# Covalent and non-covalent albumin binding of Au(i) bis-NHCs *via* post-synthetic amide modification†

**DOI:** 10.1039/d1sc01055g

**Published:** 2021-04-26

**Authors:** Sajal Sen, Mark W. Perrin, Adam C. Sedgwick, Vincent M. Lynch, Jonathan L. Sessler, Jonathan F. Arambula

**Affiliations:** Department of Chemistry, The University of Texas at Austin 105 E 24th Street A5300 Austin TX 78712-1224 USA sessler@cm.utexas.edu jfarambula@cm.utexas.edu

## Abstract

Recent decades have witnessed the emergence of Au(i) bis-N-heterocyclic carbenes (NHCs) as potential anticancer agents. However, these systems exhibit little interaction with serum proteins (*e.g.*, human serum albumin), which presumably impacts their pharmacokinetic profile and tumor exposure. Anticancer drugs bound to human serum albumin (HSA) often benefit from significant advantages, including longer circulatory half-lives, tumor targeted delivery, and easier administration relative to the drug alone. In this work, we present Au(i) bis-NHCs complexes, **7** and **9**, capable of binding to HSA. Complex **7** contains a reactive maleimide moiety for covalent protein conjugation, whereas its congener **9** contains a naphthalimide fluorophore for non-covalent binding. A similar drug motif was used in both cases. Complexes **7** and **9** were prepared from a carboxylic acid functionalized Au(i) bis-NHC (complex **2**) using a newly developed post-synthetic amide functionalization protocol that allows coupling to both aliphatic and aromatic amines. Analytical, and *in vitro* techniques were used to confirm protein binding, as well as cellular uptake and antiproliferative activity in A549 human lung cancer cells. The present findings highlight a hitherto unexplored approach to modifying Au(i) bis-NHC drug candidates for protein ligation and serve to showcase the relative benefits of covalent and non-covalent HSA binding.

## Abstract 2

Au(i) bis-N-heterocyclic carbenes (NHCs) functionalized using an amide linker were found to bind to human serum albumin (HSA) in covalent and non-covalent fashion. The solubility and *in vitro* anti-cancer activity of these new conjugates were studied.

## Graphical abstract

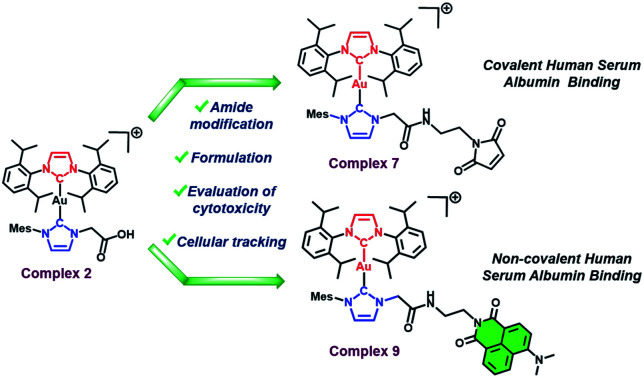

## 1. Introduction

Bis-N-heterocyclic carbene (bis-NHC) gold(i) complexes have attracted interest as potential metal-based anticancer chemotherapeutics.^1–3^ Inhibition of the mitochondria-localized enzyme thioredoxin reductase 2 (TrxR 2) has been demonstrated as the main mode of action for this class of compounds.^2,4^ However, in many instances, the phenotypic response is less than ideal. Recent findings have provided support for the notion that improved biological effects can be achieved through rational modification of classic Au(i) bis-NHC structures.^5,6^ Post-synthetic functionalization provides an attractive means for creating new Au(i) bis-NHC derivatives.^7,8^ Often this involves the use of chemical tethers to link Au(i) bis-NHCs to other moieties. However, a number of previously reported linkers, including esters and carbonates, proved susceptible to degradation in biological milieus leading to reduced efficacy.^9^ These shortcomings are providing an incentive to develop linkers that are less susceptible to hydrolysis or enzymatic scission. Here we describe a synthetic protocol that allows for the facile preparation of amide-conjugated Au(i) bis-NHCs, including species designed to bind to serum albumin (SA) through either covalent or non-covalent means. As detailed below, enhanced solubility in PBS was seen for both classes of HSA adducts; however, contrasting *in vitro* activity was found against A549 human lung cancer cells. The present work thus serves to highlight some of the advantages and disadvantages associated with HSA adduct formation.

Small-molecule anticancer drugs often lack efficient tumour targeted delivery, which results in non-specific distribution of the drug in the body. Such drugs are also plagued with short plasma circulatory lifetimes, with concomitant rapid renal clearance.^10^ Binding to serum albumin (SA) offers an attractive means of overcoming these limitations. Human serum albumin (HSA) is the most abundant protein in blood serum.^11^ It has been widely investigated as a carrier for drug molecules.^12,13^ Albumin binding with anticancer agents can be accomplished using either covalent or non-covalent means.^14^ The resulting adducts typically benefit from improved pharmacokinetics, facilitated formulation, and tumour targeted drug delivery *in vivo*.^15,16^ The benefit of SA binding is underscored by the clinical effectiveness of aldoxorubicin (INNO206; human serum albumin (HSA)-coupled doxorubicin derivative) and Abraxane® (HSA based nanoparticle encapsulation of paclitaxel) for the treatment of sarcoma,^17^ breast cancer,^18^ lung cancer,^19^ and pancreatic cancer.^20^ Several other HSA-anticancer drug conjugates are currently in clinical trials. The success of these HSA-anticancer drug conjugates is attributed to their long circulation time in blood, good tumour accumulation due to an enhanced permeability and retention (EPR) effect, and low levels of immunotoxicity.^21^

**Fig. 1:**
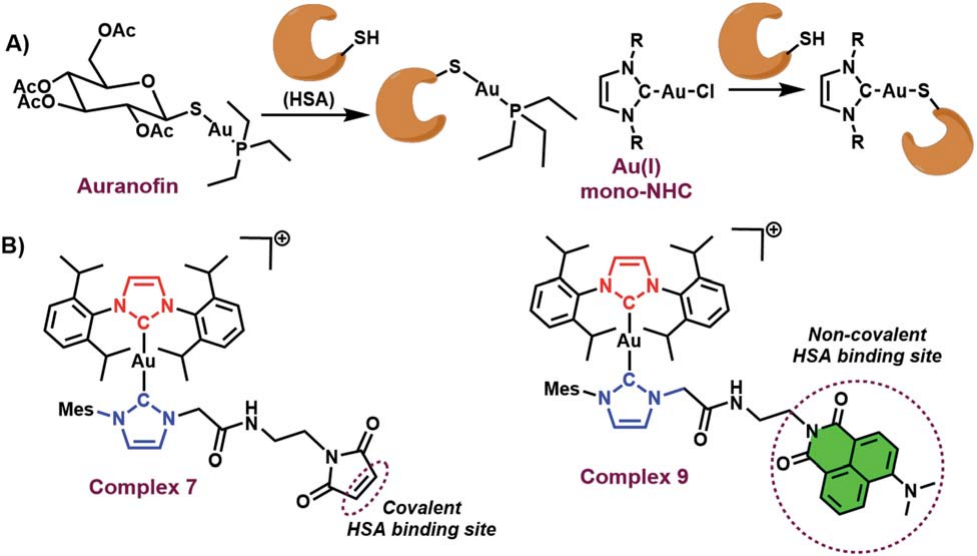
(A) Known HSA binding mode of auranofin and Au(i) mono-NHCs. (B) HSA binding modes for the Au(i) bis-NHCs **7** and **9** described here.

The binding of HSA to metal based anticancer agents has also been investigated, albeit to a lesser degree relative to small molecule organic based therapeutics. Covalent modes of binding have been reported for a number of metal drugs. In particular, HSA serves as a major carrier in the blood for auranofin,^22^ an FDA approved Au(i) drug. Mechanistic and structural studies have revealed that direct ligation of the Au(i)–PEt_3_^+^ to cys-34 on HSA is facilitated *via* exchange of the thioglucose ligand, during that binding process (Fig. 1).^22^ Recently, albumin–drug conjugates with Au(i) mono-NHCs have also been reported (see Fig. 1A).^23^ Similar to auranofin, these systems bind to HSA *via* a direct Au(i) ligation to protein based nucleophiles (see Fig. 1A).^23,24^

Unfortunately, this binding mode leads to sequestration of the gold centre *via* irreversible binding to the protein as inferred from studies involving foetal bovine serum.^4^ The effect of such binding approach may underlie the reduced *in vitro* anticancer activity seen for Au(i) mono-NHCs relative to their Au(i) bis NHC counterparts.^3–5^ Novel modes of HSA binding are thus needed to secure the metal active site to the protein without perturbing significantly the active metal centre. To date, covalent binding of intact metal complexes to HSA has been demonstrated in iridium, osmium, ruthenium, and platinum based anticancer drugs.^25–27^ To the best of our knowledge, similar strategies have not hitherto been explored in the case of Au(i) NHCs.

As true for covalent binding, non-covalent interactions with serum albumin can also aid in formulation, enhance drug stability, and lead to improved antiproliferative activity.^21^ Recently, a strategy designed to enhance non-covalent binding interactions between HSA and Au(i) bis-NHCs was reported by our lab.^8^ However, covalent binding was not achieved. More broadly, we are not aware of any systematic studies that compare the relative benefits of covalent *vs.* non-covalent binding using a similar drug motif. We thus sought to develop a synthetic method that would allow access to both covalently and non-covalently bound Au(i) bis-NHC serum albumin adducts, and do so without affecting the metal centre active site (see Fig. 1B).

## 2. Results and discussion

### 2.1 Syntheses and characterization

**Fig. 2:**
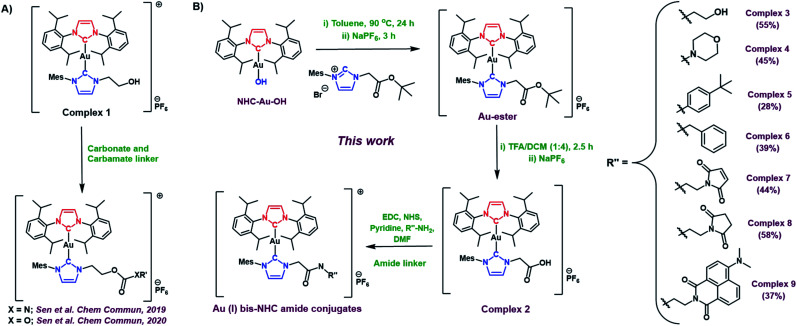
(A) Post-synthetic modification strategies developed for use with asymmetric Au(i) bis-NHCs. (B) The amide-based Au(i) bis-NHC modification approach and conjugates reported here (yields shown in brackets are after column chromatographic purification).

Prior studies of the asymmetric hydroxyethyl Au(i) bis-NHC complex **1** (Fig. 2A) cumulated in the development of both carbonate and carbamate linker strategies.^7,8^ However, carbamates and carbonates are known to be more susceptible to hydrolysis than amides in biological environments.^9^ Unfortunately, complex **1** did not allow access to Au(i) bis NHC targets containing less labile amide linkers. Thus, a new asymmetric Au(i) bis-NHC with a different functionalisable “arm” was needed. With this need in mind, we prepared a carboxylic acid analogue of complex **1**, shown here as complex **2** (Fig. 2B). In brief, **NHC-Au-OH** (Fig. 1B) was combined with an imidazolium *tert*-butyl ester and allowed to react at 90 °C overnight in toluene. This was followed by a water wash to give an ester-modified Au(i) bis-NHC (**Au-ester**, see Fig. 2B) in 75% yield. Stirring in TFA/DCM (1 : 4) mixture for 2.5 hours yielded complex **2** in 73% yield.

**Fig. 3:**
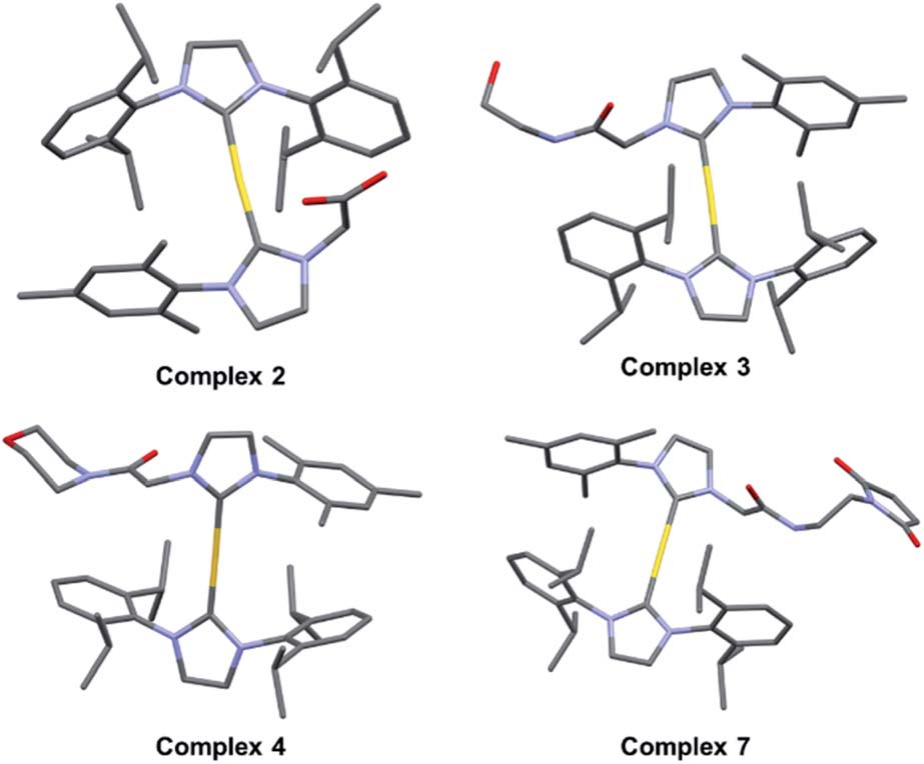
Crystal structures of complex **2** and amide conjugates **3**, **4**, and **7**.

With complex **2** in hand and using ethanolamine as a model substrate, several reaction conditions were screened in an effort to optimise amide formation (see Table S1† for details). EDC and NHS were found to be effective when dimethylformamide (DMF) was used as the solvent. This protocol was used to prepare several representative amides (complexes **3–6**, Fig. 2B) derived from both aromatic and aliphatic amines, in varying yields. Complexes **7–9**, designed for albumin binding studies (*vide infra*), were also prepared using this approach. All conjugates were fully characterized using ^1^H and ^13^C NMR spectroscopy in conjunction with HRMS analysis. Complexes **2**, **3**, **4**, and **7** were further characterized *via* single crystal X-ray diffraction analyses (Fig. 3; see ESI† for details).

### 2.2 Covalent binding between HSA and Au(i) bis-NHC

Complex **7**, containing a maleimide moiety, was designed to promote covalent binding to HSA. Maleimide derivatives of several metal and non-metal metal drug candidates have been used to achieve fast, simple, and efficient albumin-binding.^11,13,25,27–30^ HSA possesses a single free thiol group (cysteine-34) which reacts with the maleimide in a covalent fashion; this allows for the defined and selective preparation of albumin–drug conjugates without affecting the metal active site.^13^

**Fig. 4:**
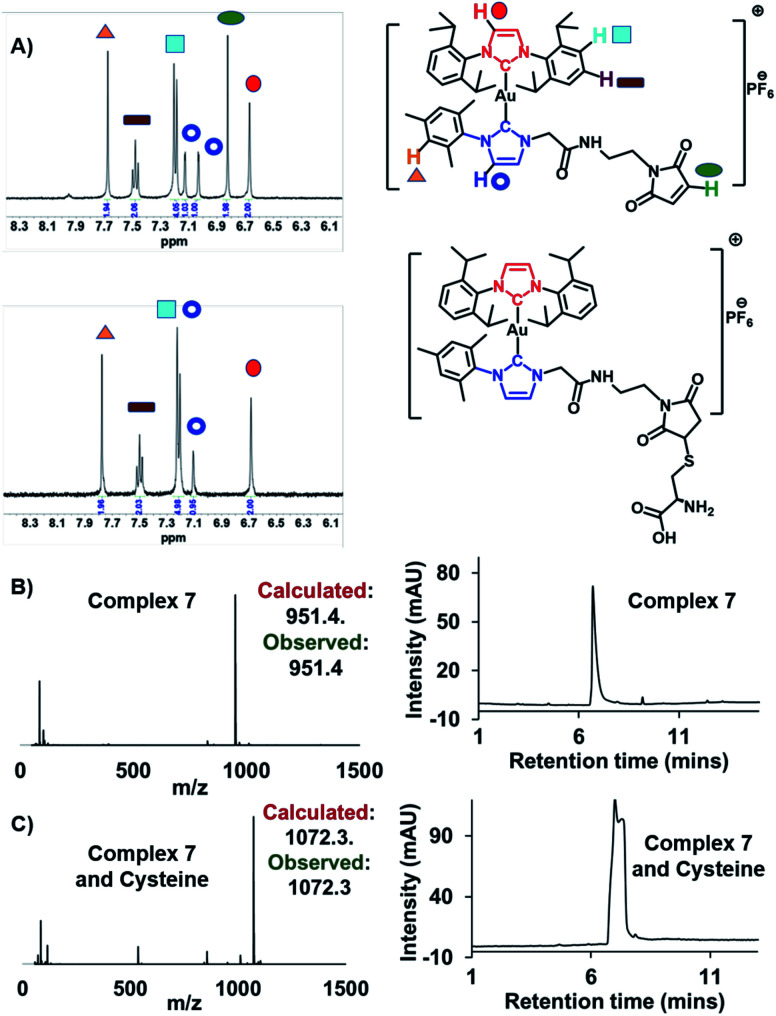
(A) Change in the ^1^H NMR spectrum in the aromatic region of complex **7** (2.25 mM) seen upon addition of 5 equivalents of cysteine in DMSO-*d*_6_/D_2_O (3 : 1) after 30 min at 37 °C. Please see the ESI† for the full spectrum. (B) Mass-spectrometric data and HPLC chromatogram corresponding to complex **7**. (C) Mass-spectrometric data and HPLC chromatogram of complex **7** post-incubation with cysteine (5 equiv.).

Complex **7** was first examined for its binding with cysteine, which was used as a model thiol reagent. Here, a 2.25 mM solution of complex **7** in DMSO-*d*_6_ (375 μL) was incubated with 5 equivalents of cysteine in D_2_O (125 μL) for 30 min. The resulting ^1^H NMR spectrum revealed an absence of the peaks at 6.9 ppm corresponding to the maleimide double bond in **7**, as would be expected upon addition of cysteine (Fig. 4A; for full spectrum, see the ESI†). Mass spectrometric (MS) and liquid chromatographic analyses from an LCMS study with complex **7** before and after cysteine addition also proved consistent with cysteine-maleimide adduct formation (Fig. 4B and C).

**Fig. 5:**
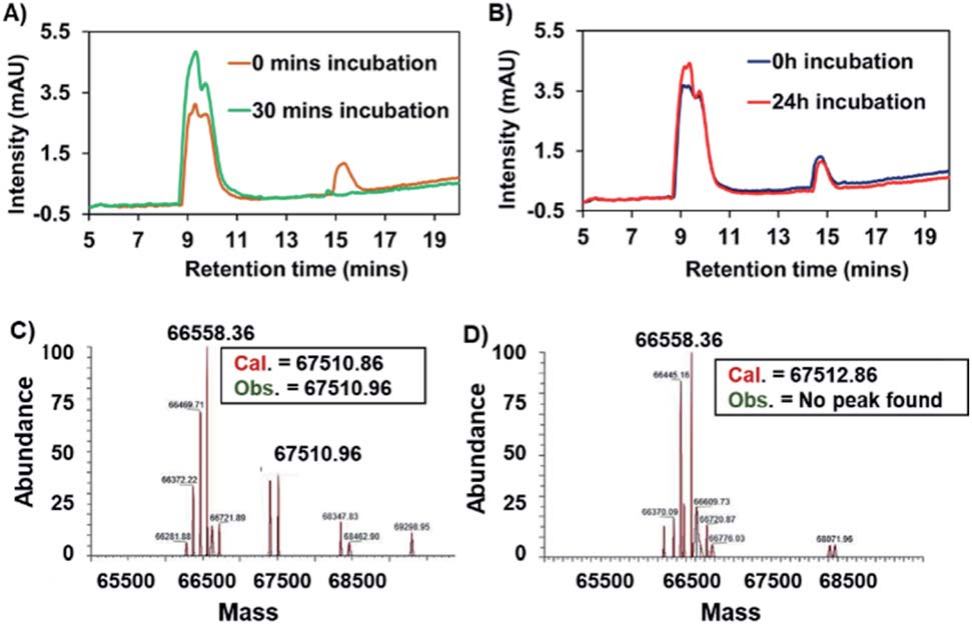
(A) HPLC chromatograms of complex **7** (100 μM) and HSA (600 μM) pre- and post-incubation. (B) HPLC chromatogram of complex **8** (100 μM) and HSA (600 μM) pre- and post-incubation. (C) Deconvolved mass-spectrum of HSA (60 μM) incubated with complex **7** (180 μM) for 2 h. (D) Deconvolved mass-spectrum of HSA (60 μM) incubated with complex **8** (180 μM) for 2 h.

Binding between HSA and complex **7** was evaluated by means of HPLC analyses. In brief, a 200 μM solution of complex **7** in PBS was incubated in the presence of 600 μM HSA (physiological concentration of HSA in human serum). Chromatograms recorded as a function of time revealed that the peak area around 15 min, which corresponds to complex **7**, diminished over the course of 1.5 h (see Fig. S1†). This reduction was accelerated at higher HSA : **7** ratios, as expected (see Fig. 5A). Using identical conditions to those used in the case of **7**, the succinimide-conjugated Au(i) bis-NHC **8** was incubated with HSA; in this case, no appreciable changes in the HPLC chromatograms were seen, even after 24 h (see Fig. 5B). Further support for the conclusion that a covalent protein–drug adduct was formed when HSA was treated with **7** came from protein molecular weight determinations. Briefly, HSA (60 μM) was co-incubated with 3 equiv. of complex **7** or complex **8** for 2 h. Post-incubation, a mass peak at 67 510.96 amu, corresponding to the target protein–drug conjugate (Fig. 5C), was seen in the case of complex **7**. As inferred from the protein mass-spectrometric analyses (Fig. 5C), complex **7** forms predominantly a 1 : 1 adduct with albumin. No adduct peaks were seen in the case of untreated HSA or HSA incubated with complex **8** (Fig. 5D and S2†). Taken in concert, these findings support the design expectation that an HSA conjugate will form in the case of complex **7**, but not in the control complex **8** that lacks a maleimide moiety.

Longer incubation of complex **7** in the presence of 5 equivalent of cysteine did not lead to any detectable NHC ligand exchange, even after 24 h, as inferred from NMR spectral studies (see Fig. S3†). The stability of complex **7** towards the nucleophilic attack from cysteine is ascribed to the steric hindrance provided by the bulky groups, *e.g.*, 1,3-diisopropylbenzyl and mesityl, present in the complex. This observation is in accord with the literature, which provides support for the notion that increasing steric hindrance reduces the rate of ligand exchange between cysteine and Au bis-NHCs.^31,32^ Further support for this conclusion came from analogous NMR spectral studies involving complex **8**; again, no discernible NHC ligand exchange in presence of cysteine was observed over the course of the study (see Fig. S4†).

### 2.3 Non-covalent binding between HSA and Au(i) bis-NHC

**Fig. 6:**
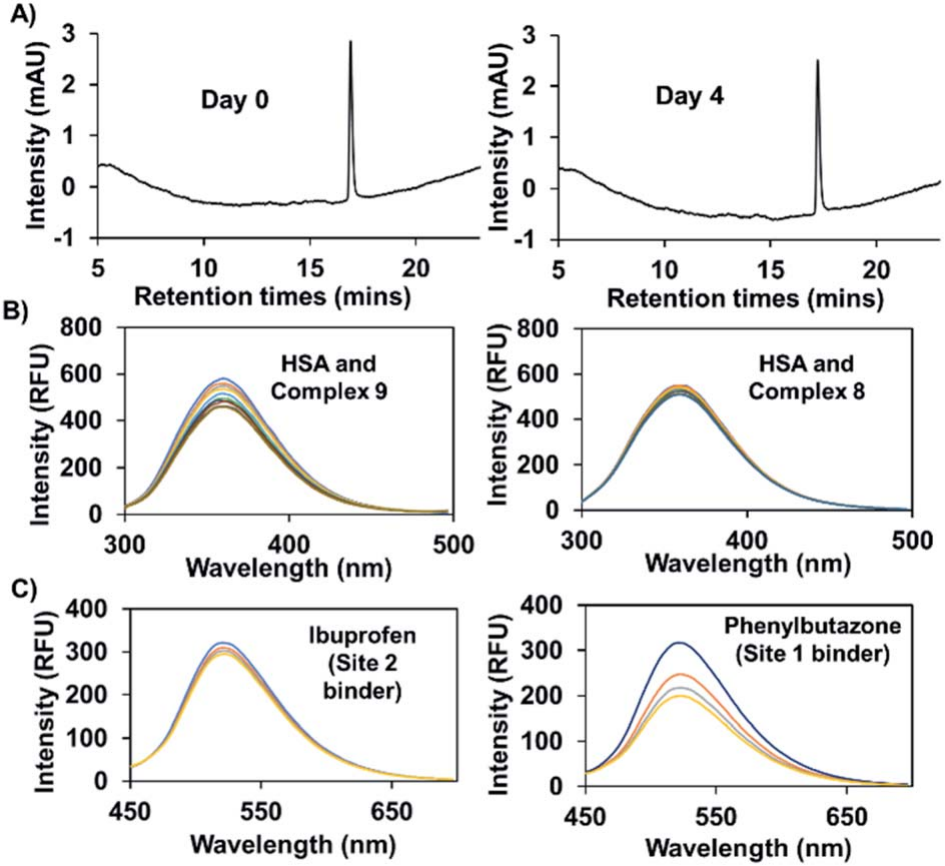
(A) HPLC chromatogram for complex **9** (100 μM) incubated in a water/methanol mixture (1 : 1) at 37 °C and sampled on day 0 and day 4. (B) Fluorescence titration of HSA (3 μM) with increasing quantities (0–3 equiv.) of complex **9** or complex **8**. (C) Fluorescence titration of a mixture of HSA (50 μM) and complex **9** (2.5 μM) with increasing quantities of phenylbutazone (site 1 binder) or ibuprofen (site 2 binder).

In previous work, and in accord with the literature,^33,34^ we found that attachment of a naphthalimide moiety to an Au(i) bis-NHC motif facilitated binding to bovine serum albumin (BSA).^8^ However, our previous studies centred around a carbonate-linked complex that was found to undergo slow hydrolysis, even *ex vivo*. Complex **9**, prepared using the present amide linking protocol, was expected to be stable against hydrolysis. As a test of this assumption, complex **9** (100 μM) was incubated in 200 μl of a water/methanol (1 : 1) mixture at 37 °C. Time-dependent HPLC analysis revealed no change in the peak area corresponding to complex **9**, even after 4 days (see Fig. 6A). Under analogous conditions our previously reported carbonate bonded naphthalimide system proved unstable,^8^ leading us to infer that complex **9** would be suitable for use *in vitro*.

Efforts were then made to probe the putative interactions between complex **9** and serum albumin. It was found that the fluorescence intensity of complex **9** increased in the presence of bovine and human serum albumin as compared to what was seen in PBS alone (Fig. S5†). Various fluorescence titrations were then performed using solutions of HSA (3 μM) to which increasing quantities of complex **9** were added. The intrinsic fluorescence intensity of HSA (*λ*_ex_ = 290 nm), ascribed to the presence of tryptophan subunits, is quenched in a concentration dependent manner in the presence of complex **9.** In contrast, complexes **2** and **8** for which little appreciable binding to HSA is expected did not induce appreciable quenching (see Fig. 6B and S6†). By applying a Stern–Volmer analysis, a bimolecular quenching constant (*k*_q_ = (3.0 ± 0.2) × 10^12^ M^−1^ s^−1^) was derived (see Fig. S7†). This value is consistent with static binding between complex **9** and HSA.^35^

To obtain further insights into the possible site(s) in HSA to which complex **9** is presumably binding, competition experiments with site-specific inhibitors of HSA were carried out. Here, increasing quantities of phenylbutazone (site 1 binder) or ibuprofen (site 2 binder) were added to HSA (50 μM) solutions containing complex **9** (2.5 μM). A significant decrease in the fluorescence intensity was seen in the case of phenylbutazone, but not ibuprofen (see Fig. 6C). On this basis, we infer that complex **9** binds predominantly in site 1 of HSA.^34^

### 2.4 Enhanced aqueous solubility owing to HSA interaction

**Fig. 7:**
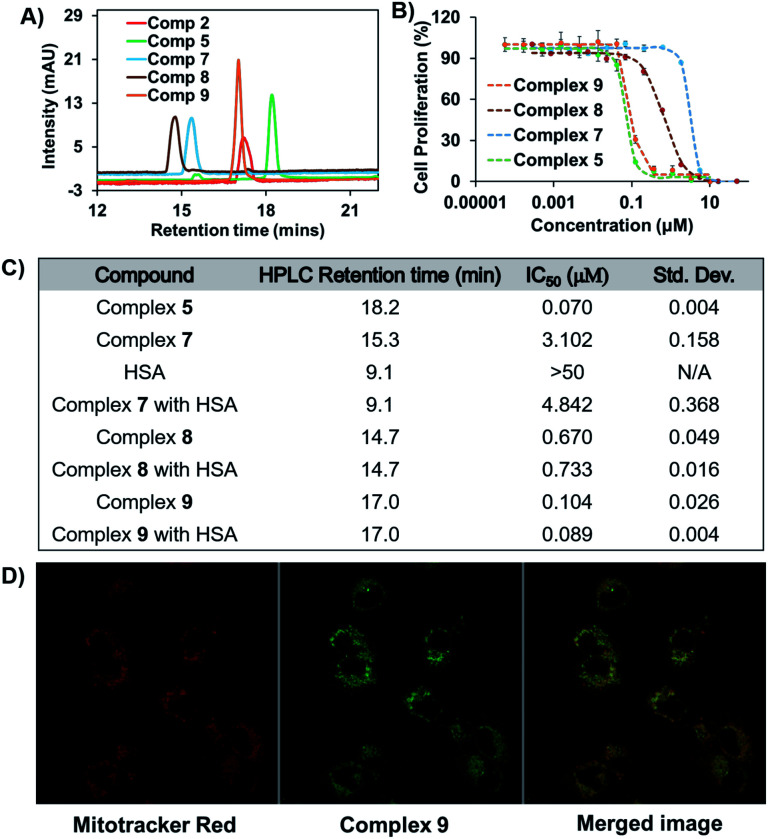
(A) HPLC chromatograms of selected Au(i) bis-NHC amide conjugates showing differences in the retention time. (B) Cell proliferation profile of amide conjugates against the A549 lung cancer cell line. (C) Cytotoxicity values obtained from 3 day MTT assays against A549 cells (experiments repeated in triplicate). (D) Confocal microscopy images obtained post-incubation with complex **9** in A549 cells.

Poor aqueous solubility can often limit the development of otherwise promising anticancer drug candidates.^36^ However, anticancer drugs clinically formulated with exogenous albumin prior to administration are viewed as a means to circumvent this roadblock.^14,37^ In fact, treatment of both complexes **7** and **9** with HSA was found to increase their solubility in PBS. Specifically, 150 μM solutions of complexes **7** and **9** (2% DMSO in PBS containing 600 μM HSA) remained transparent for up to 96 h (see Fig. S8†). However, in the absence of HSA neither complex could be fully solubilized in 2% DMSO in PBS and both were found to precipitate out within 15 min of initial mixing. Under analogous conditions and concentrations, complex **8** (as well as **2**) was found to precipitate within 12 h of formulating with HSA (see Fig. S8†). This contrast is noteworthy since complex **8** is no less hydrophilic than complex **7** or **9** as inferred from HPLC studies (Fig. 7A and C). Furthermore, complex **7** could be formulated at an even higher concentration (300 μM) by doubling the DMSO concentration to 4% in PBS while keeping the HSA concentration constant at 600 μM. Unfortunately, the solubility of complex **9** could not be increased in this way, presumably because of its relatively more hydrophobic nature (Fig. 6A). These solubility studies thus underscore the relative merits of the covalent and non-covalent binding approaches to creating Au(i) bis-NHC HSA adducts.

### 2.5 Cytotoxicity and cellular colocalization studies in A549 cells

The *in vitro* antiproliferative activity of representative amide conjugates was evaluated by means of MTT assays carried out using the A549 lung cancer cell line. With the exception of complex **7**, the antiproliferative response was positively corelated to the complex hydrophobicity (*i.e.*, the greater the anticancer response, the greater the HPLC retention time) (Fig. 7A–C).

A relatively low cytotoxic response was seen for complex **7**, which is attributed to its rapid and strong binding to the foetal bovine serum present in the medium. Evidence for this proposed binding came from HPLC studies wherein complex **7** was incubated with RPMI-1640 media containing 10% FBS (see Fig. S9†). The cytotoxicity proved even lower when complex **7** was pre-incubated with HSA (higher IC_50_ value, Fig. 7C). Similar reductions in cytotoxicity post-HSA incubation have previously been reported for a few covalently HSA-bound anticancer drugs.^38,39^ Further elongation of the drug incubation time (5 days) does not lead to a dramatic change in the antiproliferative activity of complex **7** (see Fig. S10,† IC_50_ = ∼2.3 μM).

To probe further the putative role of HSA in reducing the cytotoxicity of complex **7***in vitro*, an analogous pre-incubation experiment with HSA was carried out using its congener, complex **8**. Complex **8** contains a succinimide moiety in place of the maleimide group present in complex **7**. As such, it does not form a covalent adduct with HSA (*vide supra*). The corresponding MTT results revealed that pre-incubation with HSA has a minimal effect on the cytotoxicity of complex **8** (Fig. 7C and S11†) in contrast to what is true for complex **7**. We thus conclude that covalent binding to HSA provides a means to modulate the *in vitro* cytotoxicity of Au bis-NHC amide derivatives. On the other hand, complex **9**, which benefits from a non-covalent interaction with albumin, does not induce an appreciable reduction in cytotoxicity, even after incubation with 6 equivalents of HSA for 1.5 h (see Fig. S11†). This is also in accord with previously reported studies in the literature.^21^

Complex **9** could be exploited to effect cellular tracking *via* confocal microscopy. Au(i) mono-carbenes with naphthalimide moiety have been previously reported to localize in lysosome.^40^ In contrast, free standing naphthalimide fluorophores typically colocalize in the nucleosome.^41^ Finally, Au(i) bis-NHCs act as mitochondria-targeting chemotherapeutics.^1^ Therefore, the behaviour of complex **9** was difficult to predict *a priori*. To gain insight into the cellular localisation of complex **9**, confocal microscopy studies were carried out. Merged images obtained 4 h post incubation with complex **9** and Mitotracker® Red in A549 cells lead to the conclusion that it colocalises predominantly in the mitochondria (Fig. 7D).

## 3. Conclusions

In conclusion, we have developed a strategy for post-synthetic amide conjugation that relies on the use of the new Au(i) bis-NHC complex (**2**) bearing a carboxylic acid functional group. The scope of this reaction protocol was tested against aliphatic and aromatic amines, both of which could be linked successfully. Covalent and non-covalent binding to albumin was then effected using appropriately chosen amide Au(i) bis-NHCs. HSA binding served to enhance the solubility of complexes **7** and **9** in PBS containing HSA. This permitted much higher concentrations of both complexes to be solubilized in water than could otherwise be achieved for the control complexes **2** and **8**, both of which precipitated out from solution under analogous conditions notwithstanding the fact that both are characterized by similar hydrophilic profiles. In the case of complex **7**, a system designed to support covalent attachment to HSA, albumin binding resulted in a reduction in cytotoxicity. This result is in line with other albumin conjugates involving robust linkers.^38,39^ This proof-of-concept work lays the foundation for future Au(i) bis-NHC protein conjugates involving labile linkers as a means to increase the cytotoxicity but maintain the beneficial attributes of protein conjugation.

In contrast to the covalent nature of HSA binding with **7**, complex **9** was designed to support non-covalent binding to HSA. The resulting complex retained its strong antiproliferative activity in the presence of HSA as evidenced from *in vitro* studies. This non-covalent complex, which bears a naphthalimide fluorophore, also allowed studies of subcellular localization to be carried out. Confocal microscopic studies involving complex **9** revealed its predominant colocalization inside the mitochondria of A549 cells, a finding consistent with the prior literature. However, due to inherent limitations of the confocal microscopy technique,^42^ and the environmentally-sensitive fluorescent nature of naphthalimide fluorophores,^43,44^ we believe it would prove beneficial to probe further the cellular fate of Au bis-NHC complexes using other imaging techniques. Studies along these lines are currently planned.

The present work details two readily workable approaches to creating albumin adducts comprising Au(i) bis-NHCs as active payloads. The binding modes described here do not affect the metal centre active site, as normally occurs for auranofin or Au(i) mono-NHCs. Our studies also highlight the relative merits of covalent and non-covalent attachment to serum proteins in the case of Au(i) bis-NHCs. This work is thus likely to broaden the scope of rationally designed Au(i) bis-NHCs *via* protein conjugation as tumour-targeted chemotherapeutics.

## Author contributions

S. S. and M. P. performed the synthesis and characterized the compounds. S. S. carried out the *in vitro* assays, HPLC, fluorescence, and solubility experiments. S. S., and M. L. analyzed the *in vitro* data. V. M. L. solved the X-ray structures of compounds. S. S., A. C. S., J. F. A., and J. L. S. designed the project, analyzed the results, and wrote the manuscript.

## Conflicts of interest

There are no conflicts to declare.

## Supplementary Material

SC-012-D1SC01055G-s001

SC-012-D1SC01055G-s002

## References

[cit1] Berners-Price S. J., Filipovska A. (2011). Metallomics.

[cit2] Hickey J. L., Ruhayel R. A., Barnard P. J., Baker M. V., Berners-Price S. J., Filipovska A. (2008). J. Am. Chem. Soc..

[cit3] Porchia M., Pellei M., Marinelli M., Tisato F., Del Bello F., Santini C. (2018). Eur. J. Med. Chem..

[cit4] McCall R., Miles M., Lascuna P., Burney B., Patel Z., Sidoran K. J., Sittaramane V., Kocerha J., Grossie D. A., Sessler J. L., Arumugam K., Arambula J. F. (2017). Chem. Sci..

[cit5] Mora M., Gimeno M. C., Visbal R. (2019). Chem. Soc. Rev..

[cit6] Sen S., Hufnagel S., Maier E. Y., Aguilar I., Selvakumar J., DeVore J. E., Lynch V. M., Arumugam K., Cui Z., Sessler J. L., Arambula J. F. (2020). J. Am. Chem. Soc..

[cit7] Sen S., Li Y., Lynch V., Arumugam K., Sessler J. L., Arambula J. F. (2019). Chem. Commun..

[cit8] Sen S., Perrin M. W., Sedgwick A. C., Dunsky E. Y., Lynch V. M., He X.-P., Sessler J. L., Arambula J. F. (2020). Chem. Commun..

[cit9] Rautio J., Kumpulainen H., Heimbach T., Oliyai R., Oh D., Järvinen T., Savolainen J. (2008). Nat. Rev. Drug Discovery.

[cit10] Larsen M. T., Kuhlmann M., Hvam M. L., Howard K. A. (2016). Mol. Cell. Ther..

[cit11] Liu Z., Chen X. (2016). Chem. Soc. Rev..

[cit12] Elsadek B., Kratz F. (2012). J. Controlled Release.

[cit13] Kratz F. (2008). J. Controlled Release.

[cit14] Hoogenboezem E. N., Duvall C. L. (2018). Adv. Drug Delivery Rev..

[cit15] Ohnuma T., Ohnuma T., Kavy S., Bhardwaj S., Holland J. F. (1980). Br. J. Cancer.

[cit16] Yamasaki K., Chuang V. T. G., Maruyama T., Otagiri M. (2013). Biochim. Biophys. Acta, Gen. Subj..

[cit17] Cranmer L. D. (2019). OncoTargets Ther..

[cit18] Miele E., Spinelli G. P., Miele E., Tomao F., Tomao S. (2009). Int. J. Nanomed..

[cit19] Green M. R., Manikhas G. M., Orlov S., Afanasyev B., Makhson A. M., Bhar P., Hawkins M. J. (2006). Ann. Oncol..

[cit20] Von Hoff D. D., Ervin T., Arena F. P., Chiorean E. G., Infante J., Moore M., Seay T., Tjulandin S. A., Ma W. W., Saleh M. N., Harris M., Reni M., Dowden S., Laheru D., Bahary N., Ramanathan R. K., Tabernero J., Hidalgo M., Goldstein D., Van Cutsem E., Wei X., Iglesias J., Renschler M. F. (2013). N. Engl. J. Med..

[cit21] Zheng Y. R., Suntharalingam K., Johnstone T. C., Yoo H., Lin W., Brooks J. G., Lippard S. J. (2014). J. Am. Chem. Soc..

[cit22] Pratesi A., Cirri D., Ciofi L., Messori L. (2018). Inorg. Chem..

[cit23] Matos M. J., Labão-Almeida C., Sayers C., Dada O., Tacke M., Bernardes G. J. L. (2018). Chem.–Eur. J..

[cit24] Ferraro G., Gabbiani C., Merlino A. (2016). Bioconjugate Chem..

[cit25] Moon S., Hanif M., Kubanik M., Holtkamp H., Söhnel T., Jamieson S. M. F., Hartinger C. G. (2015). ChemPlusChem.

[cit26] Zhang P., Huang H., Banerjee S., Clarkson G. J., Ge C., Imberti C., Sadler P. J. (2019). Angew. Chem., Int. Ed..

[cit27] Mayr J., Heffeter P., Groza D., Galvez L., Koellensperger G., Roller A., Alte B., Haider M., Berger W., Kowol C. R., Keppler B. K. (2017). Chem. Sci..

[cit28] Pichler V., Mayr J., Heffeter P., Dömötör O., Enyedy É. A., Hermann G., Groza D., Köllensperger G., Galanksi M., Berger W., Keppler B. K., Kowol C. R. (2013). Chem. Commun..

[cit29] Hanif M., Nazarov A. A., Legin A., Groessl M., Arion V. B., Jakupec M. A., Tsybin Y. O., Dyson P. J., Keppler B. K., Hartinger C. G. (2012). Chem. Commun..

[cit30] Kratz F., Warnecke A., Scheuermann K., Stockmar C., Schwab J., Lazar P., Drückes P., Esser N., Drevs J., Rognan D., Bissantz C., Hinderling C., Folkers G., Fichtner I., Unger C. (2002). J. Med. Chem..

[cit31] Tolbatov I., Coletti C., Marrone A., Re N. (2020). Inorg. Chem..

[cit32] Dos Santos H. F., Vieira M. A., Sánchez Delgado G. Y., Paschoal D. (2016). J. Phys. Chem. A.

[cit33] Cheng H., Zou T., Xu Y., Wang Y., Wu A., Dai J., Zhang Y., Liu Y. (2016). Luminescence.

[cit34] Chai X., Han H. H., Sedgwick A. C., Li N., Zang Y., James T. D., Zhang J., Le Hu X., Yu Y., Li Y., Wang Y., Li J., He X. P., Tian H. (2020). J. Am. Chem. Soc..

[cit35] Liu J., He Y., Liu D., He Y., Tang Z., Lou H., Huo Y., Cao X. (2018). RSC Adv..

[cit36] Kalepu S., Nekkanti V. (2015). Acta Pharm. Sin. B.

[cit37] Bianco I., Garro A. G., Beltramo D. M., Alasino R. V., Leonhard V., Heredia V. (2011). Int. J. Nanomed..

[cit38] Warnecke A., Fichtner I., Garmann D., Jaehde U., Kratz F. (2004). Bioconjugate Chem..

[cit39] Kratz F., Beyer U., Collery P., Lechenault F., Cazabat A., Schumacher P., Falken U., Unger C. (1998). Biol. Pharm. Bull..

[cit40] Groves L. M., Williams C. F., Hayes A. J., Ward B. D., Isaacs M. D., Symonds N. O., Lloyd D., Horton P. N., Coles S. J., Pope S. J. A. (2019). Dalton Trans..

[cit41] Cao C., Wei P., Li R., Zhong Y., Li X., Xue F., Shi Y., Yi T. (2019). ACS Sens..

[cit42] Pawley J. B. (1991). Scanning.

[cit43] Banerjee S., Veale E. B., Phelan C. M., Murphy S. A., Tocci G. M., Gillespie L. J., Frimannsson D. O., Kelly J. M., Gunnlaugsson T. (2013). Chem. Soc. Rev..

[cit44] Dong H. Q., Wei T. B., Ma X. Q., Yang Q. Y., Zhang Y. F., Sun Y. J., Shi B. B., Yao H., Zhang Y. M., Lin Q. (2020). J. Mater. Chem. C.

