# Supplementary material for: Covalent and non-covalent albumin binding of Au(i) bis-NHCs via post-synthetic amide modification
Source: Chem Sci. 2021 Apr 26;12(21):7547–53. doi: 10.1039/d1sc01055g (PMC8171490; doi:10.1039/d1sc01055g)
Supplement: SC-012-D1SC01055G-s001 [file SC-012-D1SC01055G-s001.pdf]

Electronic Supplementary Material (ESI) for Chemical Science.
This journal is © The Royal Society of Chemistry 2021

Electronic Supplementary Information for:

Covalent and non-covalent albumin binding of Au(I) bis-NHCs via post-synthetic amide modification

Sajal Sen, Mark W. Perrin, Adam C. Sedgwick, Vincent M. Lynch, Jonathan L. Sessler,* Jonathan F. Arambula*

Department of Chemistry, The University of Texas at Austin, 105 E 24th Street A5300, Austin, TX 78712-1224, United States.

Corresponding author e-mails: sessler@cm.utexas.edu, jfarambula@cm.utexas.edu.

Table of Contents

1. Materials and Methods S2
2. Synthesis S4
    2.1 Synthesis of the key *tert*-butyl mesityl imidazolium ester "Imid Ester": S5
    2.2 Synthesis of the intermediate referred to as "Au-ester": S6
    2.3 Synthesis of complex 2: S7
    2.4 General synthesis of products referred to as "Au-amides": S8
    2.4a Synthesis of complex 3: S9
    2.4b Synthesis of complex 4: S10
    2.4c Synthesis of complex 5: S11
    2.4d Synthesis of complex 6: S12
    2.4e Synthesis of complex 7: S13
    2.4f Synthesis of complex 8: S14
    2.4g Synthesis of complex 9: S15
3. Results from Analytical Experiments: S16
4. Results from Biological Experiments S20
5. Crystallographic Analyses S21
6. NMR spectra: S26
7. HRMS Results: S48
9. References S51

1. Materials and Methods

All chemical reactions were conducted under a nitrogen atmosphere using Schlenk techniques unless otherwise noted. The glassware was oven dried at 120 °C before use. All materials were obtained from commercial sources at the highest purity available and used without further purification. Chloro (dimethylsulfide) gold(I) was purchased from Sigma-Aldrich. Solvents were either dried with a solvent purification system (dichloromethane (DCM), acetonitrile (ACN), methanol) or dried over molecular sieves (toluene) (3 Å) and degassed prior to use. The precursors referred to as Au-OH and naphthalimide-amine (structures shown in the synthetic experimental) were synthesized according to previous protocols.[1,2] N-(2-Aminoethyl)maleimide trifluoroacetate salt and 1-(2-aminoethyl)pyrrolidine-2,5-dione hydrochloride were purchased from Sigma-Aldrich and Oakwood Chemicals, respectively.

Analytical studies: The reported ^{1}H NMR and ^{13}C NMR spectra were measured on Varian Inova spectrometers at The University of Texas at Austin using either CD_2Cl_2 or DMSO-d_6 as the deuterated solvents. Chemical shifts are reported relative to the residual solvent proton signals. For the spin multiplicities the following abbreviations were used: s (singlet), d (doublet), t (triplet) and m (multiplet), as well as appropriate combinations of these. Coupling constants for protons (*J*) are given in Hertz (Hz). The NMR spectra were analyzed using the software *MestReNova v.10.0.2-15465* (Mestrelab Research S.L.). All deuterated solvents were purchased from Cambridge Isotope Laboratories. High-resolution electrospray ionization (ESI) mass spectra were recorded on a VG ZAB2E instrument or VG AutoSpec apparatus. Column chromatography was performed on Sorbent silica gel (40-63 µm). Analytical thin layer chromatography (TLC) analyses were carried on glass-backed silica gel plates (200 µm, Sorbent Technologies). Fluorescence spectroscopic measurements were made using an Agilent Cary Eclipse fluorescence spectrofluorometer. Analytical and semi-preparative RP-HPLC were performed on a Thermo Scientific Dionex Ultimate 3000 instrument equipped with a PDA detector. The analytical column was a Syncronis C18 column, 5 µm, 4.6 x 250 mm (Thermo Scientific); the mobile phase containing 0.1% acetic acid consisted of an increasing gradient from 10% acetonitrile/water to 99% acetonitrile/water over 2-20 min at a flow rate of 1.2 ml/min. HSA was purchased from Sigma-Aldrich and used without further purification. All fluorescence measurements for the titrations against HSA were recorded from 300 to 500 nm with an excitation wavelength of 295 nm. The emission and excitation slit widths were fixed at 5 mm. The approximate concentration of HSA in the resulting stock solutions was determined spectrophotometrically using a molar extinction coefficient of 35,700 M^{-1} cm^{-1} at 280 nm. For the titrations themselves, 3 mM solutions of the complex in question was used as the working solution with 1 µl aliquots being used for each addition into a 3 ml volume of the HSA stock solution. This helps to avoid decreases in the emission signal intensity due to dilution. Respective blanks were subtracted from the spectra. To avoid inner filter effects originating from complex **9**, the fluorescence titration was performed using a low concentration of HSA (3 µM). Furthermore, inner filter effect corrections for the observed fluorescence intensities were made according to previous reports[3–5] in an effort to minimize any possible error. Stern-Volmer analyses were carried out using the resulting corrected fluorescence intensity values in accord with the following equation: [6–8]

$$\frac{F_0}{F} = K_{SV}[\mathrm{Q}] + 1 = k_q\tau_0 + 1$$

where, F_0 and F are the fluorescence intensities in the absence and presence of the quencher Q under study (i.e., complex **2**, complex **8**, or complex **9**), K_{SV} is the Stern–Volmer quenching constant (measuring the efficiency of quenching), k_q is the bimolecular rate constant of the quenching reaction and τ_0 is the average integral fluorescence life time of tryptophan, which was taken from the literature as being ~10^{-8} s.[6-

[8] The binding constants and the number of binding site were obtained using the modified Stern-Volmer equation:[6-8]

$$\log\left(\frac{F_0}{F}-1\right)=\log k_b+n\log[\mathrm{Q}]$$

where, k_b is the binding constant, n is the number of binding sites, [Q] is the concentration of quencher under study (i.e. complex **2**, **8**, or **9**). In accord with the literature, the nature of the quenching process (dynamic or static) can be determined from the bimolecular rate constants obtained for each complex (maximum scattering collision quenching rate constant : 2.0×10^{10} L mol^{-1} s^{-1}).[6–8]

Cell proliferation studies: A549 cells were harvested and seeded into 96-well culture plates (Costar 07-200-90) in 100 μL of culture medium. The cells were allowed to incubate overnight at 37 ºC in the presence of 5% CO_2. A549 cells were seeded at a density of 1500 cells/well. The next day, appropriate serial dilutions of drug stocks in culture media were made. To each well of a 96 well plate was added 100 μL of the appropriate solution. After a total of three days, a 50 μL aliquot of 3 mg/mL tetrazolium dye, 3-(4,5-dimethylthiazol-2-yl)-2,5-diphenyltetrazolium bromide (Alfa Aesar L11939), dissolved in culture medium without fetal bovine serum (FBS), was added to each well, followed by a 4 h incubation period at 37 ºC. After removal of the medium, the resulting formazan was dissolved in 50 μl DMSO and the respective absorbances were measured at 560–650 nm using a microplate reader (Molecular Devices, Sunnyvale, CA). Absorbance values were corrected for background and then normalized to wells containing untreated cells to allow for plate-to-plate comparisons. The resulting dose response curves were subjected to linear regression analysis (Origin by OriginLab, Inc.) to determine the IC_{50} values. The data are shown as mean inhibition of proliferation or growth as a percentage of control cells and are from 3 replicate experiments. The maximum concentration of DMSO used was 0.1%. This concentration of DMSO was determined to be non-toxic in separate control experiments. To evaluate the cytotoxicity of HSA-treated complex **7** and complex **9**, 200 μM of complex **7** or 100 μM of complex **9** were incubated with 40 mg/ml (~600 μM) HSA at 37° C for 1.5 h (final DMSO conc. 2%). The stock solution of complex **7** was further diluted 2-fold in RPMI media such that the maximum drug concentration used was 50 μM for the dose dependent MTT assay. For complex **9**, the maximum drug concentration was 10 μM.

Confocal fluorescence microscopy studies: Tumor cells were harvested and seeded at a density of 2 x 10^5 cells/dish in 35 mm dishes containing a poly-D lysine coated 10 mm glass diameter (Mat Tek P35GC-1.5- 10-C) overnight. Cells were then incubated with respective doses of complex **9** at 37 ºC for 4 h. Post incubation, the media was removed and the cells were washed (2x) with PBS. To the cells was added a PBS solution containing 50 nM Mitotracker® Red FM (Lifetech M22425) for 30 min at 37 ºC. After incubation, the PBS solution containing the dye was removed and the cells were washed with PBS (2x). The cells were then subject to fluorescent imaging using a Zeiss LSM 710 laser scanning confocal microscope with a Plan-Apo 63x/1.4 oil objective. The green channel was excited with a 405 nm laser, and the emission was detected spectrally from 482 - 555 nm. The red channel was excited with a 561 nm laser, and the emission was detected spectrally from 573 - 639 nm.

Protein mass spectrometric analysis: HSA (60 μM) was incubated with complex **7** or complex **8** (3 equiv.) for 2 h at 37 °C. The resulting samples along with an untreated HSA sample were submitted for protein mass spec analysis to The University of Texas at Austin Proteomics Facility. Experiments were conducted using an Optimize Technologies protein trap in-line with an Thermo Orbitrap Fusion. The data was collected using the ion trap detector and deconvoluted using the Thermo Protein Deconvolution software.

Common abbreviations used:

DCM = dichloromethane. **DEE** = diethyl ether. **TEA** = triethylamine. **ACN** = acetonitrile. **TFA** = trifluoroacetic acid. **HSA** = Human serum albumin.

2. Synthesis

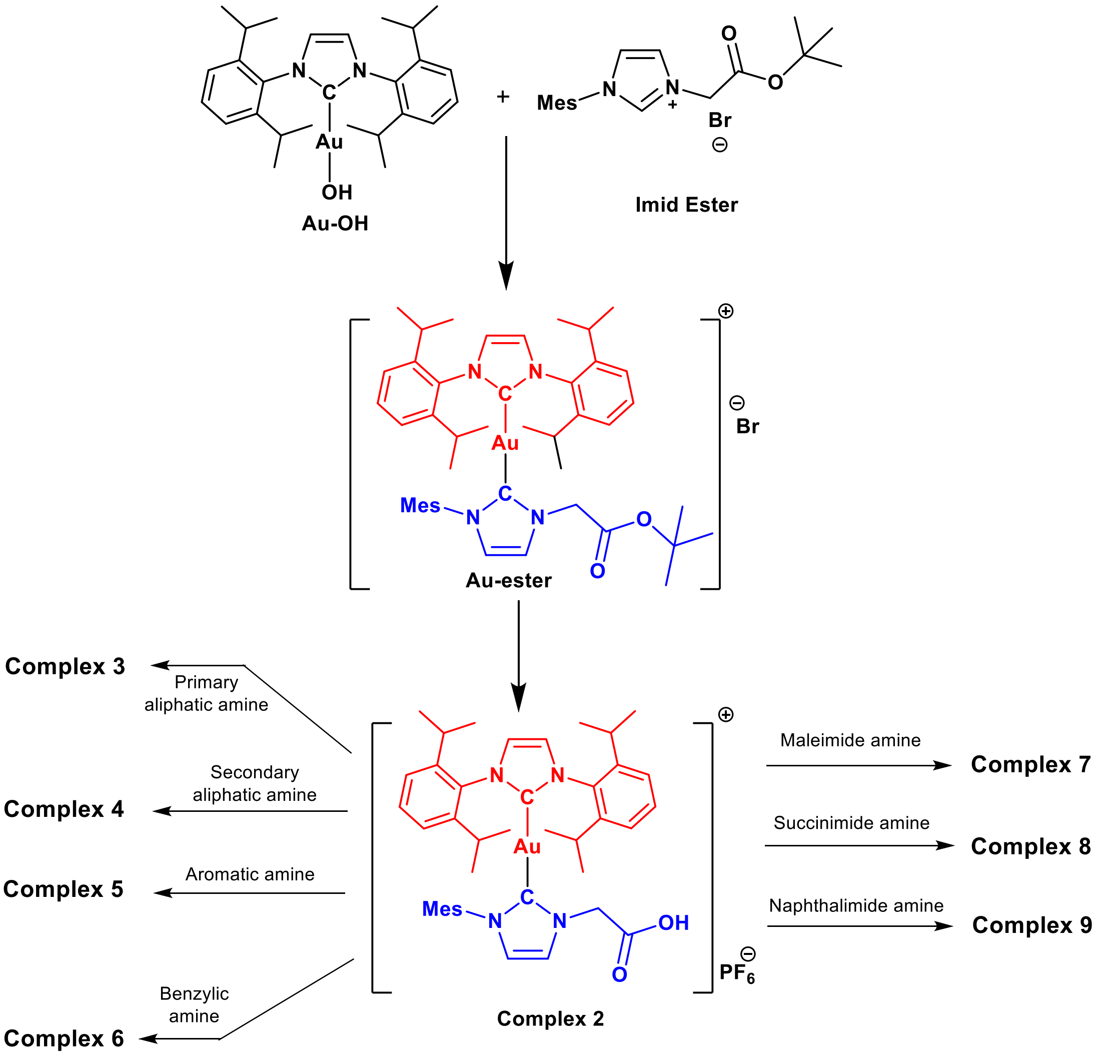

Scheme S1: Schematic diagram showing the preparation of the Au-Bis NHC amide conjugates of this study.

2.1 Synthesis of the key *tert*-butyl mesityl imidazolium ester "Imid Ester":

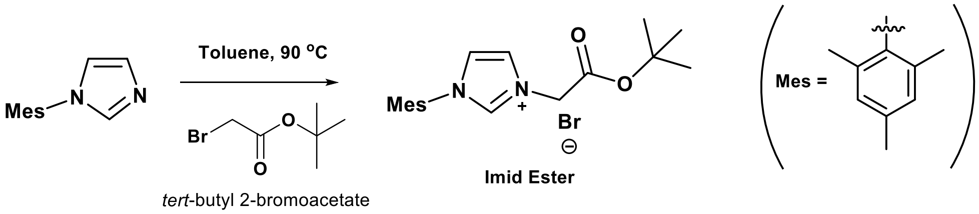

Mesityl imidazole (300 mg, 1.61 mmol, 1 equiv.) and *tert*-butyl 2-bromoacetate (0.195 ml, 3.54 mmol) were combined in an oven-dried two-neck round-bottomed flask. Dry toluene (4 ml) was added to the mixture and the resulting solution was stirred for 24 h under nitrogen atmosphere. A white solid precipitated out which was collected via filtration and washed several times with toluene, followed by hexanes. Finally, the precipitate was left to dry under air.

Yield: 605 mg, 98%.

^{1}H NMR (500 MHz, DMSO-*d*$_6$) δ 9.45 (dd, *J* = 3.4, 1.6 Hz, 1H), 8.05 (q, *J* = 1.8 Hz, 1H), 7.96 (t, *J* = 1.8 Hz, 1H), 7.17 (s, 2H), 5.26 (d, *J* = 2.1 Hz, 2H), 2.35 (s, 3H), 2.03 (s, 6H), 1.47 (s, 9H).

^{13}C NMR (126 MHz, DMSO-*d*$_6$) δ 165.9, 140.8, 139.2, 134.6, 131.5, 129.7, 129.4, 128.7, 125.8, 124.9, 124.0, 83.7, 50.9, 28.1, 21.1, 17.3.

2.2 Synthesis of the intermediate referred to as "Au-ester":

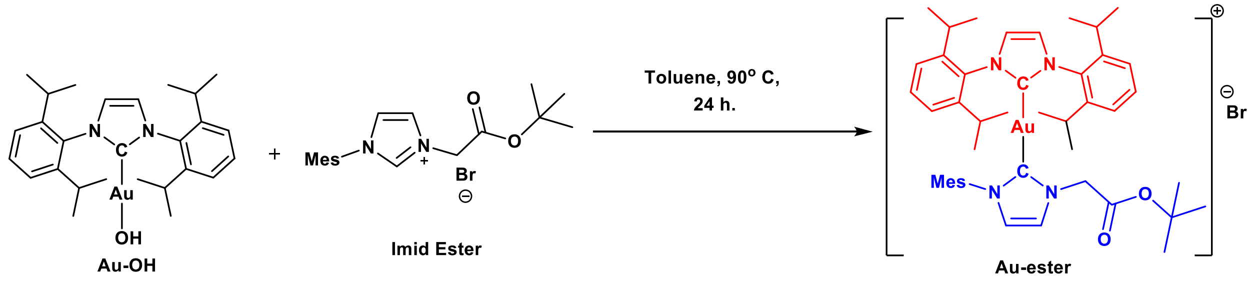

Au-OH (250 mg, 0.414 mmol, 1 equiv.) and Imid Ester (157 mg, 1 equiv.) were combined in an oven-dried two neck round-bottomed flask and kept under vacuum for 15 mins. After refilling the flask with nitrogen, dry toluene (10 ml) was added and the mixture was kept for stirring at 90 °C for 24 h which led to an appearance of a white precipitate. The precipitate was collected via filtration and washed with toluene followed by hexanes. After collecting the precipitate in a scintillation vial, 5 ml HPLC grade water was added to it and the mixture was left to stir for another 15 mins to remove any residual imidazolium salt. Finally, the mixture was filtered again and the resulting solid was washed with water and diethyl ether. The precipitate was collected and dried under vacuum to afford the Au-ester as a white crystalline solid.

Yield: 299 mg, 0.310 mmol, 75%.

ESI-HRMS (acetonitrile) (m/z): Calculated for $[C_{45}H_{60}AuN_4O_2]^+$: 885.4382. Observed : 885.4377.

^{1}H NMR (500 MHz, DMSO-d_6) δ 8.06 (s, 2H), 7.65 – 7.57 (m, 3H), 7.34 (d, *J* = 7.6 Hz, 5H), 6.79 (s, 2H), 4.40 (s, 2H), 2.35 (q, *J* = 6.7, 5.3 Hz, 7H), 1.56 (s, 6H), 1.37 (s, 9H), 1.16 (d, *J* = 6.8 Hz, 12H), 1.05 (d, *J* = 6.9 Hz, 12H).

^{13}C NMR (126 MHz, DMSO-d_6) δ 185.9, 183.3, 165.9, 145.4, 138.9, 134.2, 133.8, 131.0, 129.5, 129.4, 128.7, 125.8, 125.5, 123.9, 82.9, 51.6, 28.7, 28.0, 24.3, 24.0, 21.5, 21.2, 17.1.

2.3 Synthesis of complex 2:

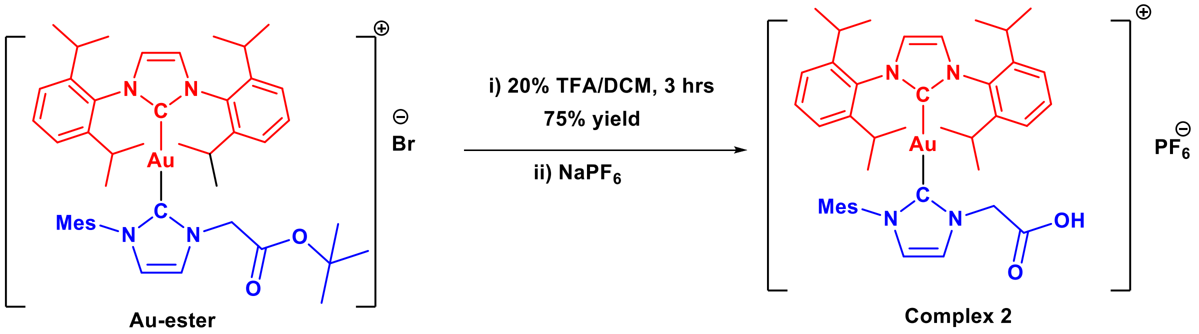

Au-ester (370 mg, 0.383 mmol) was transferred into a 20 ml scintillation vial and to that, 5 ml 20% TFA/DCM was added. The mixture was left to stir for 3 h. This was followed by the addition of 15 ml DCM. Next, the solution was subjected to an extraction with water, twice. The organic layer was then combined, dried over magnesium sulfate, and concentrated to 1-2 ml. Finally, hexanes were slowly added to the mixture. A white solid slowly precipitated out, which was collected via filtration.

For anion exchange, the filtered white solid was redissolved in 1 ml methanol and to that mixture, 10 equiv. of sodium hexafluorophosphate in 4 ml water was added. The mixture was stirred for 2 h and the precipitate was filtered to obtain complex **2** as white crystalline solid.

Yield: 270 mg, 73%.

ESI-HRMS (acetonitrile) (m/z): Calculated for $[C_{41}H_{52}AuN_4O_2]^+$: 829.3750. Observed : 829.3748.

^{1}H NMR (500 MHz, DMSO-*d*$_6$) δ 13.20 (s, 1H), 8.04 (s, 2H), 7.63 – 7.53 (m, 3H), 7.37 – 7.26 (m, 5H), 6.77 (s, 2H), 4.35 (s, 2H), 2.35 (q, *J* = 7.4, 6.9 Hz, 7H), 1.57 (s, 6H), 1.16 (d, *J* = 6.9 Hz, 12H), 1.05 (d, *J* = 6.9 Hz, 12H).

^{13}C NMR (126 MHz, DMSO-*d*$_6$) δ 186.2, 183.6, 168.5, 145.4, 138.9, 134.3, 134.2, 133.8, 131.0, 129.5, 125.4, 124.3, 124.2, 123.8, 50.9, 28.6, 24.23, 24.1, 21.2, 17.1.

2.4 General synthesis of products referred to as "Au-amides":

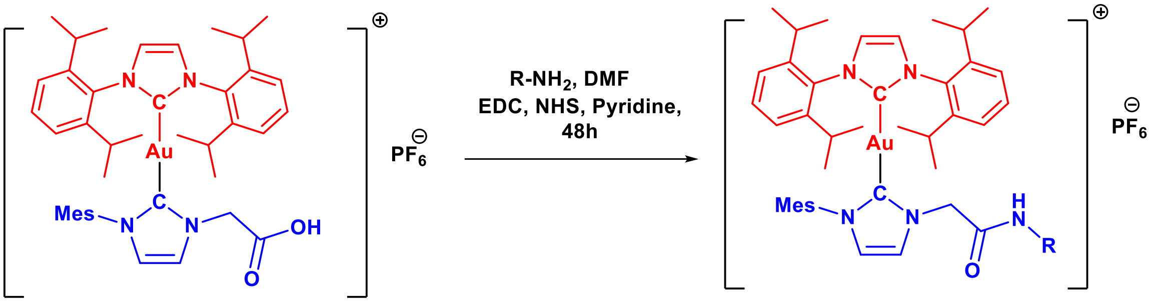

Complex 2 Au-amide

Complex **2** (1 equiv.), EDC (6 equiv.), and NHS (6 equiv.) were combined in an oven-dried round bottomed flask and left under vacuum for 15 min. After refilling the flask with nitrogen, 1.5 ml dry DMF was added to the mixture, which was left to stir for 1 h. Next, dry pyridine (2 equiv.) and the amine under study was added. The reaction was stirred for another 48 h. The reaction progress was monitored via LCMS. Once the reaction was deemed complete, the volatiles were evaporated off and to that 15 ml of DCM were added. The organic layer was extracted with water and collected after drying over magnesium sulfate. The concentrated organic layer containing the crude product was then purified by column chromatography over silica gel (eluent: Methanol/DCM: 5% to 10%) to afford the respective amide conjugate. Finally, the amide derivative was redissolved in methanol and anion exchange was performed as described above.

Entry no.	Coupling agent/Reaction cond.	Solvent	Temp (°C)	Time	Outcome
1.	CDI, TEA	ACN	RT/40	48 h	No product
2.	DCC, DMAP	ACN	RT/40	48 h	No product
3.	DCC,DMAP, Pentafluoro phenol (to get activated ester)	ACN	RT/40	48 h	No conversion
4.	Oxalyl chloride, Pyridine	DCM/DMF	RT	48 h	No product
5.	EDC, DMAP	DCM/ACN	40	48 h	Mixture of products
6.	EDC, NHS, Pyridine	DCM/ACN	40	48 h	Didn’t work
7.	PyBOP, DIPEA	DMF	RT	48 h	Product formed; hard to separate
8.	EDC, NHS, Pyridine	DMF	RT	48 h	Product formed; facile separation

Table S1. Reaction conditions screened to synthesize Au-Bis NHC amide conjugates (ethanol amine was used as the control amine).

2.4a Synthesis of complex 3:

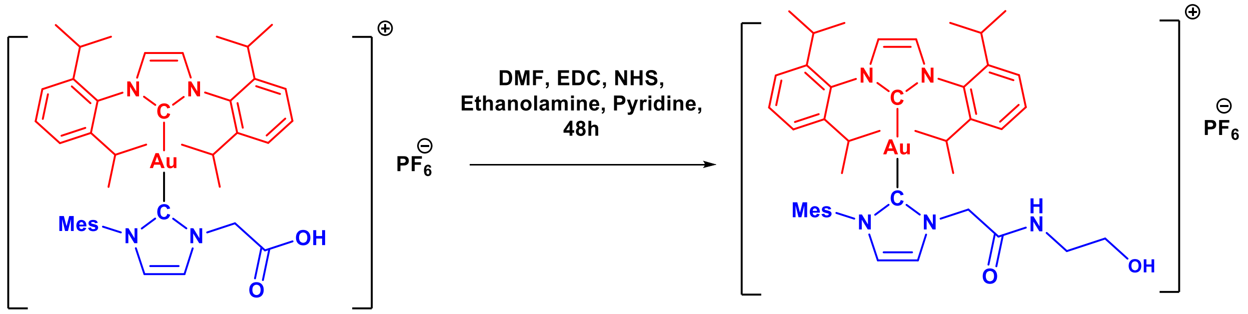

Following the general protocol described in section 2.4 above, complex **2** (35 mg, 0.036 mmol, 1 equiv.) was treated with ethanol amine (17 μl, mmol, 3 equiv.), EDC (34 mg, mmol, 6 equiv.), NHS (25 mg, 6 equiv.), and dry pyridine (5 μl, 2 equiv.) to afford complex **3** as a white crystalline solid. Dry DMF (1.5 ml) was used as the reaction solvent.

Yield: 20 mg, 55%.

ESI-HRMS (acetonitrile) (m/z): Calculated for $[C_{43}H_{57}AuN_5O_2]^+$: 872.4172. Observed: 872.4182.

^{1}H NMR (400 MHz, CD_2Cl_2) δ 7.57 (t, *J* = 7.8 Hz, 2H), 7.30 – 7.24 (m, 6H), 7.15 (d, *J* = 1.9 Hz, 1H), 6.76 (d, *J* = 1.9 Hz, 1H), 6.71 (s, 2H), 6.13 (s, 1H), 4.22 (s, 2H), 3.71 (t, *J* = 5.0 Hz, 2H), 3.37 (q, *J* = 5.3 Hz, 2H), 2.44 – 2.37 (m, 3H), 2.36 (d, *J* = 7.0 Hz, 4H), 1.66 (s, 6H), 1.18 (d, *J* = 6.9 Hz, 13H), 1.12 (d, *J* = 6.9 Hz, 12H).

^{13}C NMR (126 MHz, CD_2Cl_2) δ 187.1, 184.5, 165.4, 145.6, 139.0, 134.4, 133.9, 133.3, 130.8, 129.3, 124.1, 123.9, 122.9, 61.5, 52.0, 42.7, 28.7, 24.15, 23.7, 21.0, 16.9.

2.4b Synthesis of complex 4:

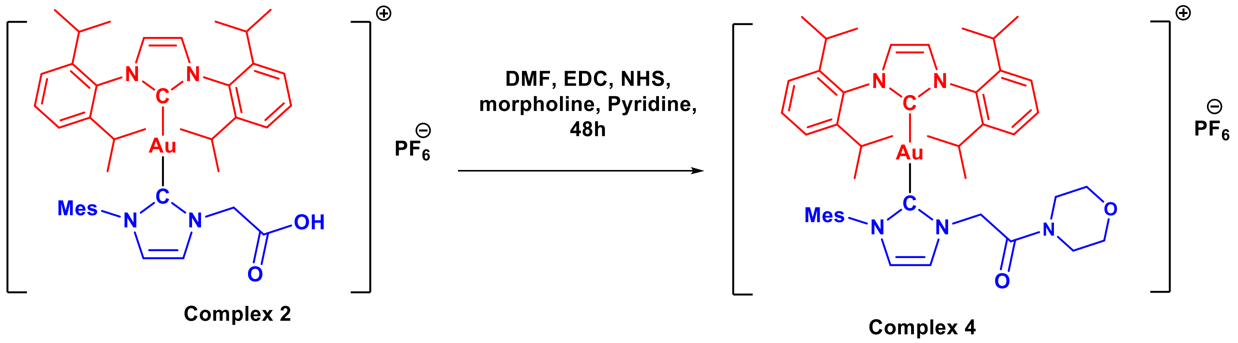

Following the general protocol described in section 2.4 above, complex **2** (50 mg, 0.051 mmol, 1 equiv.) was used along with morpholine (27 µl, 3 equiv.), EDC (49 mg, 6 equiv.), NHS (36 mg, 6 equiv.), and dry pyridine (7 µl, 2 equiv.) to afford complex **4** as a white crystalline solid. Dry DMF (1.5 ml) was used as the reaction solvent.

Yield: 24 mg, 45%.

ESI-HRMS (acetonitrile) (m/z): Calculated for $[C_{45}H_{59}AuN_5O_2]^+$:898.4329. Observed: 898.4323.

^{1}H NMR (400 MHz, CD_2Cl_2) δ 7.56 (t, *J* = 7.8 Hz, 2H), 7.30 – 7.22 (m, 6H), 7.15 – 7.11 (m, 1H), 6.77 – 6.72 (m, 3H), 4.50 (s, 2H), 3.70 (dt, *J* = 21.4, 4.8 Hz, 4H), 3.50 (t, *J* = 4.9 Hz, 2H), 3.25 (t, *J* = 4.8 Hz, 2H), 2.45 – 2.34 (m, 7H), 1.65 (s, 6H), 1.18 (d, *J* = 6.8 Hz, 12H), 1.09 (d, *J* = 6.9 Hz, 12H).

^{13}C NMR (126 MHz, CD_2Cl_2) δ 186.8, 184.4, 163.4, 145.6, 139.1, 134.4, 133.9, 133.4, 130.7, 129.4, 124.1, 124.0, 123.4, 122.8, 77.6, 77.3, 77.1, 66.5, 66.1, 50.9, 45.1, 42.5, 28.7, 24.3, 23.6, 20.9, 16.9.

2.4c Synthesis of complex 5:

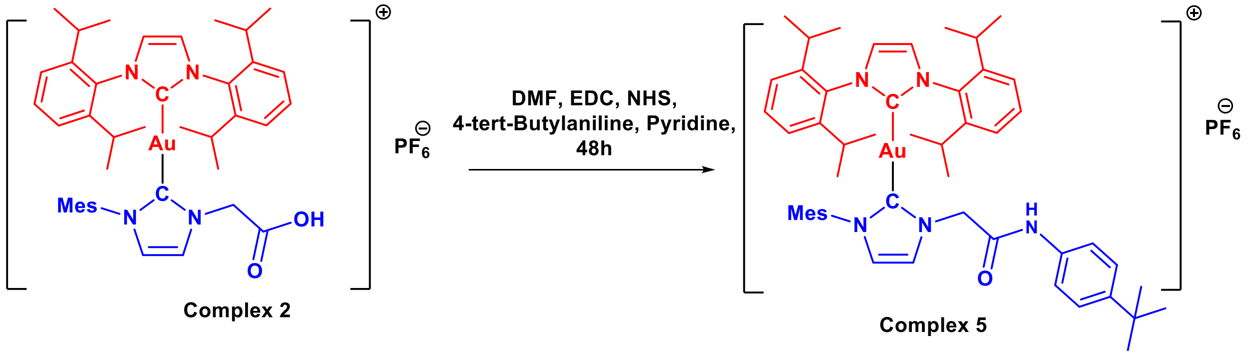

Following the general protocol described in section 2.4 above, complex **2** (35 mg, mmol, 1 equiv.) was used along with tert-butyl aniline (22 μl, 3 equiv.), EDC (34 mg, mmol, 6 equiv.), NHS (25 mg, 6 equiv.), and dry pyridine (5 μl, 2 equiv.) to afford complex **5** as a white crystalline solid. Dry DMF (1.5 ml) was used as the reaction solvent.

Yield: 11 mg, mmol, 28%.

ESI-HRMS (acetonitrile) (m/z): Calculated for $[C_{51}H_{65}AuN_5O]^+$: 960.4849. Observed: 960.4837.

^{1}H NMR (400 MHz, CD_2Cl_2) δ 7.72 (s, 1H), 7.55 – 7.50 (m, 2H), 7.47 – 7.38 (m, 4H), 7.23 (d, *J* = 0.9 Hz, 2H), 7.20 (dd, *J* = 7.8, 0.8 Hz, 4H), 7.08 (dd, *J* = 2.0, 0.9 Hz, 1H), 6.76 (dd, *J* = 1.9, 0.9 Hz, 1H), 6.73 (s, 2H), 4.40 (s, 2H), 2.44 – 2.28 (m, 7H), 1.70 (s, 6H), 1.34 (d, *J* = 0.8 Hz, 9H), 1.19 – 1.10 (m, 24H).

^{13}C NMR (126 MHz, CD_2Cl_2) δ 187.3, 184.9, 162.9, 147.3, 145.5, 138.9, 135.2, 134.4, 133.9, 133.3, 130.8, 129.2, 126.1, 125.9, 125.7, 124.0, 123.9, 122.8, 119.3, 52.6, 34.3, 31.3, 31.1, 30.9, 30.6, 28.7, 28.5, 24.1, 23.7, 21.0, 17.0.

2.4d Synthesis of complex 6:

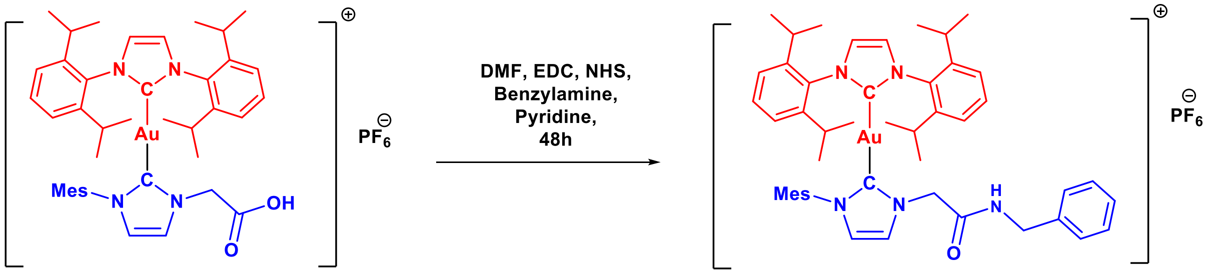

Following the general protocol described in section 2.4 above, complex **2** (35 mg, mmol, 1 equiv.) was used along with benzyl amine (μl, mmol, 3 equiv.), EDC (34 mg, mmol, 6 equiv.), NHS (25 mg, 6 equiv.), and dry pyridine (5 μl, 2 equiv.) to afford complex **6** as a white crystalline solid. Dry DMF (1.5 ml) was used as the reaction solvent.

Yield: 15 mg, mmol, 39%.

ESI-HRMS (acetonitrile) (m/z): Calculated for $[C_{48}H_{59}AuN_5O]^+$: 918.4380. Observed: 918.4372.

^{1}H NMR (500 MHz, CD_2Cl_2) δ 7.38 (t, *J* = 7.8 Hz, 2H), 7.33 (t, *J* = 7.5 Hz, 2H), 7.27 (d, *J* = 6.4 Hz, 3H), 7.16 (s, 2H), 7.14 – 7.07 (m, 5H), 6.69 (d, *J* = 1.9 Hz, 1H), 6.63 (s, 2H), 5.99 (t, *J* = 6.0 Hz, 1H), 4.31 (d, *J* = 5.9 Hz, 2H), 4.12 (s, 2H), 2.28 (q, *J* = 7.0 Hz, 7H), 1.59 (s, 6H), 1.08 (d, *J* = 6.8 Hz, 12H), 1.01 (d, *J* = 6.9 Hz, 12H).

^{13}C NMR (126 MHz, CD_2Cl_2) δ 187.0, 184.2, 164.5, 145.6, 139.1, 137.9, 134.3, 133.8, 133.3, 130.7, 129.3, 128.7, 128.2, 127.7, 124.0, 123.0, 122.8, 52.2, 43.8, 28.7, 24.2, 23.6, 21.0, 17.0.

2.4e Synthesis of complex 7:

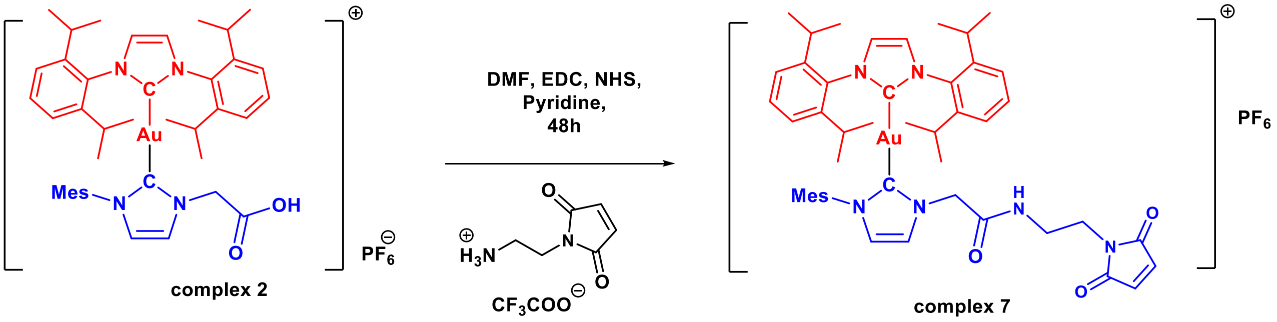

Following the general protocol described in section 2.4 above, complex **2** (42.5 mg, mmol, 1 equiv.) was used along with N-(2-aminoethyl) maleimide (32.5 mg, mmol, 3 equiv.), EDC (40 mg, mmol, 6 equiv.), NHS (28 mg, 6 equiv.), and dry pyridine (6 μl, 2 equiv.) to afford complex **7** as a white crystalline solid. Dry DMF (1.5 ml) was used as the reaction solvent.

Yield: 21 mg, mmol, 44%.

ESI-HRMS (acetonitrile) (m/z): Calculated for $[C_{47}H_{58}AuN_6O_3]^+$: 951.4236. Observed: 951.4219.

^{1}H NMR (600 MHz, CD_2Cl_2) δ 7.57 (t, *J* = 7.8 Hz, 2H), 7.27 (dd, *J* = 7.6, 1.1 Hz, 6H), 7.24 – 7.22 (m, 1H), 6.80 – 6.77 (m, 1H), 6.75 – 6.70 (m, 4H), 6.25 – 6.15 (m, 1H), 4.14 (s, 2H), 3.69 (dd, *J* = 6.3, 4.7 Hz, 2H), 3.38 (q, *J* = 5.7 Hz, 2H), 2.45 – 2.34 (m, 7H), 1.19 (dd, *J* = 6.9, 1.1 Hz, 13H), 1.12 (dd, *J* = 6.9, 1.0 Hz, 12H).

^{13}C NMR (151 MHz, CD_2Cl_2) δ 187.2, 184.5, 171.1, 165.3, 145.6, 138.9, 134.4, 134.1, 133.9, 133.3, 130.8, 129.2, 124.0, 123.8, 122.9, 122.8, 52.1, 38.9, 36.7, 29.7, 28.7, 24.1, 23.7, 20.9, 16.9.

2.4f Synthesis of complex 8:

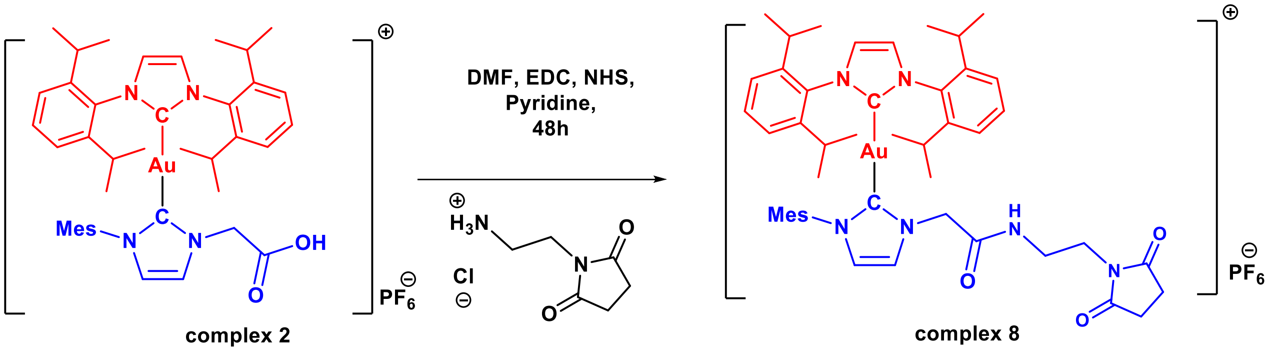

Following the general protocol described in section 2.4 above, complex **2** (35 mg, mmol, 1 equiv.) was used along with 1-(2-Amino-ethyl)-pyrrole-2,5-dione hydrochloride (30 mg, 3 equiv.), EDC (34 mg, mmol, 6 equiv.), NHS (25 mg, 6 equiv.), dry pyridine (5 µl, 2 equiv.) to afford complex **8** as a white crystalline solid. Dry DMF (1.5 ml) was used as the reaction solvent.

Yield: 23 mg, mmol, 58%.

ESI-HRMS (acetonitrile) (m/z): Calculated for $[C_{47}H_{60}AuN_6O_3]^+$:953.4387. Observed: 953.4386.

^{1}H NMR (400 MHz, CD_2Cl_2) δ 7.53 (dd, *J* = 9.0, 6.2 Hz, 2H), 7.30 – 7.18 (m, 7H), 6.74 (d, *J* = 5.2 Hz, 1H), 6.70 (d, *J* = 6.2 Hz, 2H), 6.34 (d, *J* = 6.0 Hz, 1H), 4.08 (d, *J* = 6.6 Hz, 2H), 3.65 (q, *J* = 7.7, 6.4 Hz, 2H), 3.35 (d, *J* = 6.9 Hz, 2H), 2.73 – 2.61 (m, 4H), 2.36 (dd, *J* = 7.3, 4.7 Hz, 7H), 1.66 (d, *J* = 7.2 Hz, 6H), 1.17 (t, *J* = 6.9 Hz, 12H), 1.10 (t, *J* = 6.9 Hz, 12H).

^{13}C NMR (126 MHz, CD_2Cl_2) δ 187.4, 184.5, 178.3, 165.6, 145.6, 138.9, 134.5, 133.9, 133.3, 130.9, 129.2, 124.0, 123.8, 123.2, 122.7, 52.0, 38.2, 37.5, 28.7, 28.2, 24.1, 23.7, 21.0, 16.9.

2.4g Synthesis of complex 9:

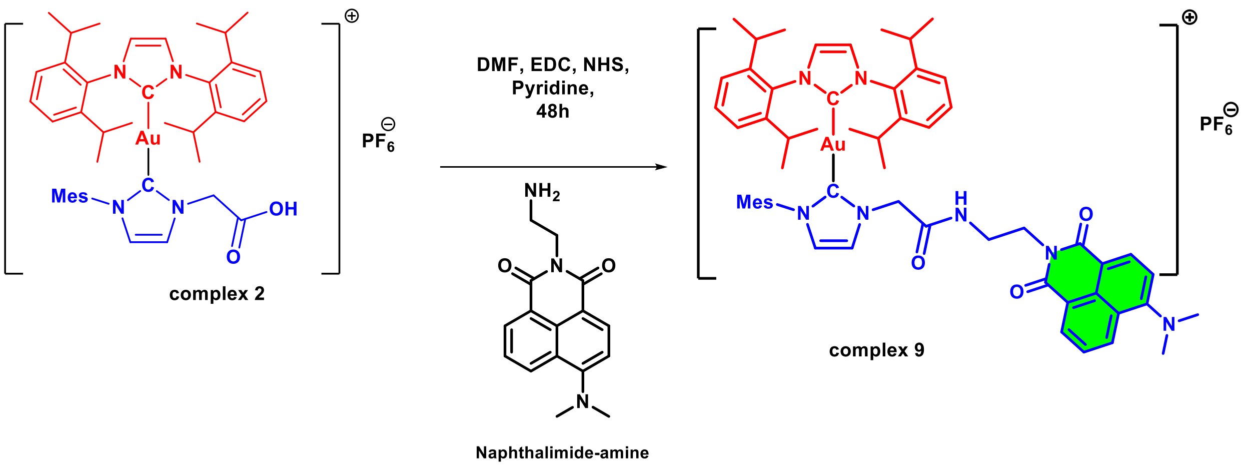

Following the general protocol described in section 2.4 above, complex **2** (40 mg, 0.041 mmol, 1 equiv.) was used along with naphthalimide amine (37 mg, 3 equiv.) EDC (39 mg, mmol, 6 equiv.), NHS (27 mg, 6 equiv.), dry pyridine (6 μl, 2 equiv.) to afford complex **9** as a yellow crystalline solid. Dry DMF (1.5 ml) was used as the reaction solvent.

Yield: 19 mg, mmol, 37%.

ESI-HRMS (acetonitrile) (m/z): Calculated for $[C_{57}H_{67}AuN_7O_3]^+$:1094.4965. Observed: 1096.4973.

^{1}H NMR (400 MHz, CD_2Cl_2) δ 8.57 (dd, *J* = 7.3, 1.2 Hz, 1H), 8.53 – 8.41 (m, 2H), 7.70 (dd, *J* = 8.5, 7.3 Hz, 1H), 7.53 (t, *J* = 7.8 Hz, 2H), 7.26 – 7.19 (m, 6H), 7.14 (d, *J* = 8.3 Hz, 1H), 7.08 (d, *J* = 1.8 Hz, 1H), 6.76 (d, *J* = 1.8 Hz, 1H), 6.72 (s, 2H), 6.18 (s, 1H), 4.37 (t, *J* = 5.6 Hz, 2H), 4.13 (s, 2H), 3.56 (q, *J* = 5.4 Hz, 2H), 3.14 (d, *J* = 0.8 Hz, 6H), 2.42 – 2.30 (m, 7H), 1.64 (s, 6H), 1.14 (d, *J* = 6.9 Hz, 12H), 1.07 (d, *J* = 6.9 Hz, 12H).

^{13}C NMR (126 MHz, CD_2Cl_2) δ 187.6, 184.2, 165.6, 164.8, 164.2, 157.1, 145.5, 138.8, 134.5, 134.1, 133.3, 132.5, 131.3, 130.9, 130.4, 129.1, 125.2, 124.7, 124.0, 123.8, 123.1, 122.4, 114.7, 113.1, 52.5, 44.6, 39.1, 38.2, 29.7, 28.7, 24.1, 23.7, 20.9, 17.0.

3. Results from Analytical Experiments:

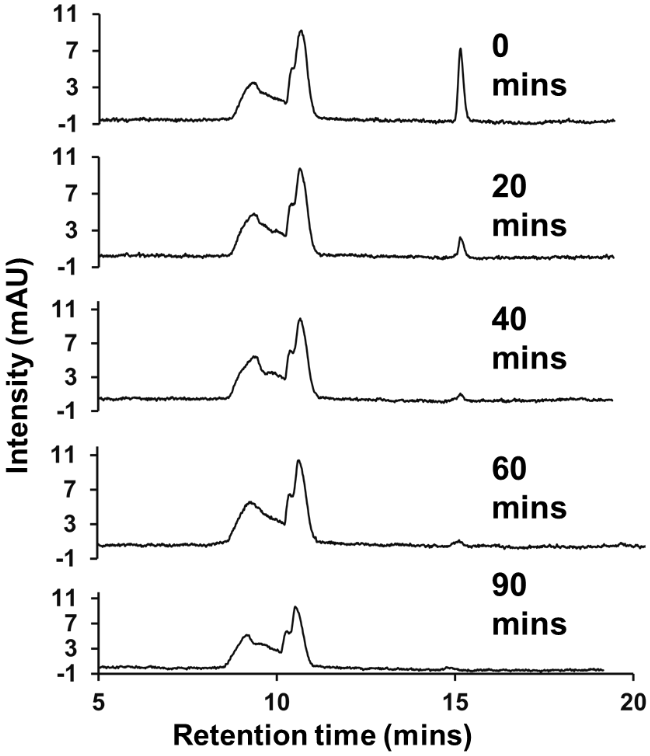

Figure S1. Time-dependent HPLC studies on HSA (600 μM) binding with complex **7** (200 μM).

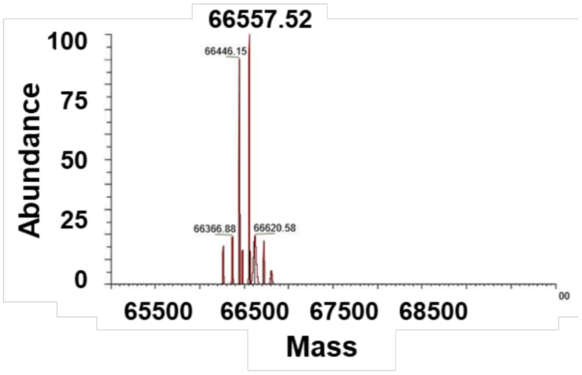

Figure S2. Deconvolved mass-spectrum of HSA (60 μM).

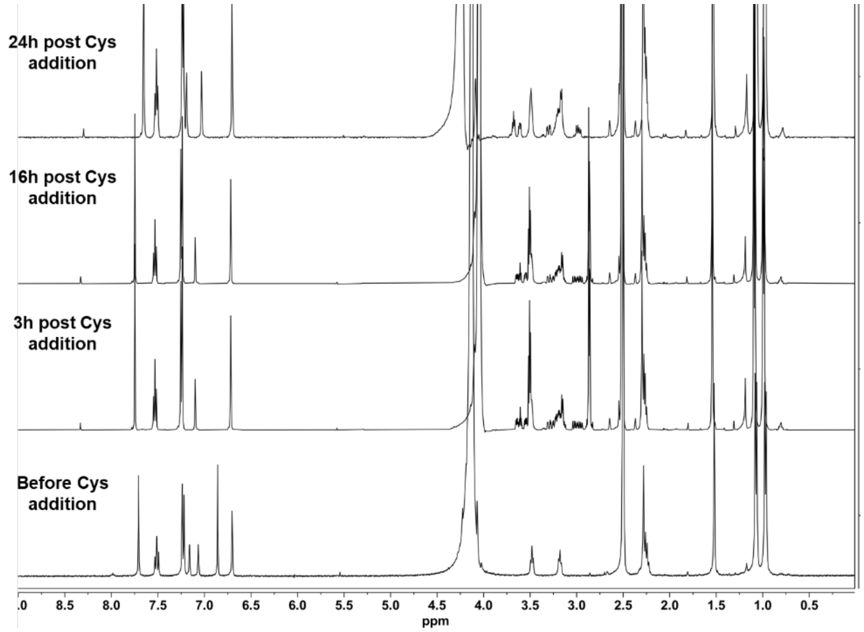

Figure S3: Time dependent ^{1}H NMR spectrum of the solution containing complex **7** (0.6 mM) and cysteine (5 equiv.) in DMSO-d_6/D_2O (3:2) mixture. Solutions were kept under nitrogen and occasionally bubbled to avoid cysteine oxidation.

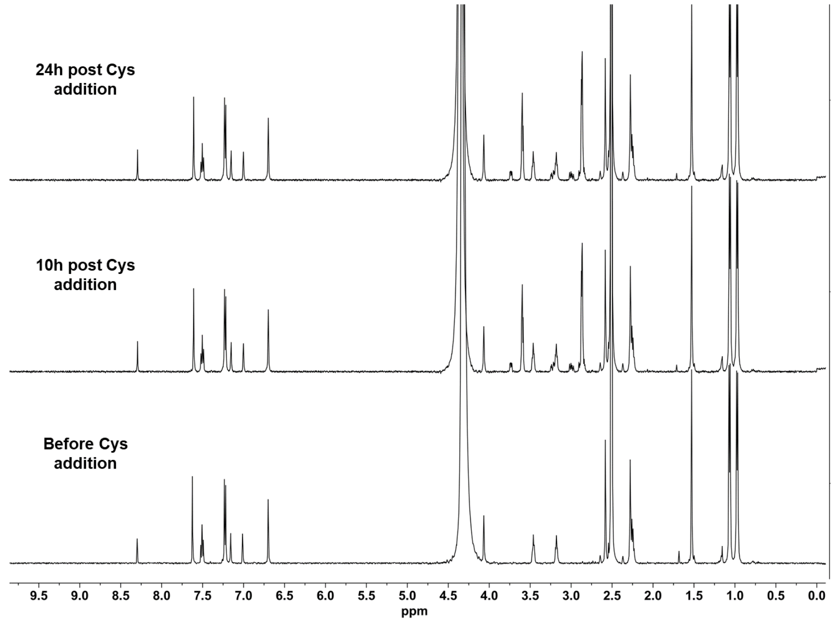

Figure S4: Time dependent ^{1}H NMR spectrum of the solution containing complex **8** (0.6 mM) and cysteine (5 equiv.) in DMSO-d_6/D_2O (3:2) mixture. Solutions were kept under nitrogen and continuously bubbled to avoid cysteine oxidation.

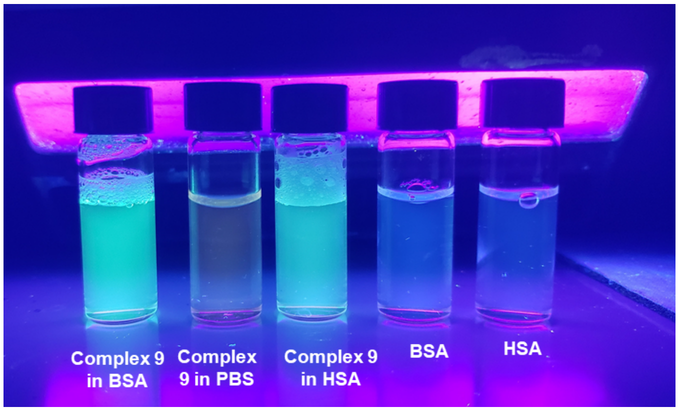

Figure S5. Increase in fluorescence intensity of complex **9** (2.5 μM) in the presence of BSA (30 μM) or HSA (30 μM) compared to PBS only.

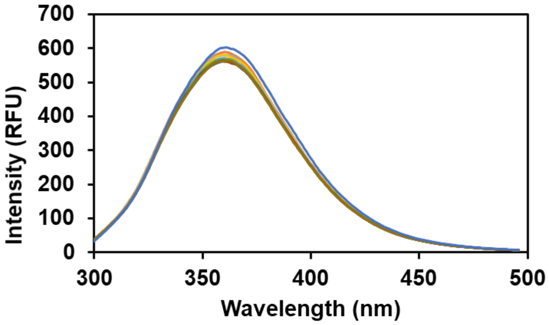

Figure S6. Fluorescence spectra of HSA (3 μM) recorded in the presence of increasing quantities of complex **2** (0-3 equiv.).

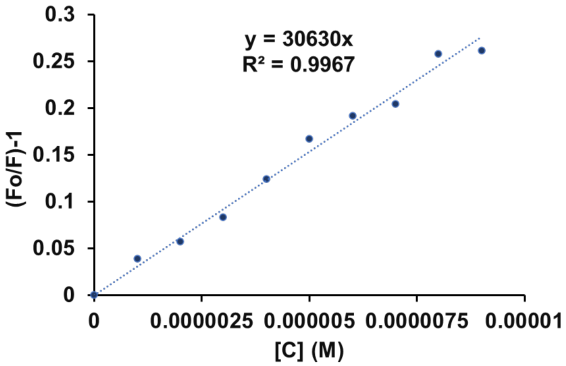

Figure S7. Stern-Volmer plot for the fluorescence quenching of HSA observed upon treatment with complex **9**.

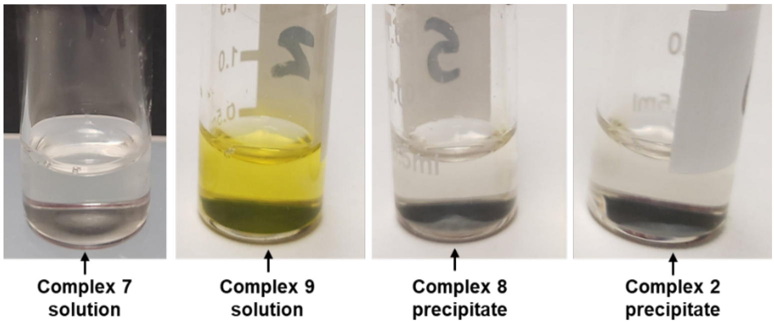

Figure S8. Photographs of 150 μM solutions of complex **7**, complex **9**, and complex **8,** and complex **2** in HSA (600 μM) after 12 h and subjecting to centrifugation (final DMSO concentration 2%). Complexes **8** and **2** precipitate out, while complexes **7** and **9** remain in solution (even after 96 h).

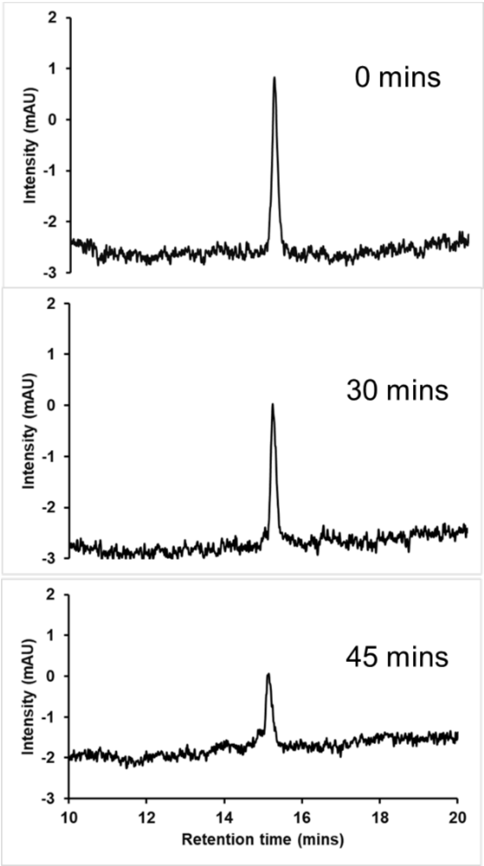

Figure S9. HPLC Chromatogram of complex **7** (100 μM) incubated with RPMI-1640 media mixed with 10% fetal bovine serum. The gradual decrease in the peak area was taken as evidence of covalent binding between the serum protein and complex **7**.

4. Results from Biological Experiments

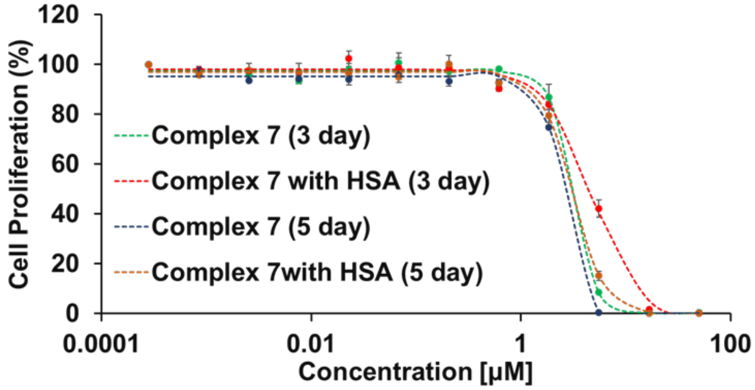

Compound	IC_{50} (μM)	Std. Dev.
Complex **7** (3day)	3.102	0.158
Complex **7** with HSA (3 day)	4.842	0.368
Complex **7** (5 day)	2.309	0.423
Complex **7** with HSA (5 day)	3.110	0.144

Figure S10. Left) 5 day cell proliferation profile for complex **7** in A549 cells. Right) IC_{50} values obtained from the MTT assay.

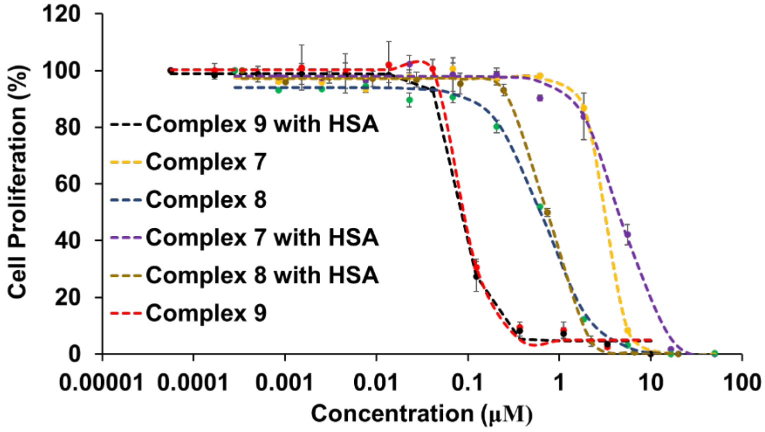

Figure S11. Cell proliferation profile obtained from 3 day MTT assay against A549 lung cancer cells of various amide conjugates pre- and post-incubation with HSA.

5. Crystallographic Analyses

Code name	Complex **2**	Complex **3**	Complex **4**	Complex **7**
**Temperature**	100(2) K	100(2) K	100(2) K	100(2) K
**Wavelength**	0.71073 Å	1.54184 Å	1.54184 Å	1.54184 Å
**Crystal system**	triclinic	triclinic	monoclinic	triclinic
**Space group**	P -1	P -1	P 1 21 1	P -1
**a**	13.630(2) Å	11.0894(3) Å	11.3001(3) Å	11.0621(2) Å
**b**	15.952(2) Å	18.0488(4) Å	50.4909(13) Å	18.0164(3) Å
**c**	19.288(3) Å	24.6597(5) Å	17.6766(4) Å	25.9531(5) Å
**α**	86.368(4)°	101.6313(18)°	90°	102.579(2)°
**β**	88.416(4)°	93.6495(19)°	100.564(2)°	92.7990(10)°
**γ**	78.854(3)°	101.640(2)°	90°	98.3590(10)°
**Volume**	4105.8(10) $Å^3$	4706.79(19) $Å^3$	9914.5(4) $Å^3$	4977.26(16) $Å^3$
**Z**	2	4	8	2
**Density (calculated)**	1.405 g/cm^3	1.436 g/cm^3	1.399 g/cm^3	1.521 g/cm^3
**Absorption coefficient**	4.106 mm^{-1}	6.726 mm^{-1}	6.401 mm^{-1}	6.933 mm^{-1}
**F(000)**	1750	2056	4224	2300
**Crystal size**	0.360 x 0.120 x 0.090 mm^3	0.21 x 0.1 x 0.067 mm^3	0.093 x 0.01 x 0.01 mm^3	0.231 x 0.08 x 0.046 mm^3
**Theta range for data collection**	3.007 to 27.493°	2.808 to 73.445°	2.543 to 67.073°	2.742 to 73.389°.
**Index ranges**	-17<=h<=17, -20<=k<=20, -25<=l<=25	-12<=h<=13, -22<=k<=15, -30<=l<=30	-12<=h<=13, -50<=k<=60, -21<=l<=21	-13<=h<=13, -20<=k<=22, -32<=l<=32
**Reflections collected**	65764	32664	56285	66015
**Independent reflections**	18451 [R(int) = 0.0391]	17996 [R(int) = 0.0475]	28213 [R(int) = 0.0781]	19449 [R(int) = 0.0350]
**Completeness to theta**	99.0 % (theta = 25.242°)	98.4 % (theta = 67.684°)	98.9 % (theta = 67.073°)	99.5 % (theta = 67.684°)
**Absorption correction**	Semi-empirical from equivalents	Gaussian and multi-scan	Gaussian and multi-scan	Gaussian and multi-scan
**Max. and min. transmission**	1.00 and 0.607	1.00 and 0.508	1.000 and 0.729	0.952 and 0.307
**Refinement method**	Full-matrix least-squares on F^2	Full-matrix least-squares on F^2	Full-matrix least-squares on F^2	Full-matrix least-squares on F^2
**Data / restraints / parameters**	18451 / 1188 / 1062	17996 / 350 / 1208	28213 / 4029 / 2345	19449 / 1779 / 1467
**Goodness-of-fit on F2**	1.031	1.025	1.032	1.047
**Final R indices [I>2σ(I)]**	R1 = 0.0387, wR2 = 0.1014	R1 = 0.0470, wR2 = 0.1219	R1 = 0.0615, wR2 = 0.1375	R1 = 0.0364, wR2 = 0.0891
**R indices (all data)**	R1 = 0.0439, wR2 = 0.1048	R1 = 0.0555, wR2 = 0.1304	R1 = 0.0922, wR2 = 0.1499	R1 = 0.0425, wR2 = 0.0940
**Largest diff. peak and hole**	3.577 and -3.793 e.$Å^{-3}$	1.953 and -1.452 e.$Å^{-3}$	1.643 and -1.715 e.$Å^{-3}$	1.105 and -1.538 e.$Å^{-3}$
**CCDC Number**	2062337	2062338	2062340	2062339

Table S2: Structural details for complexes **2, 3**, **4**, and **7** from X-ray diffraction studies.

Experimental details for complex 2:

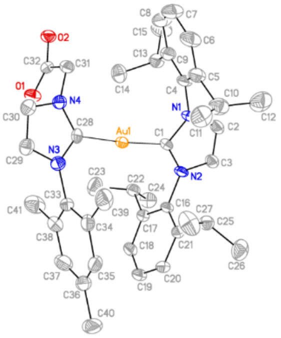

X-ray Experimental for complex **2** (bromide salt): Crystals grew as clusters of white prisms by slow diffusion of diethyl ether in dichloromethane solution. The data crystal was cut from a larger crystal and had approximate dimensions; 0.36 x 0.12 x 0.09 mm. The data were collected on a Rigaku AFC12 diffractometer with a Saturn 724+ CCD using a graphite monochromator with Mo K_α radiation (λ = 0.71073 Å). A total of 1376 frames of data were collected using ω-scans with a scan range of 0.5° and a counting time of 20 seconds per frame. The data were collected at 100 K using an Rigaku XStream Cryostream low temperature device. Details of crystal data, data collection and structure refinement are listed in Table S2. Data reduction were performed using the Rigaku Americas Corporation's Crystal Clear version 1.40.[9] The structure was solved by direct methods using SUPERFLIP[10] and refined by full-matrix least-squares on F^2 with anisotropic displacement parameters for the non-H atoms using SHELXL-2016/6.[11] Structure analysis was aided by use of the programs PLATON[12] and WinGX.[13] The hydrogen atoms on carbon were calculated in ideal positions with isotropic displacement parameters set to 1.2xU_{eq} of the attached atom (1.5xU_{eq} for methyl hydrogen atoms).

The Br ion, an isopropyl group on complex **2** with Au1 and the 2,6-di-isopropyl benzene group were disordered. The disorder was modeled in essentially the same way for each component. For example, for the 2,6-diisopropyl benzene group, the site occupancy factors for the non-H atoms were set to equal the variable x. The site occupancy factors for the alternate component were set to (1-x). A common isotropic displacement parameter was refined for all atoms involved in the disorder while refining x. The geometry of the two components was restrained to be equivalent throughout the refinement process. Upon convergence of the variable x, the site occupancy factors were fixed for the remainder of the refinement. Displacement parameters for the non-H atoms of the disordered groups were restrained to be equivalent in the final refinement model. The displacement parameters for the disordered Br ions were constrained to be equal in the final refinement model.

The function, $\Sigma w(|F_o|^2 - |F_c|^2)^2$, was minimized, where $w = 1/[(\sigma(F_o))^2 + (0.0494*P)^2 + (16.8991*P)]$ and $P = (|F_o|^2 + 2|F_c|^2)/3$. $R_w(F^2)$ refined to 0.105, with R(F) equal to 0.0387 and a goodness of fit, S, = 1.03. Definitions used for calculating R(F), $R_w(F^2)$ and the goodness of fit, S, are given below.[14] The data were checked for secondary extinction effects, but no correction was necessary. Neutral atom scattering factors and values used to calculate the linear absorption coefficient are from the International Tables for X-ray Crystallography (1992).[15] All figures were generated using SHELXTL/PC.[16] For details related to bond lengths and bond angles, please check CIF files corresponding to CCDC #2062337.

Experimental details for complex 3:

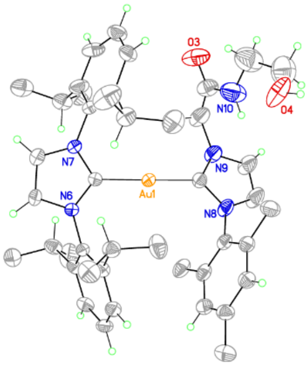

X-ray Experimental for complex **3**: Crystals grew as clusters of colorless needles by slow evaporation from dichloromethane and dichloroethane. The data crystal was separated from a cluster of crystals and had approximate dimensions; 0.21 x 0.10 x 0.067 mm. The data were collected on an Agilent Technologies SuperNova Dual Source diffractometer using a μ-focus Cu Kα radiation source (λ = 1.5418 Å) with collimating mirror monochromators. A total of 2446 frames of data were collected using ω-scans with a scan range of 0.5° and a counting time of 11 seconds per frame for frames collected with a detector offset of +/- 41.6° and 41 seconds per frame with frames collected with a detector offset of 107.1°. The data were collected at 100 K using an Oxford Cryostream low temperature device. Details of crystal data, data collection and structure refinement are listed in Table S2. Data collection, unit cell refinement and data reduction were performed using Rigaku Oxford Diffraction's CrysAlisPro V 1.171.40.53.[17] The structure was solved by direct methods using SHELXT[18] and refined by full-matrix least-squares on F^2 with anisotropic displacement parameters for the non-H atoms using SHELXL-2016/6.[11] Structure analysis was aided by use of the programs PLATON[12] and OLEX2.[19] The hydrogen atoms on the carbon atoms were calculated in ideal positions with isotropic displacement parameters set to 1.2xUeq of the attached atom (1.5xUeq for methyl hydrogen atoms).

A molecule of DCM was disordered. The contributions to the scattering factors due to this solvent molecule were removed by use of the utility SQUEEZE[20] in PLATON. PLATON was used as incorporated in OLEX2. In addition, a hexafluoro phosphate anion and a hydroxyethylamide group on one of the Au complexes was disordered. The disorder was modeled using features available in OLEX2.

The function, $\Sigma w(|F_O|^2 - |F_C|^2)^2$, was minimized, where $w = 1/[(\sigma(F_O))^2 + (0.0748*P)^2 + (1.822*P)]$ and $P = (|F_O|^2 + 2|F_C|^2)/3$. $R_W(F^2)$ refined to 0.130, with R(F) equal to 0.0470 and a goodness of fit, S, = 1.06. Definitions used for calculating R(F), $R_W(F^2)$ and the goodness of fit, S, are given below.[14] The data were checked for secondary extinction effects but no correction was necessary. Neutral atom scattering factors and values used to calculate the linear absorption coefficient are from the International Tables for X-ray Crystallography (1992).[15] All figures were generated using SHELXTL/PC.[16] For details related to bond lengths and bond angles, please check CIF files corresponding to CCDC #2062338.

Experimental details for complex 4:

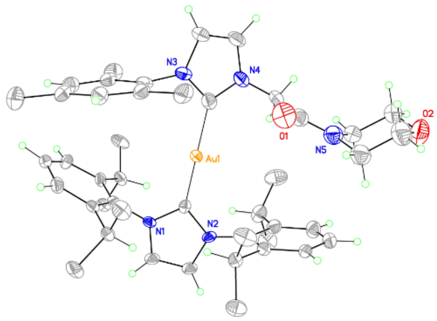

X-ray Experimental for complex **4**: Crystals grew as very, small thin needles by vapor diffusion of diethyl ether into a dichloromethane solution of the complex. The data crystal had approximate dimensions; 0.09 x 0.01 x 0.01 mm. The data were collected on a Rigaku Oxford Diffraction Synergy S Dual Source diffractometer with a HyPix 6000E detector using a μ-focus Cu Kα radiation source (λ = 1.5418 Å) with collimating mirror monochromators. A total of 1697 frames of data were collected using ω-scans with a scan range of 0.5° and a counting time of 8 seconds per frame for frames collected with a detector offset of +/- 47.9° and 32 seconds per frame with frames collected with a detector offsets of -91.8 and 107.1°. The data were collected at 100 K using an Oxford Cryostream low temperature device. Details of crystal data, data collection and structure refinement are listed in Table S2. Data collection, unit cell refinement and data reduction were performed using Rigaku Oxford Diffraction's CrysAlisPro V 1.171.41.70.[17] The structure was solved by direct methods using SHELXT[18] and refined by full-matrix least-squares on F^2 with anisotropic displacement parameters for the non-H atoms using SHELXL-2016/6.[11] Structure analysis was aided by use of the programs PLATON[12] and OLEX2.[19] The hydrogen atoms on the carbon atoms were calculated in ideal positions with isotropic displacement parameters set to 1.2xUeq of the attached atom (1.5xUeq for methyl hydrogen atoms).

One of the hexafluorophosphate anions and a morpholine group were disordered. The disorder was modeled using features found in OLEX2. A large region of disordered solvent was observed in the unit cell. The solvent could not be satisfactorily modeled. The contributions to the scattering factors due to these solvent molecules were removed by using SQUEEZE.[20]

The function, $\Sigma w(|F_o|^2 - |F_c|^2)^2$, was minimized, where $w = 1/[(\sigma(F_o))^2 + (0.0605*P)^2 + (14.321*P)]$ and $P = (|F_o|^2 + 2|F_c|^2)/3$. $R_w(F^2)$ refined to 0.150, with R(F) equal to 0.0615 and a goodness of fit, S, = 0.980. Definitions used for calculating R(F), $R_w(F^2)$ and the goodness of fit, S, are given below.[14] The data were checked for secondary extinction effects, but no correction was necessary. Neutral atom scattering factors and values used to calculate the linear absorption coefficient are from the International Tables for X-ray Crystallography (1992).[15] All figures were generated using SHELXTL/PC.[16] For details related to bond lengths and bond angles, please check CIF files corresponding to CCDC #2062340.

Experimental details for complex 7:

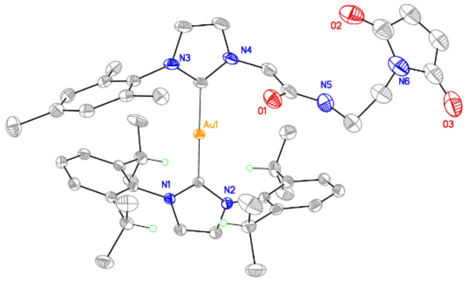

X-ray Experimental for complex **7**: Crystals grew as colorless laths by vapor diffusion of diethyl ether into a dichloromethane solution of the Au complex. The data crystal was separated from a cluster of crystals and had approximate dimensions; 0.23 x 0.080 x 0.046 mm. The data were collected on an Agilent Technologies SuperNova Dual Source diffractometer using a μ-focus Cu Kα radiation source (λ = 1.5418 Å) with collimating mirror monochromators. A total of 2186 frames of data were collected using ω-scans with a scan range of 1° and a counting time of 4 seconds per frame for frames collected with a detector offset of +/- 41.6° and 16 seconds per frame with frames collected with a detector offset of 107.1°. The data were collected at 100 K using an Oxford Cryostream low temperature device. Details of crystal data, data collection and structure refinement are listed in Table S2. Data collection, unit cell refinement and data reduction were performed using Rigaku Oxford Diffraction's CrysAlisPro V 1.171.41.70.[17] The structure was solved by direct methods using SHELXT[18] and refined by full-matrix least-squares on F^2 with anisotropic displacement parameters for the non-H atoms using SHELXL-2016/6.[11] Structure analysis was aided by use of the programs PLATON[12] and OLEX2.[19] The hydrogen atoms were calculated in ideal positions with isotropic displacement parameters set to 1.2xUeq of the attached atom (1.5xUeq for methyl hydrogen atoms). The structure consists of two Au hexafluorophosphate complexes with a molecule of dichloromethane. One of the hexafluorophosphate ions is disordered. In addition, both of the maleimide portions of the Au complexes are also disordered. The disorder was modeled using features found in OLEX2.

The function, $\Sigma w(|F_o|^2 - |F_c|^2)^2$, was minimized, where $w = 1/[(\sigma(F_o))^2 + (0.0363*P)^2 + (14.974*P)]$ and $P = (|F_o|^2 + 2|F_c|^2)/3$. $R_w(F^2)$ refined to 0.0940, with R(F) equal to 0.0364 and a goodness of fit, S, = 1.03. Definitions used for calculating R(F), $R_w(F^2)$ and the goodness of fit, S, are given below.[14] The data were checked for secondary extinction effects but no correction was necessary. Neutral atom scattering factors and values used to calculate the linear absorption coefficient are from the International Tables for X-ray Crystallography (1992).[15] All figures were generated using SHELXTL/PC.[16] For details related to bond lengths and bond angles, please check CIF files corresponding to CCDC #2062339.

6. NMR spectra:

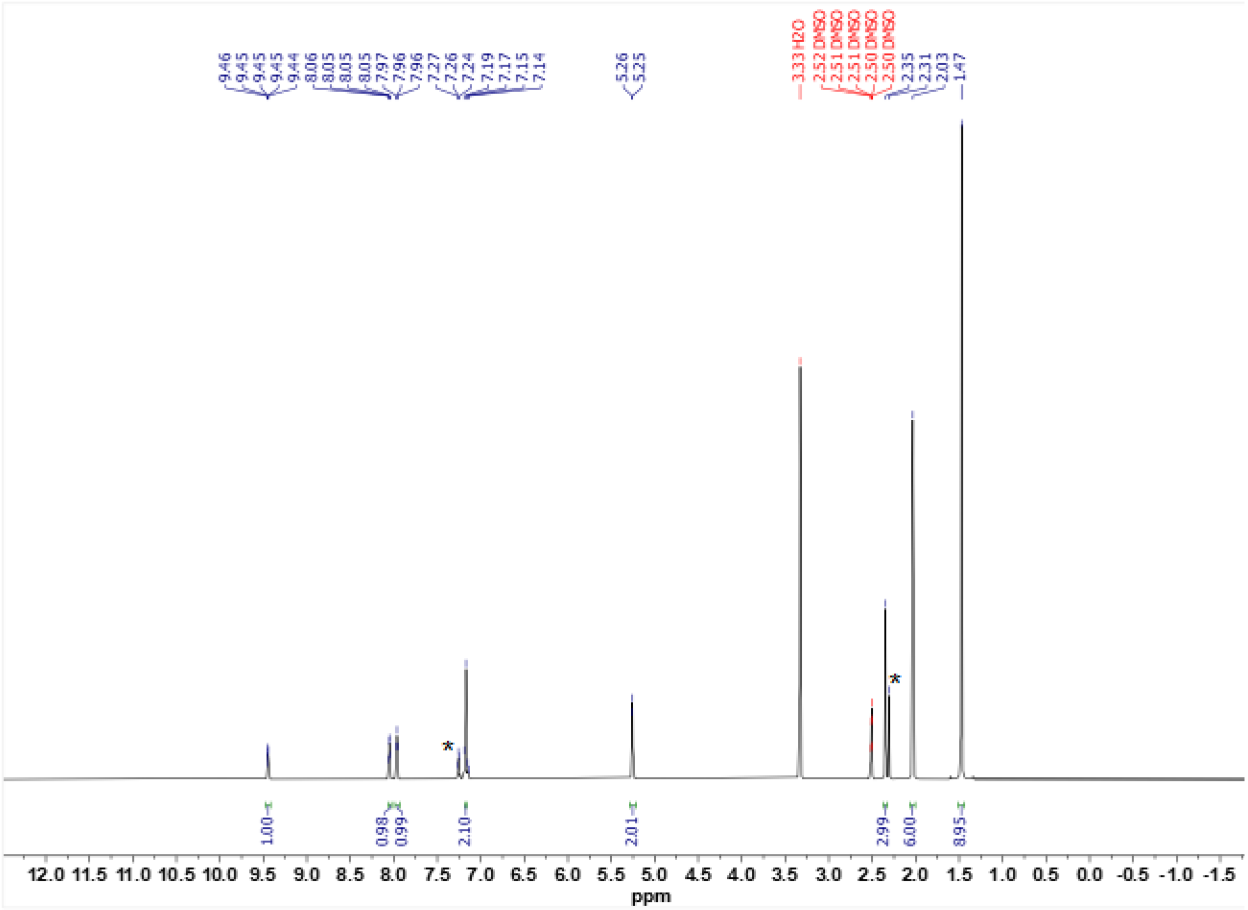

^{1}H NMR spectrum of **Imid ester** (* denotes toluene peaks) [Solvent: DMSO-d_6]

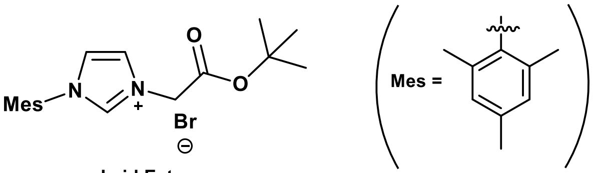

Imid Ester

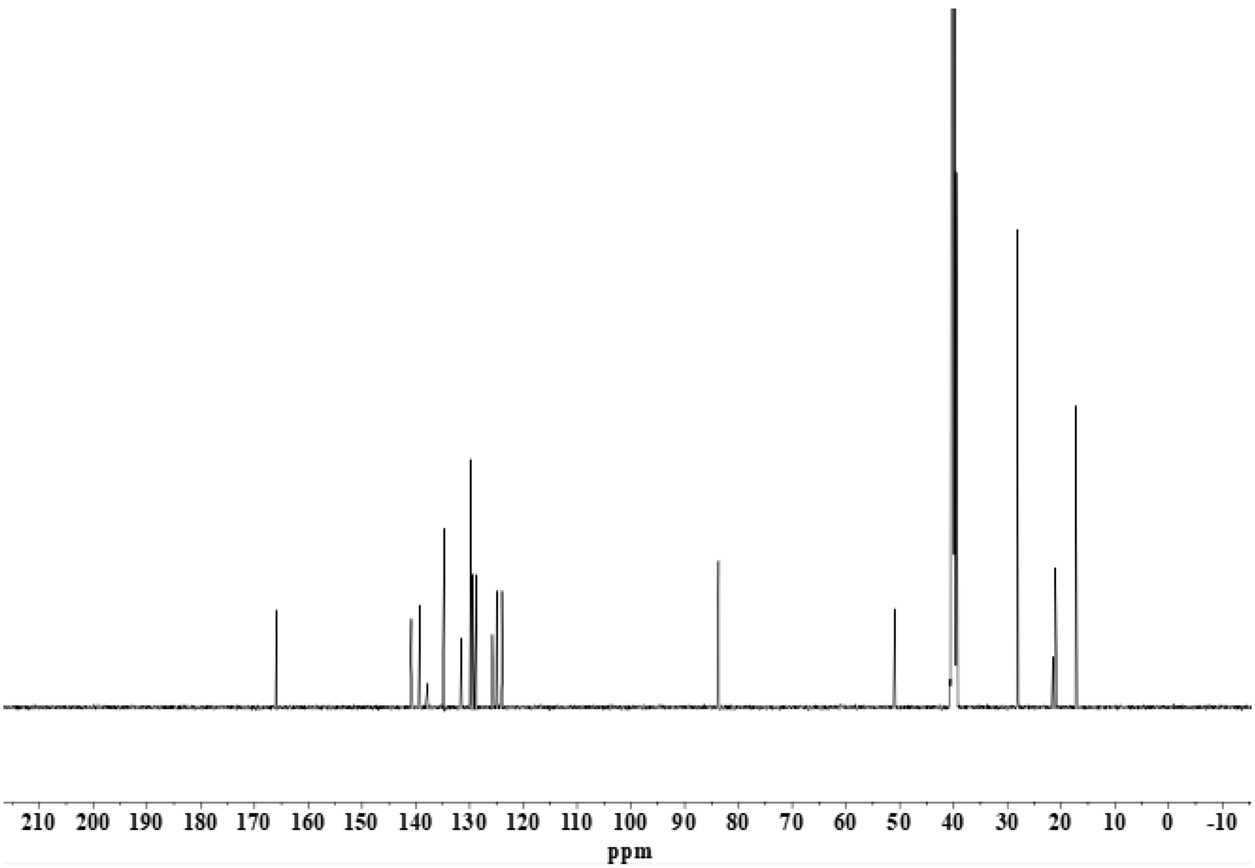

^{13}C NMR spectrum of **Imid ester** [Solvent: DMSO-d_6]

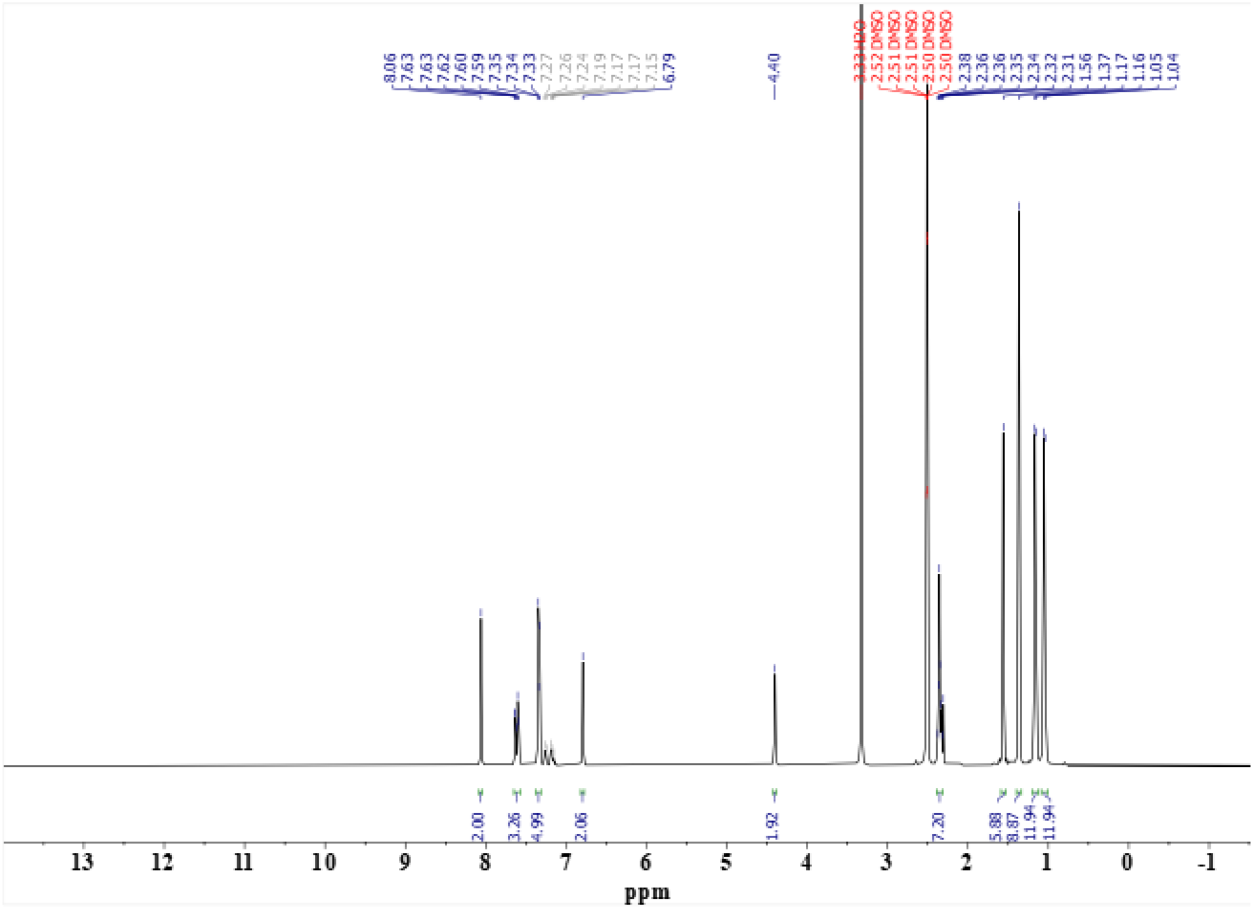

^{1}H NMR spectrum of **Au-ester** [Solvent: CD_2Cl_2]

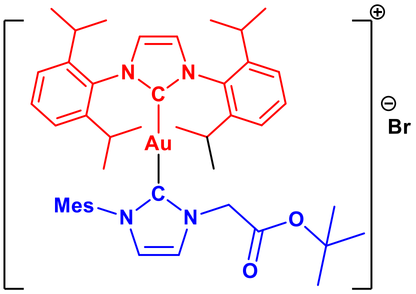

Au-ester

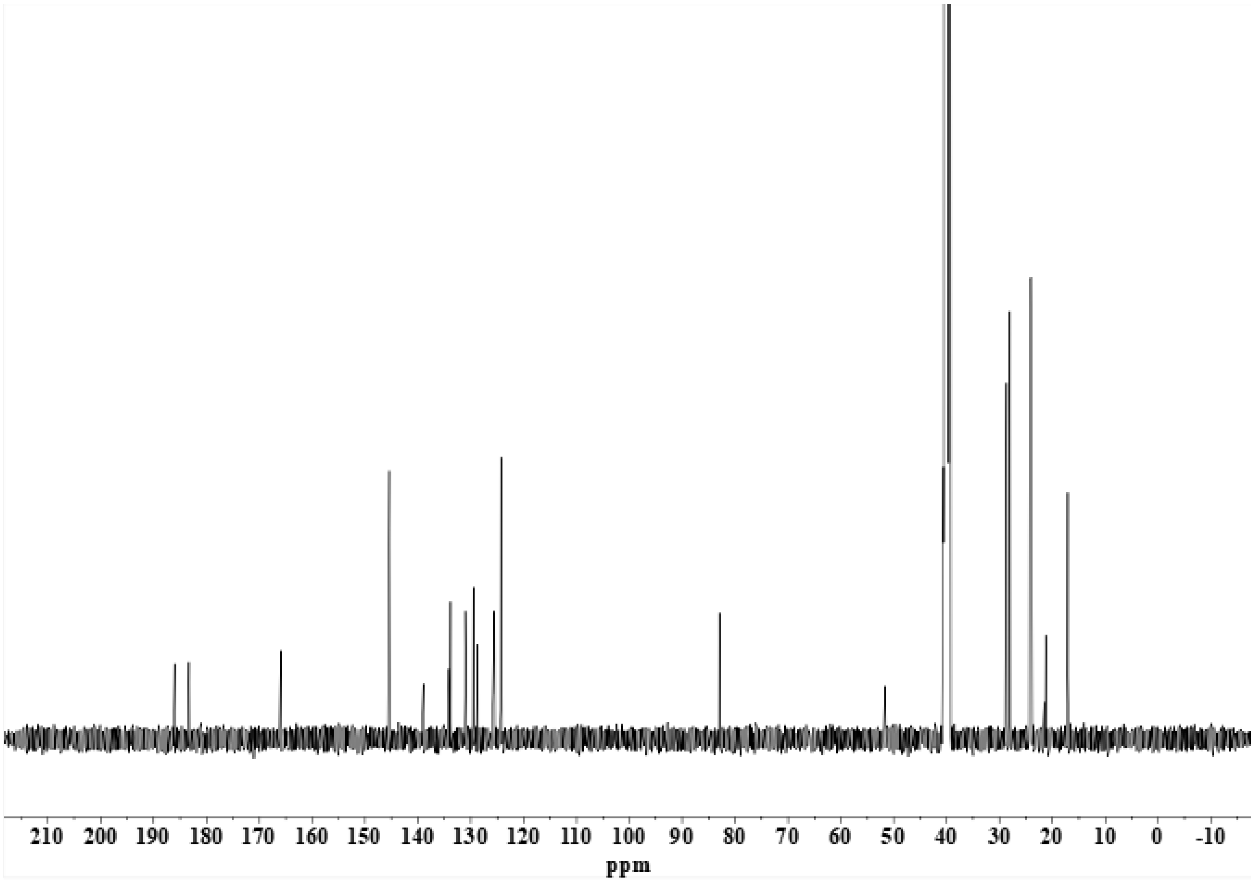

^{13}C NMR spectrum of **Au-ester** [Solvent: CD_2Cl_2]

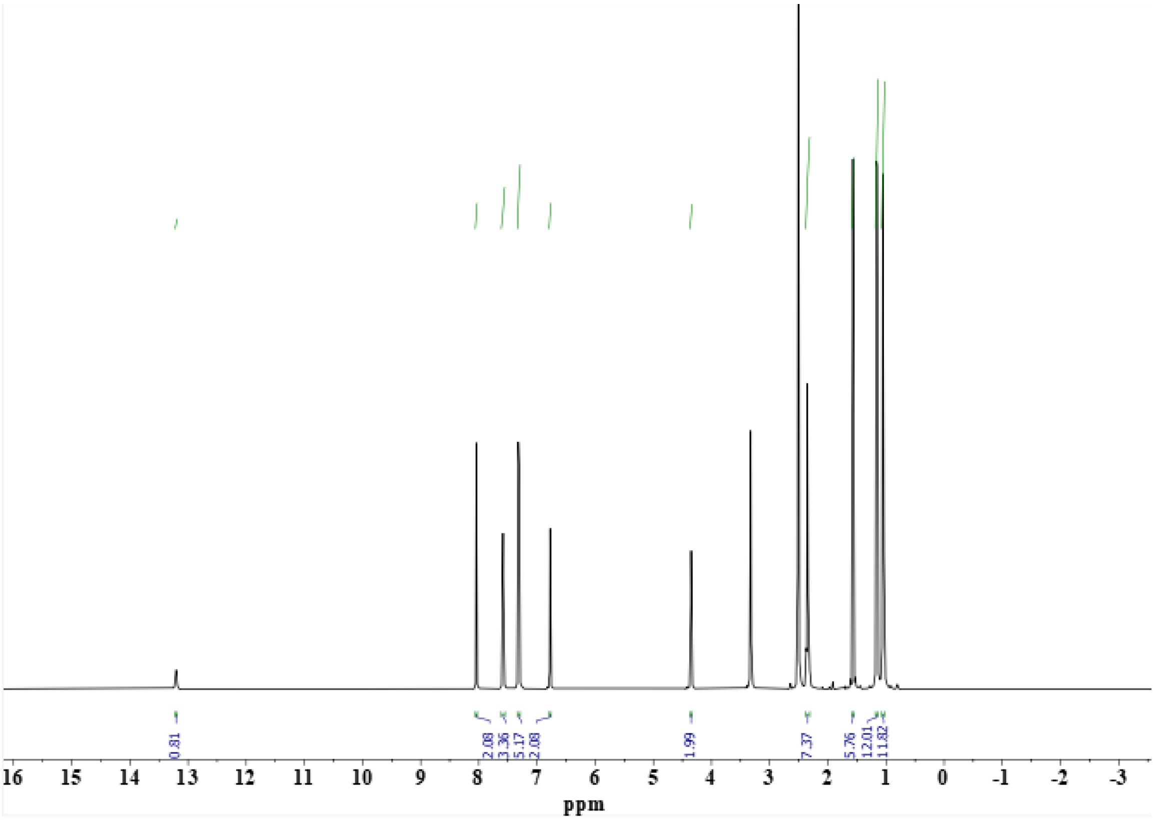

^{1}H NMR spectrum of complex **2** [Solvent: CD_2Cl_2]

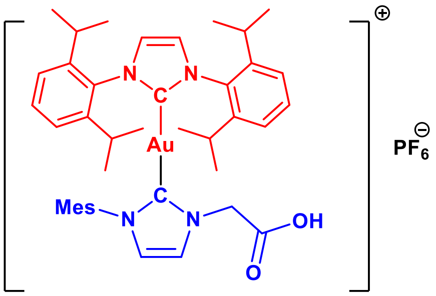

Complex 2

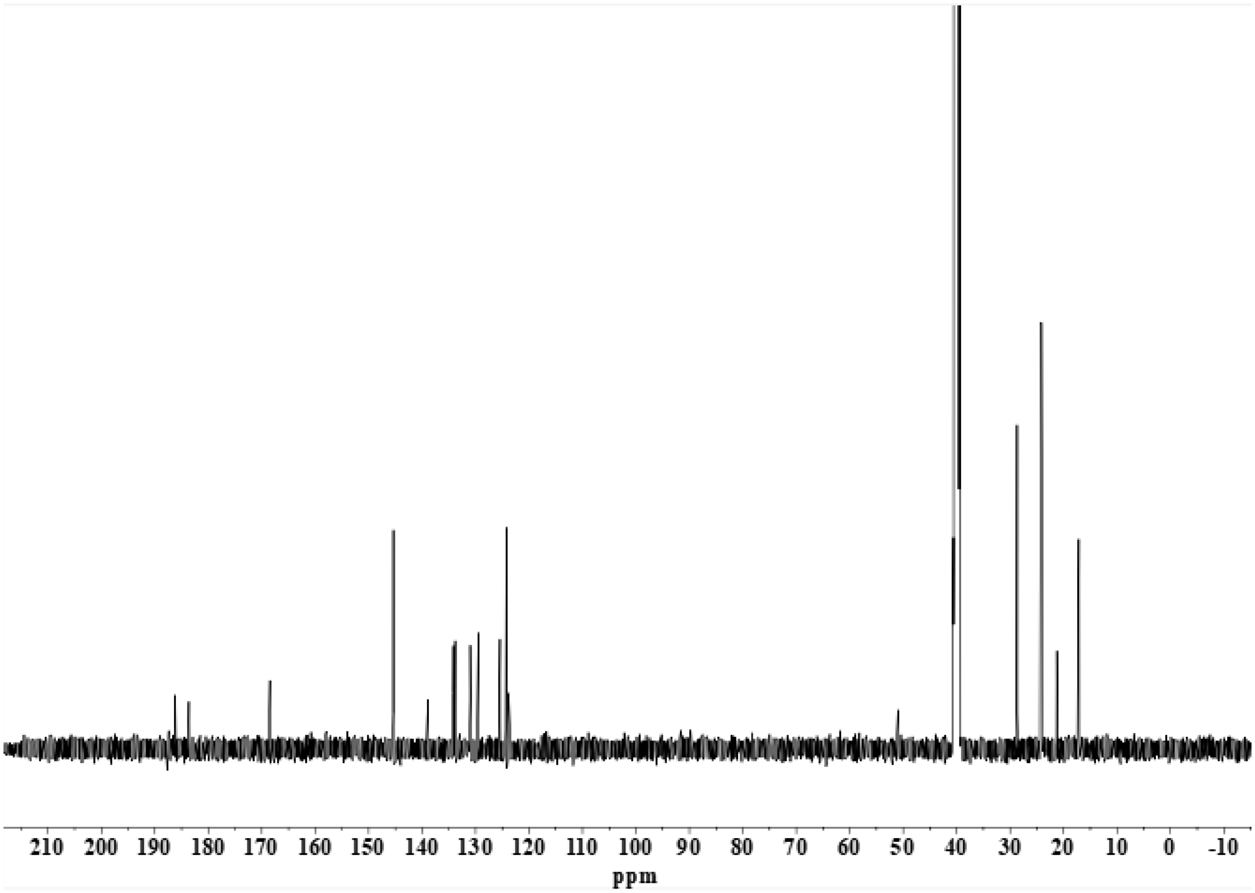

^{13}C NMR spectrum of complex **2** [Solvent: CD_2Cl_2]

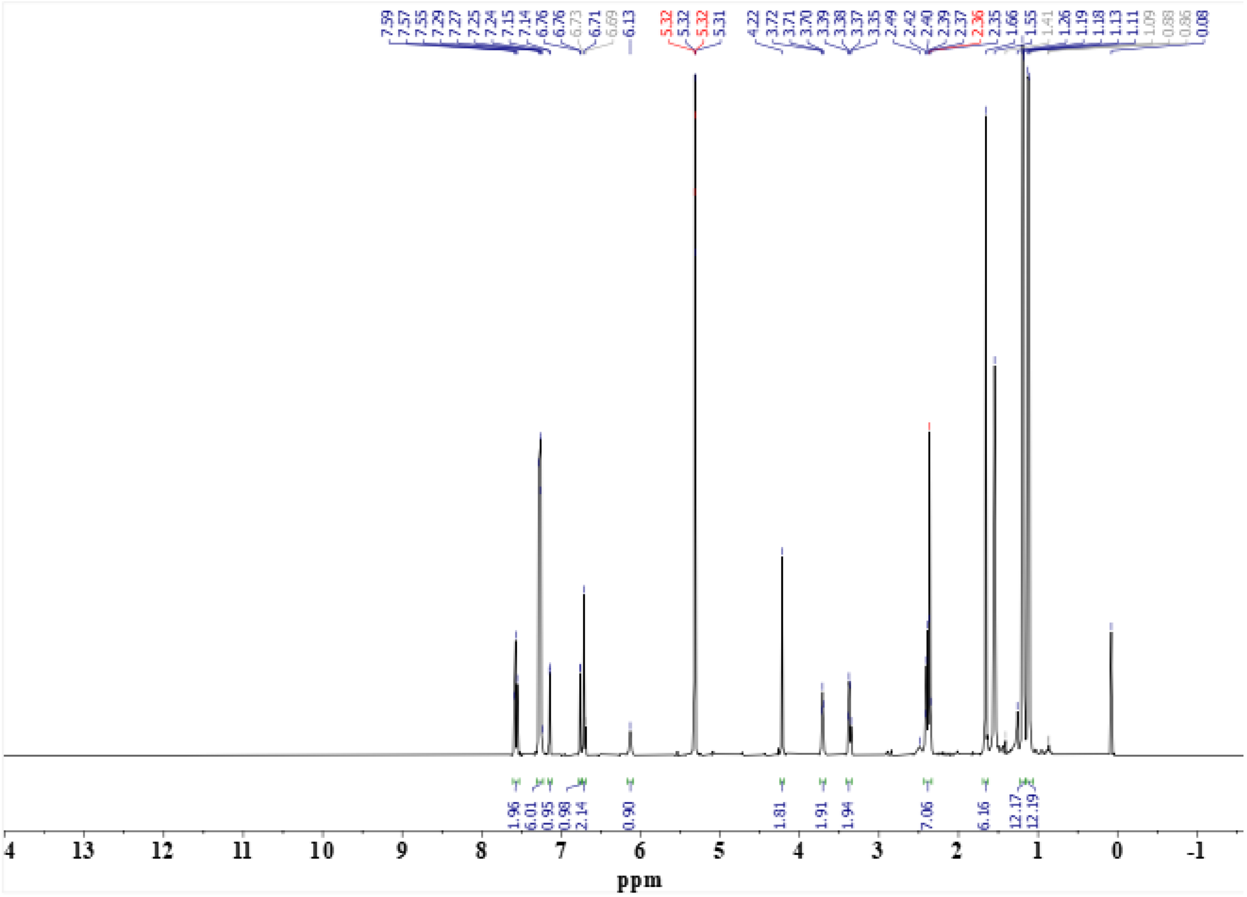

^{1}H NMR spectrum of complex **3** [Solvent: CD_2Cl_2]

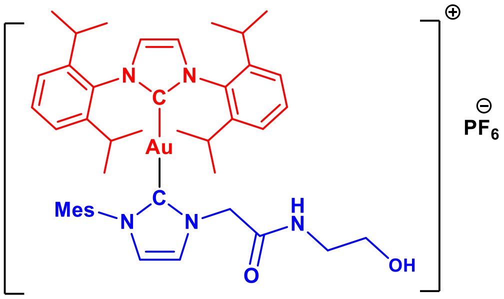

Complex 3

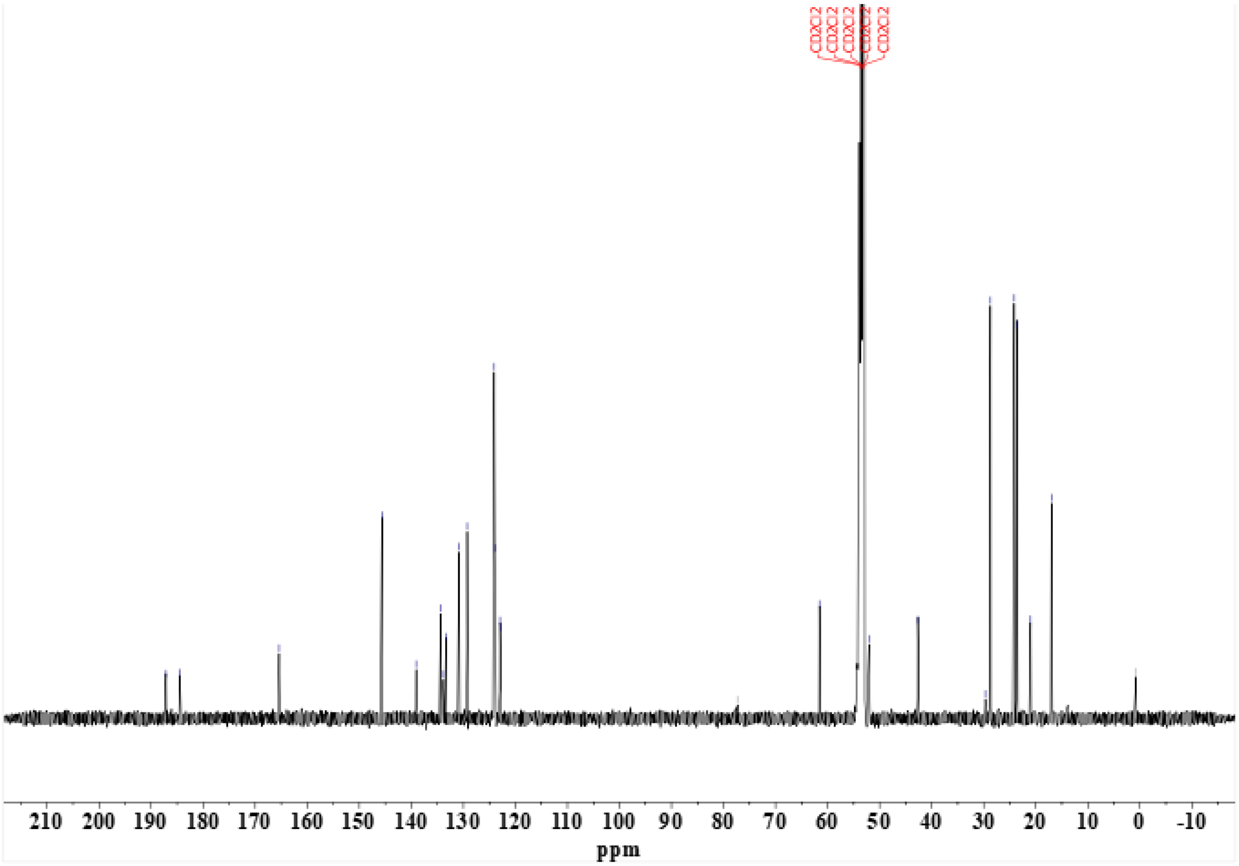

^{13}C NMR spectrum of complex **3** [Solvent: CD_2Cl_2]

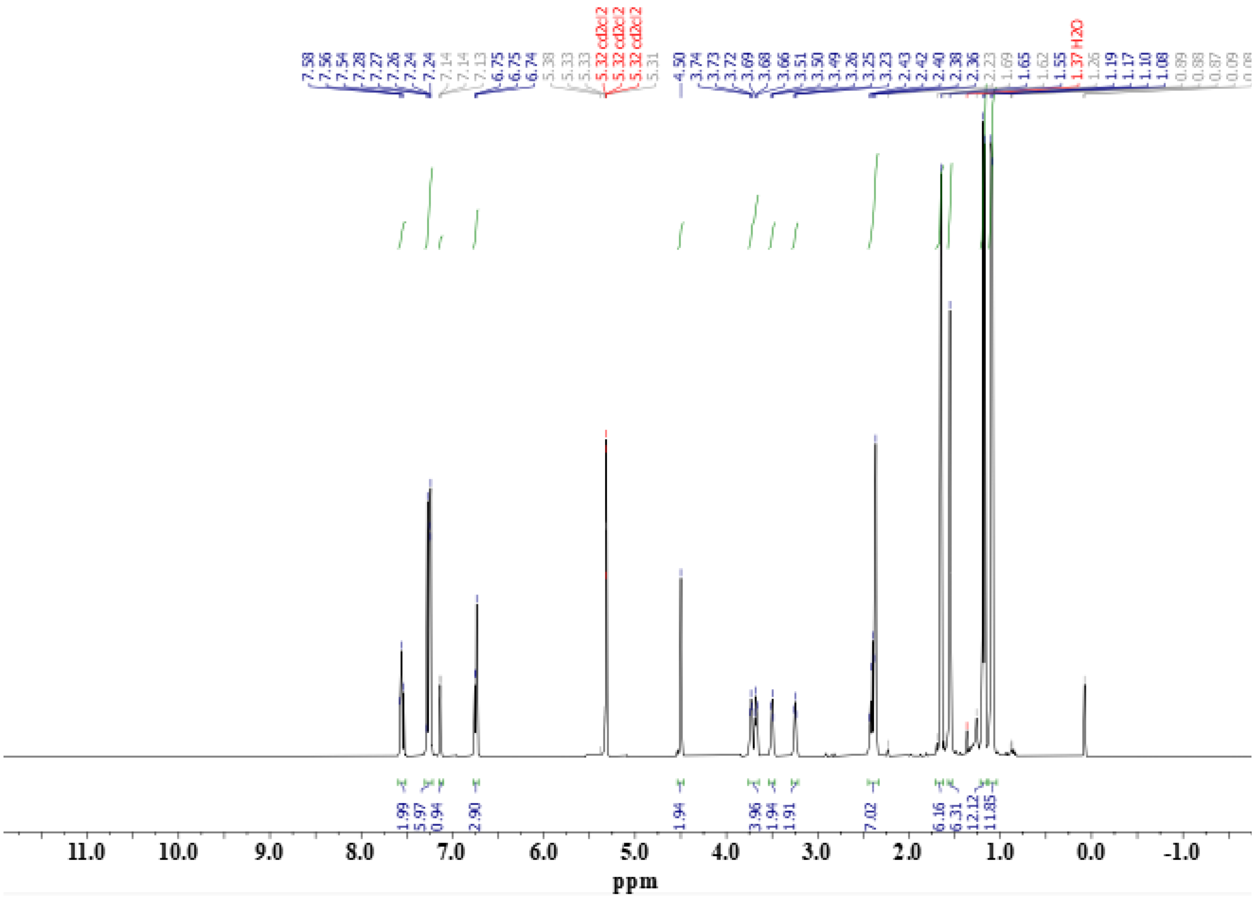

^{1}H NMR spectrum of complex **4** [Solvent: CD_2Cl_2]

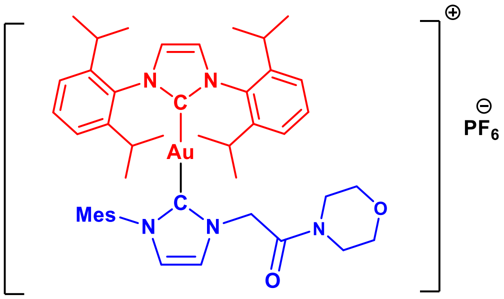

Complex 4

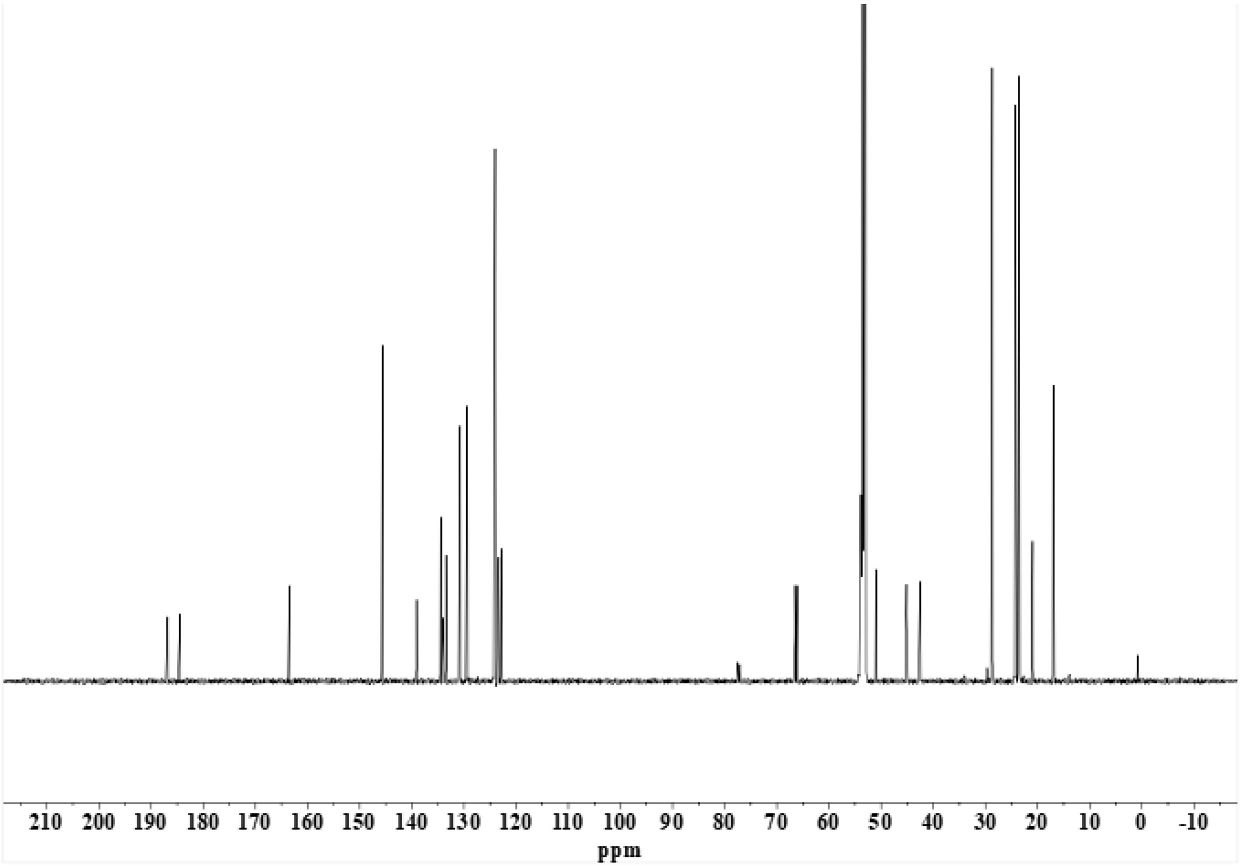

^{13}C NMR spectrum of complex **4** [Solvent: CD_2Cl_2]

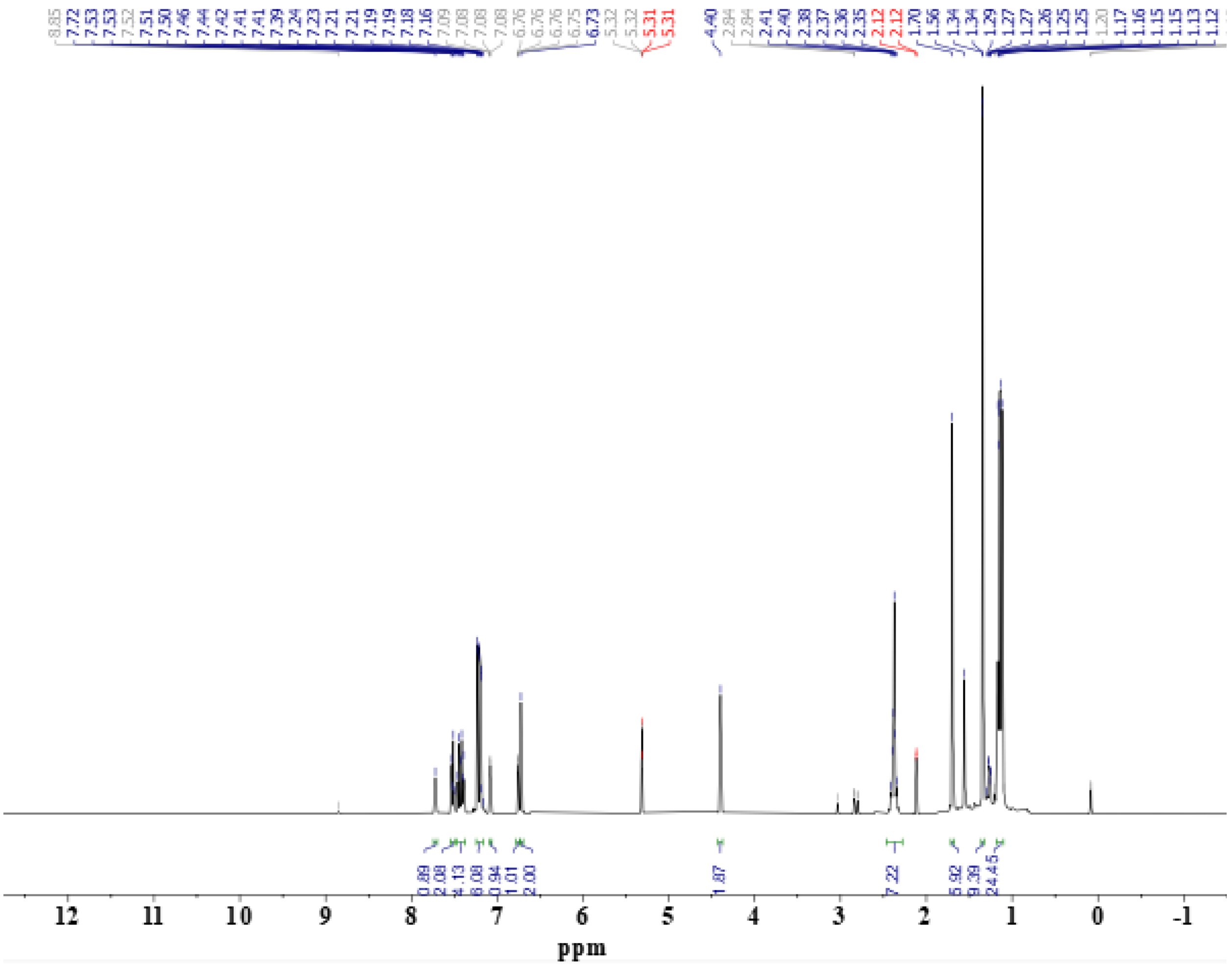

^{1}H NMR spectrum of complex **5** [Solvent: CD_2Cl_2]

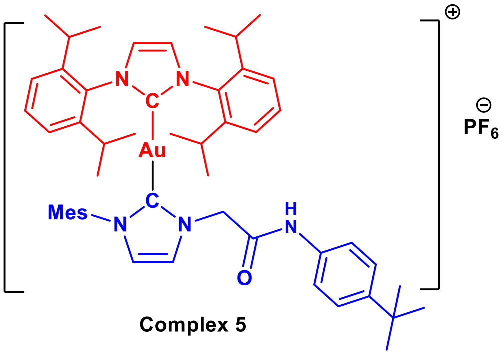

Complex 5

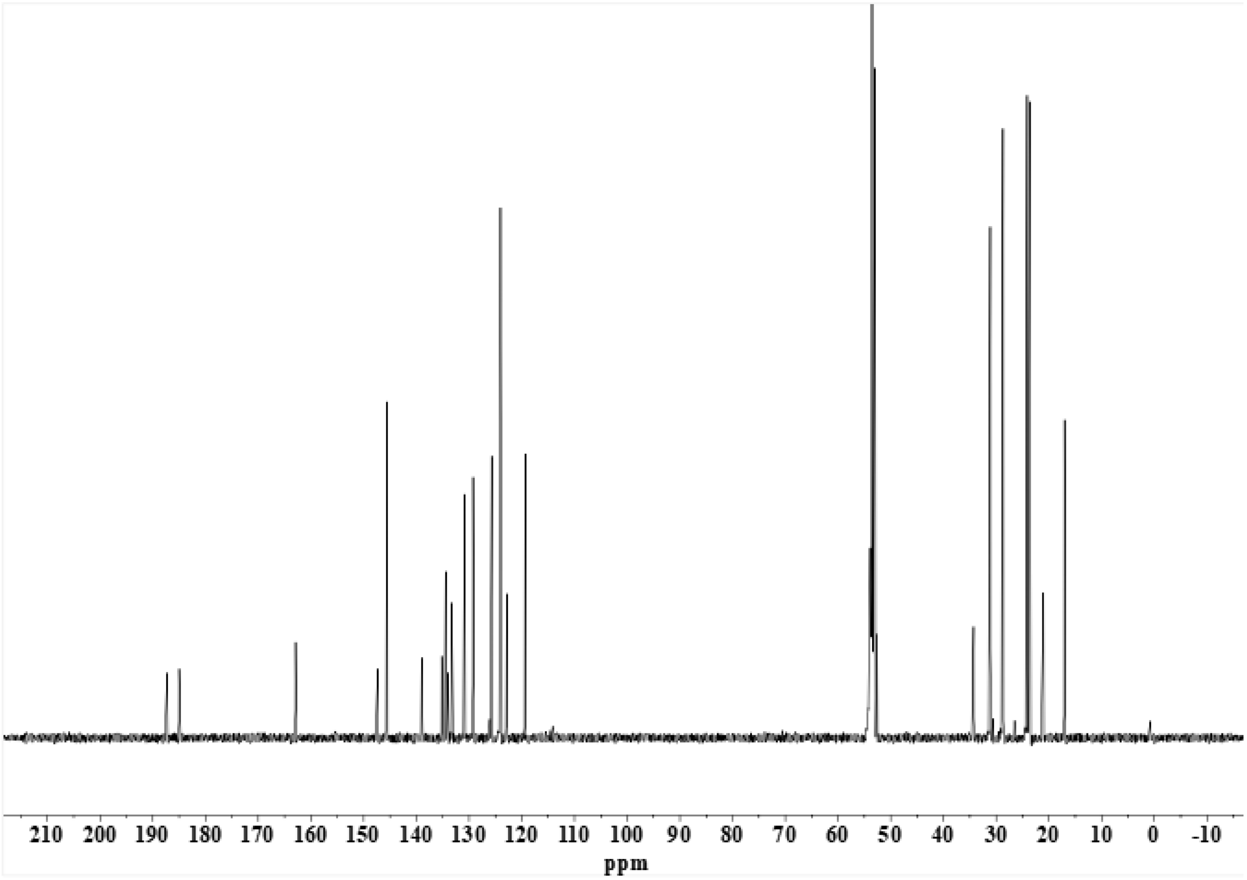

^{13}C NMR spectrum of complex **5** [Solvent: CD_2Cl_2]

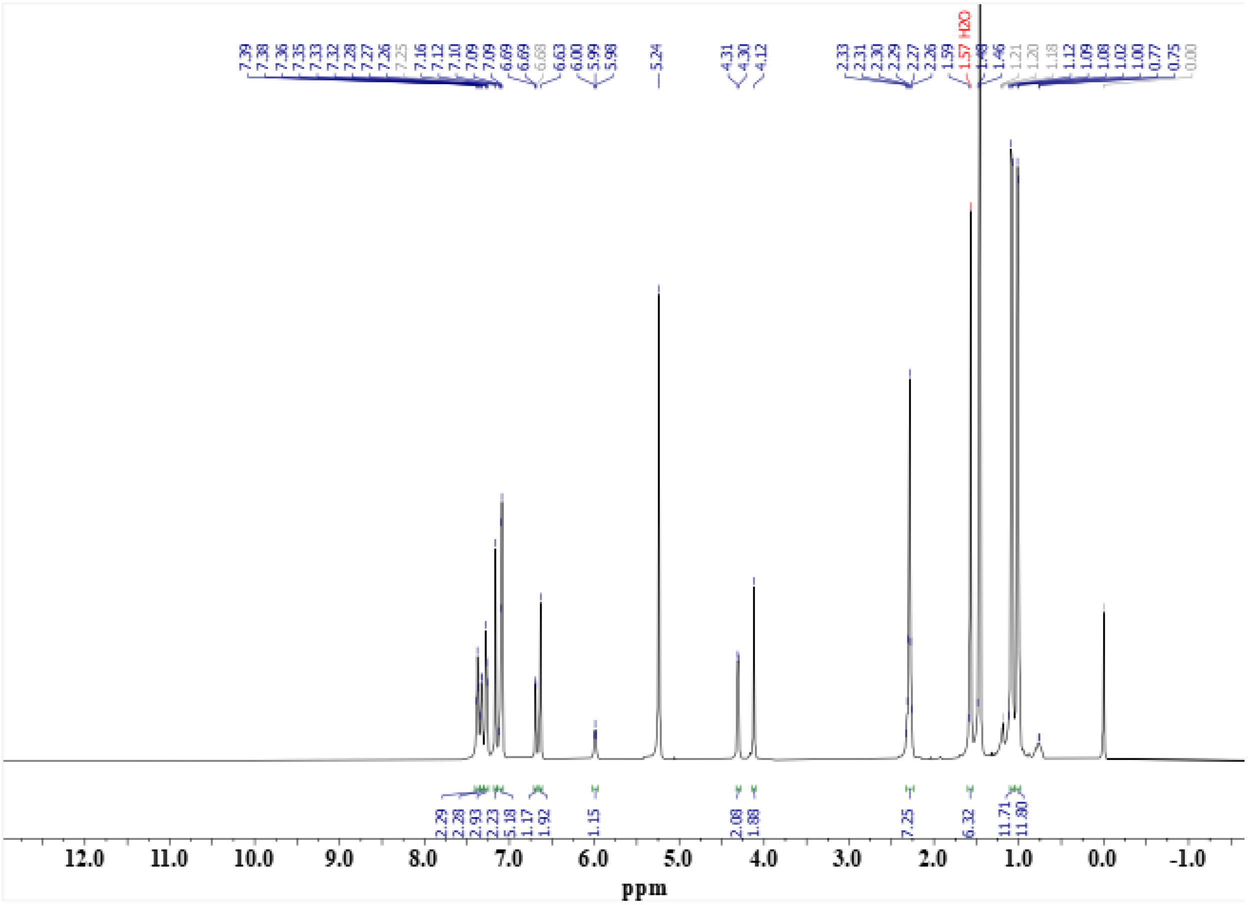

^{1}H NMR spectrum of complex **6** [Solvent: CD_2Cl_2]

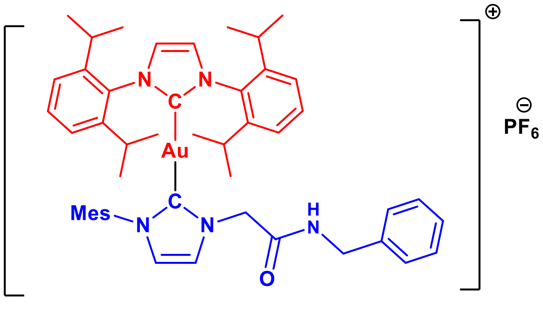

Complex 6

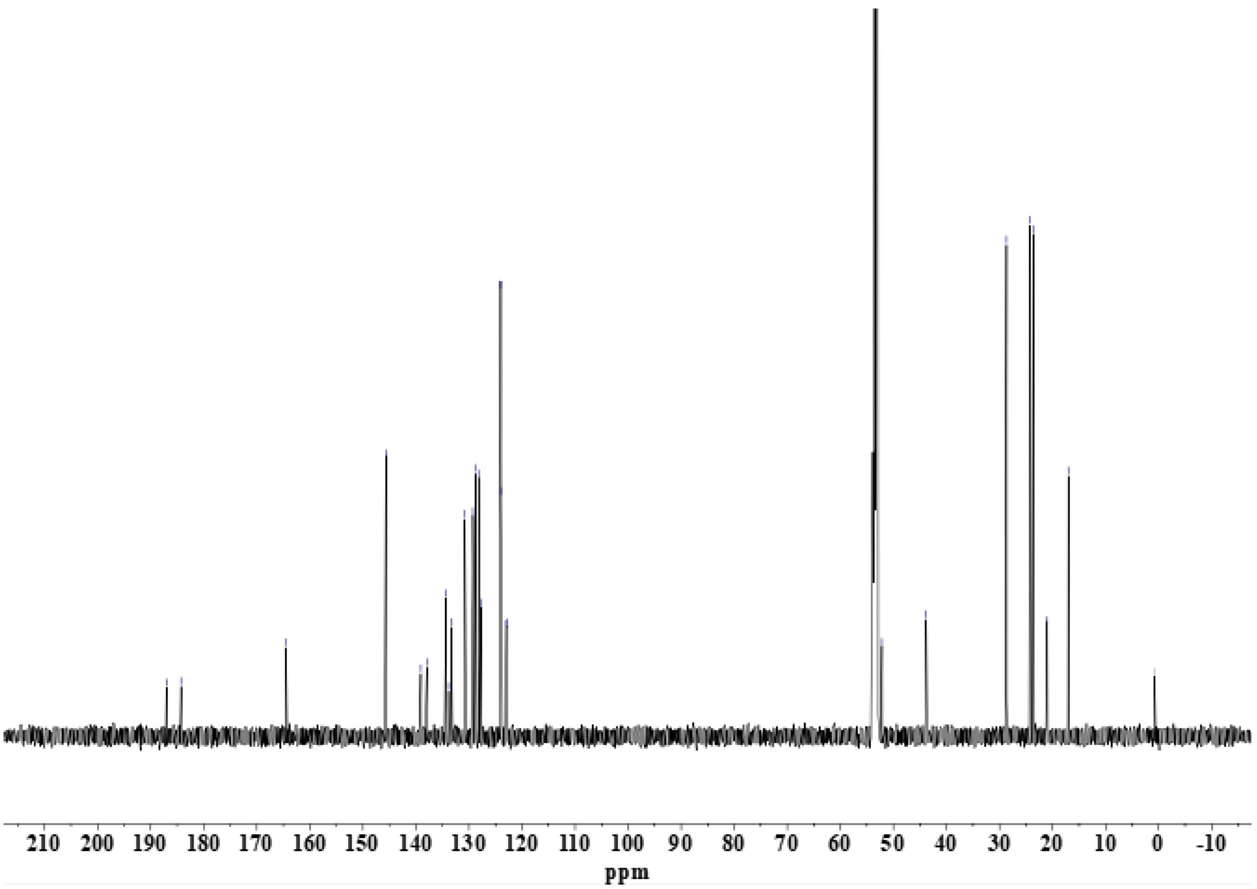

^{13}C NMR spectrum of complex **6** [Solvent: CD_2Cl_2]

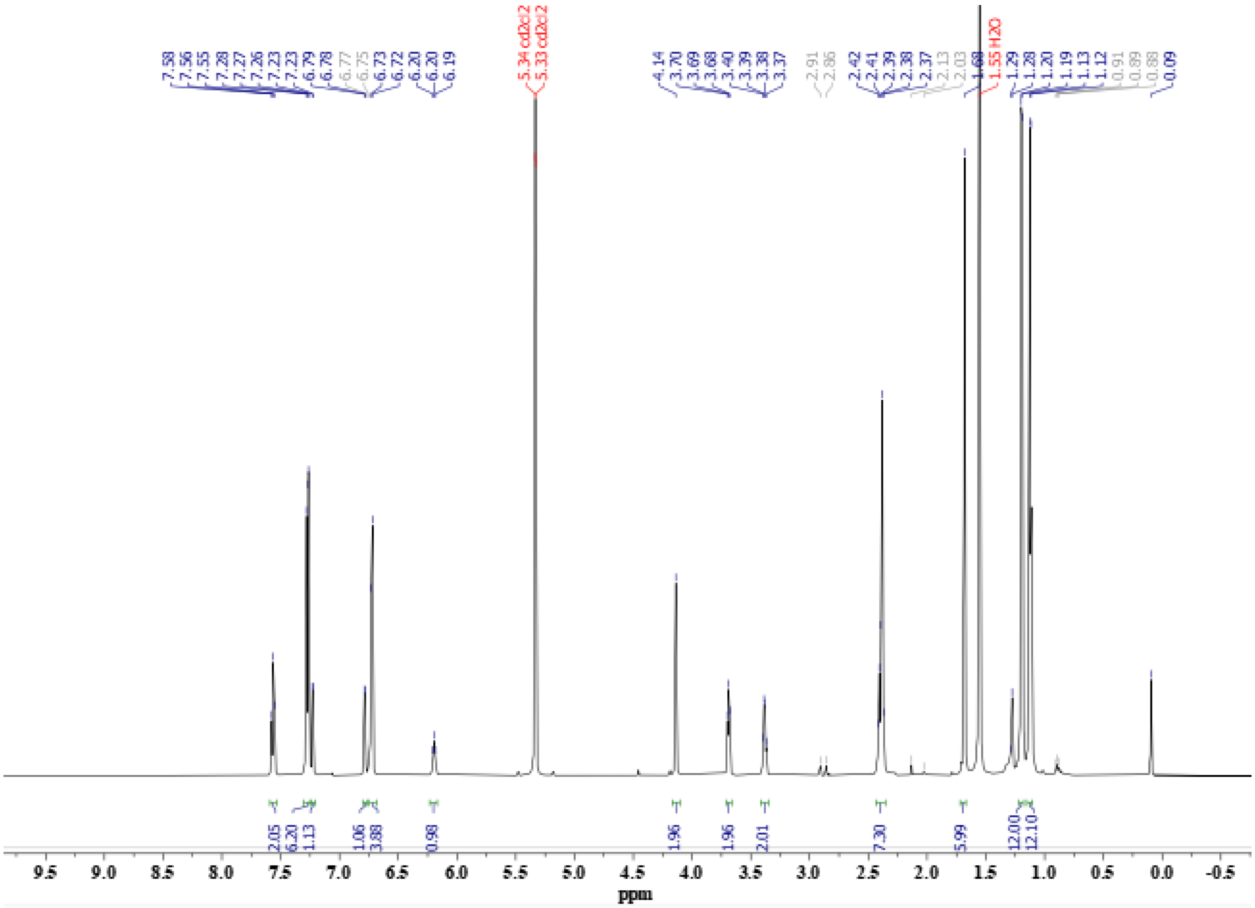

^{1}H NMR spectrum of complex **7** [Solvent: CD_2Cl_2]

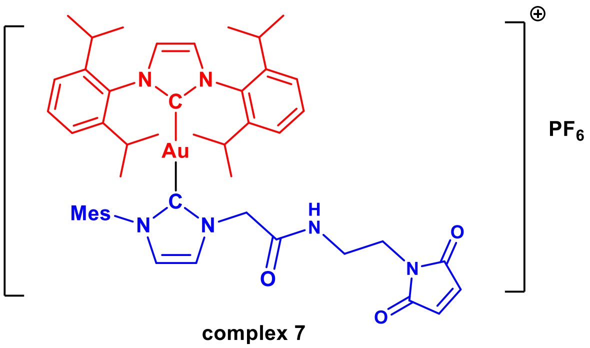

complex 7

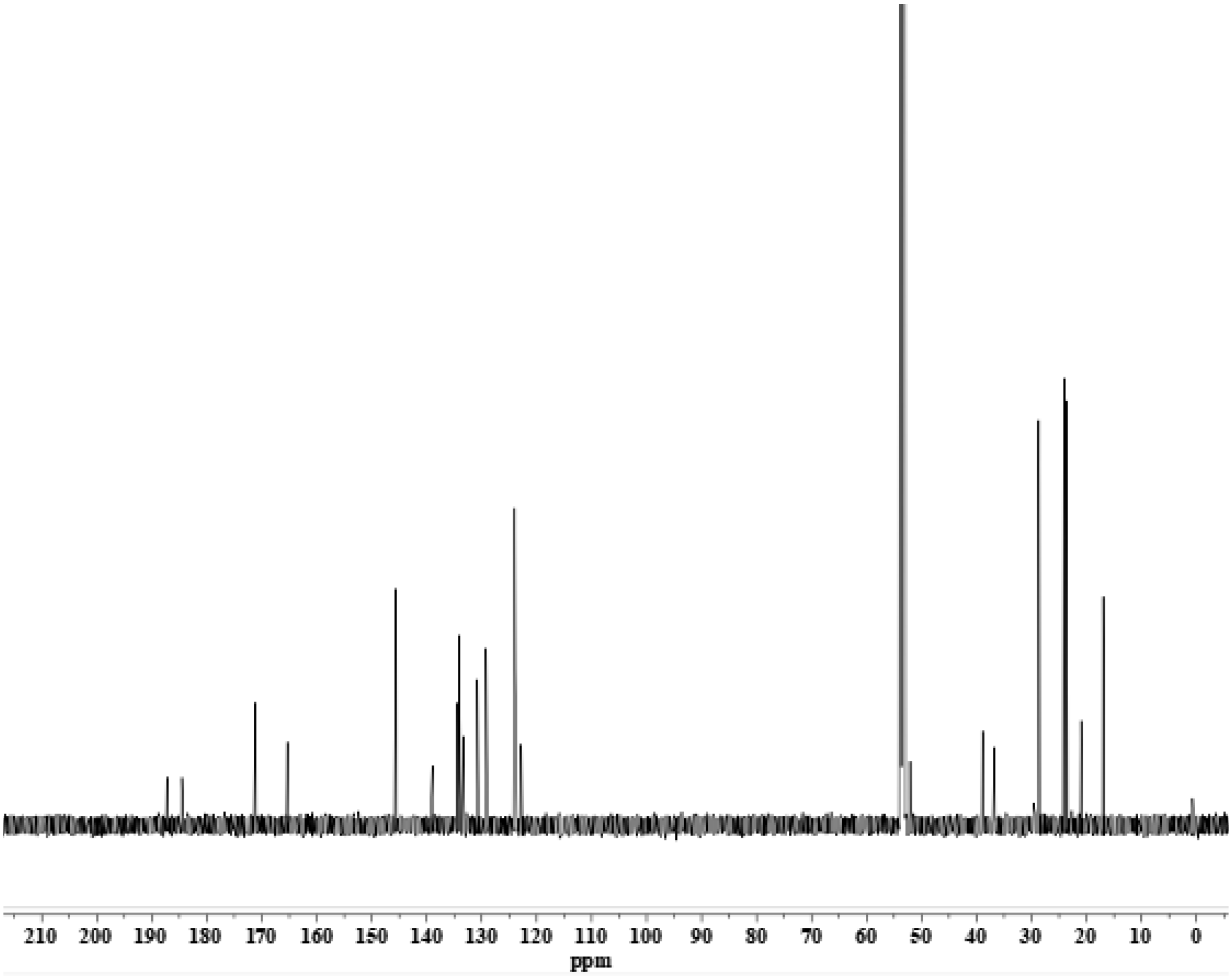

^{13}C NMR spectrum of complex **7** [Solvent: CD_2Cl_2]

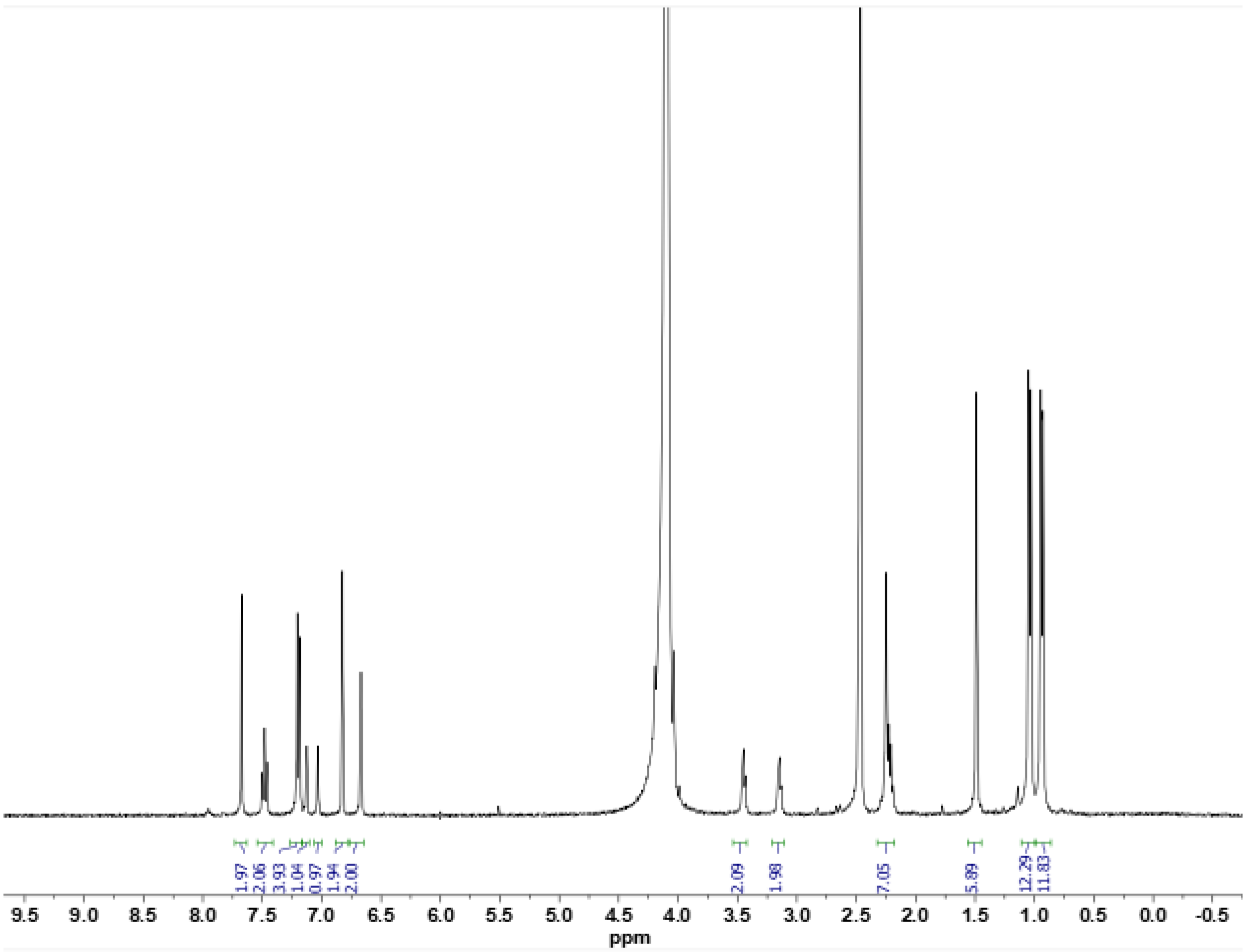

^{1}H NMR spectrum of complex **7** [Solvent: DMSO-d_6 : D_2O (3:1)]

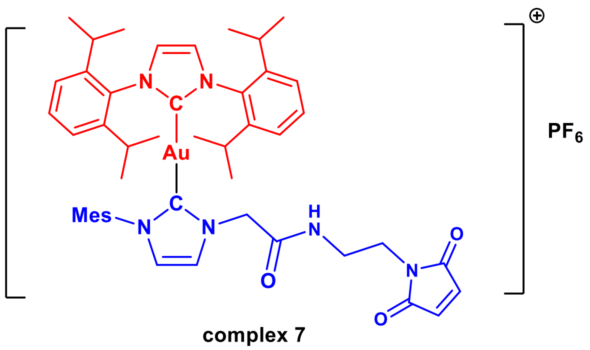

complex 7

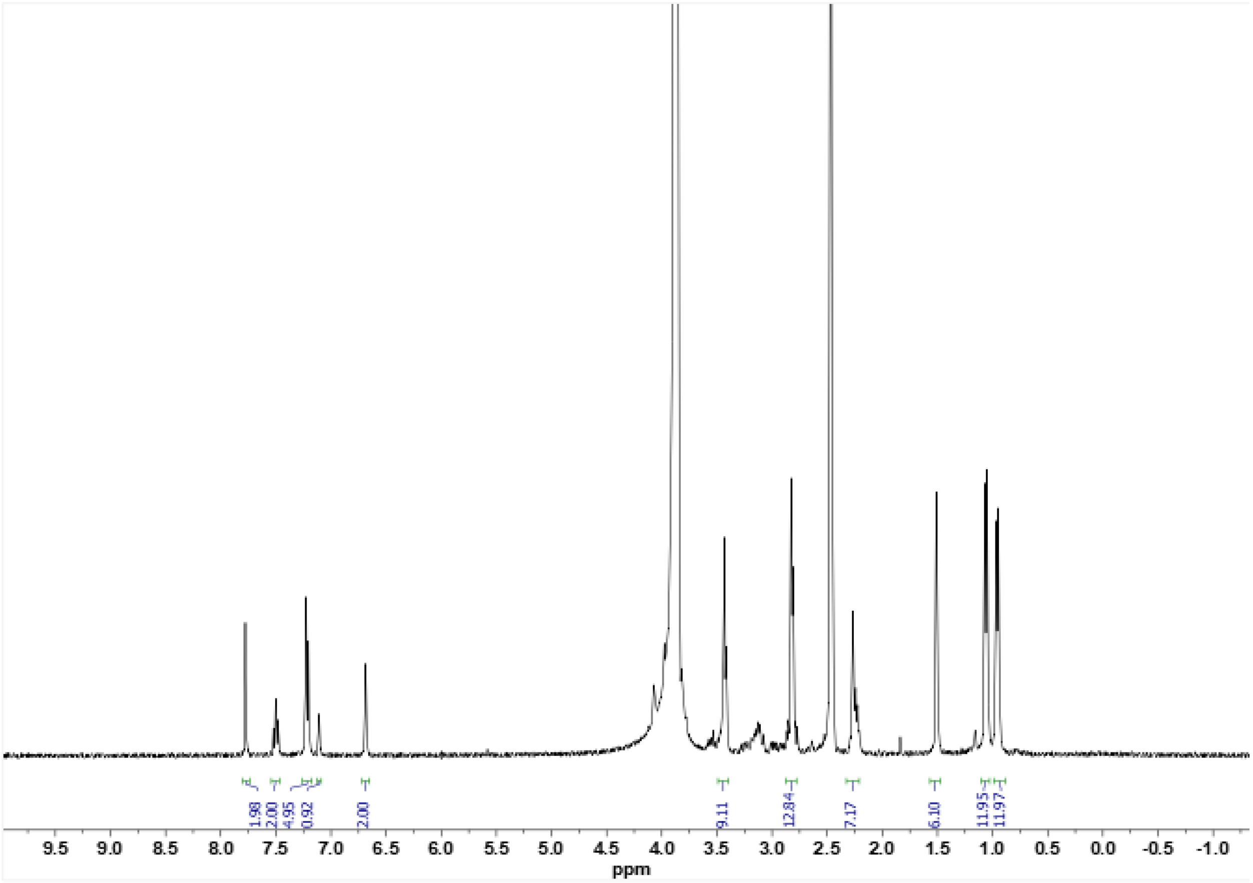

^{1}H NMR spectrum of complex **7** and cysteine **(**5 equiv.) [Solvent: DMSO-d_6 : D_2O (3:1)]

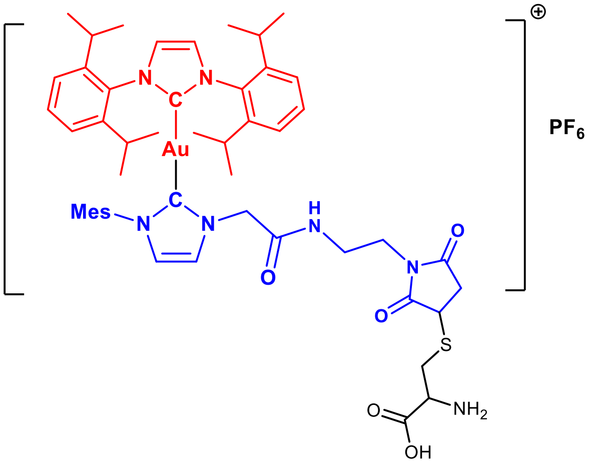

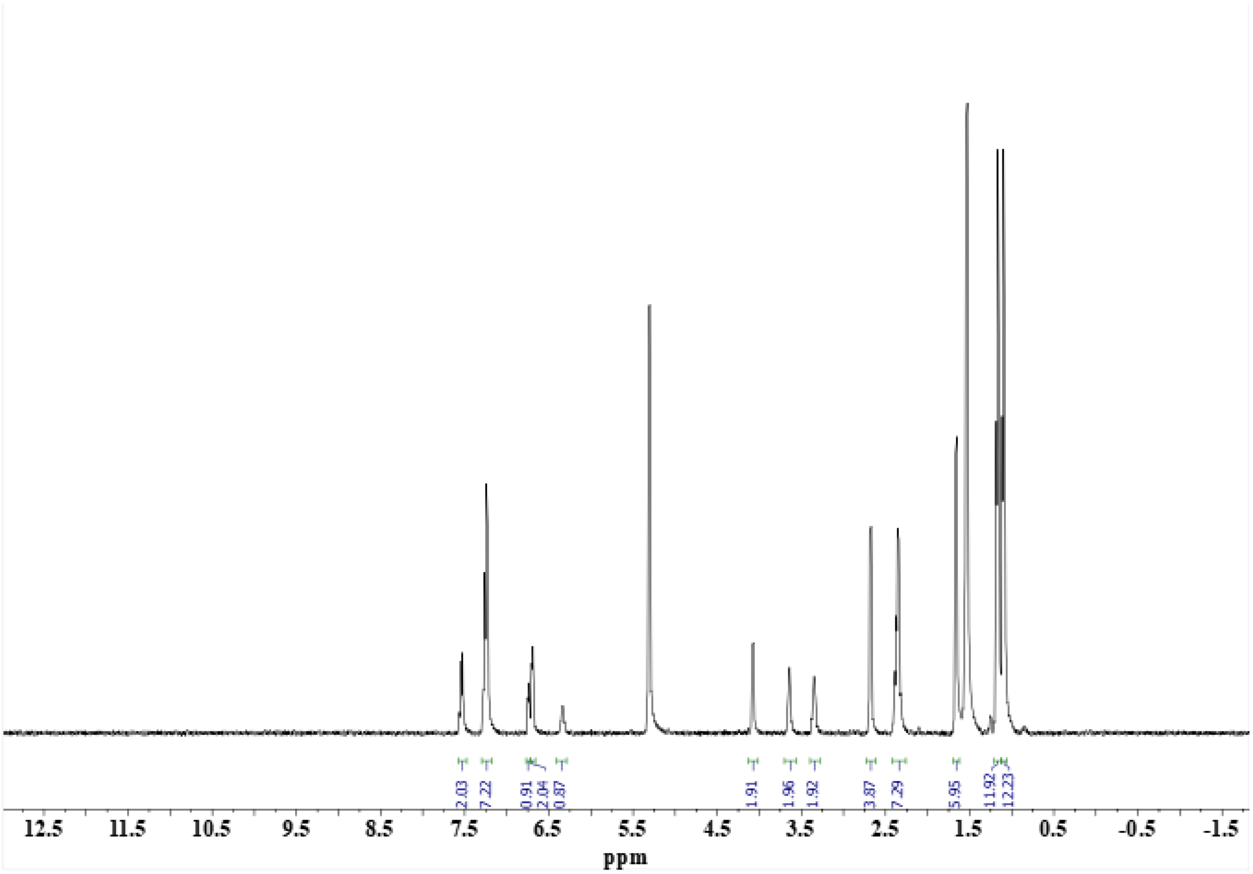

^1H NMR spectrum of complex **8** [Solvent: CD_2Cl_2]

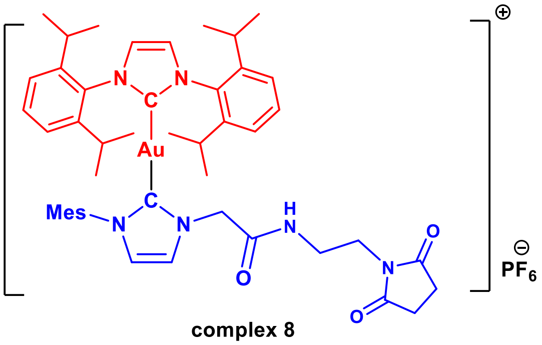

complex 8

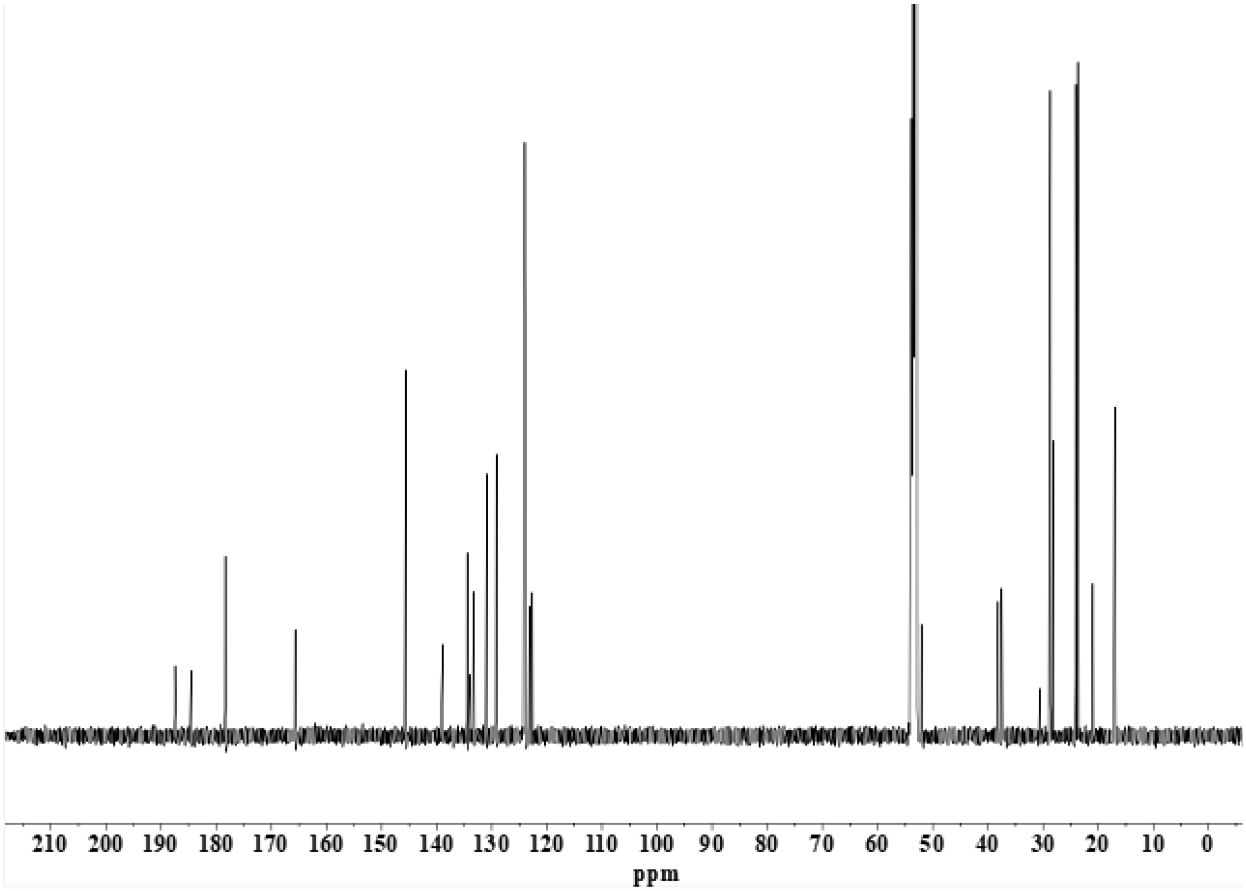

^{13}C NMR spectrum of complex **8** [Solvent: CD_2Cl_2]

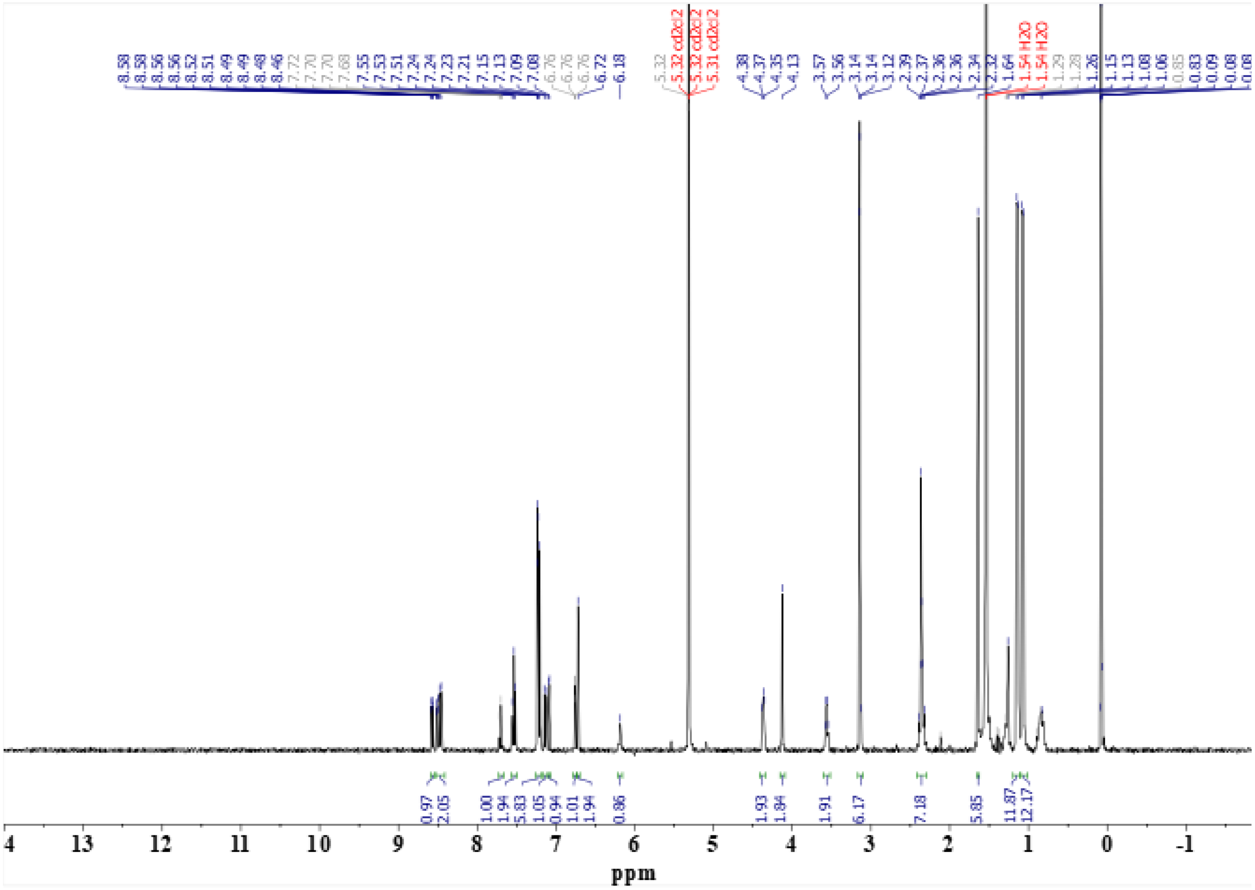

^{1}H NMR spectrum of complex **9** [Solvent: CD_2Cl_2]

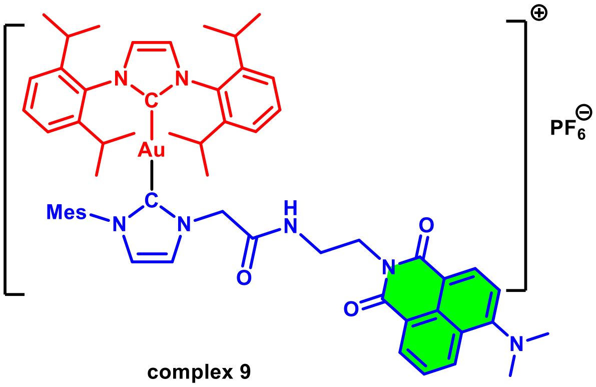

complex 9

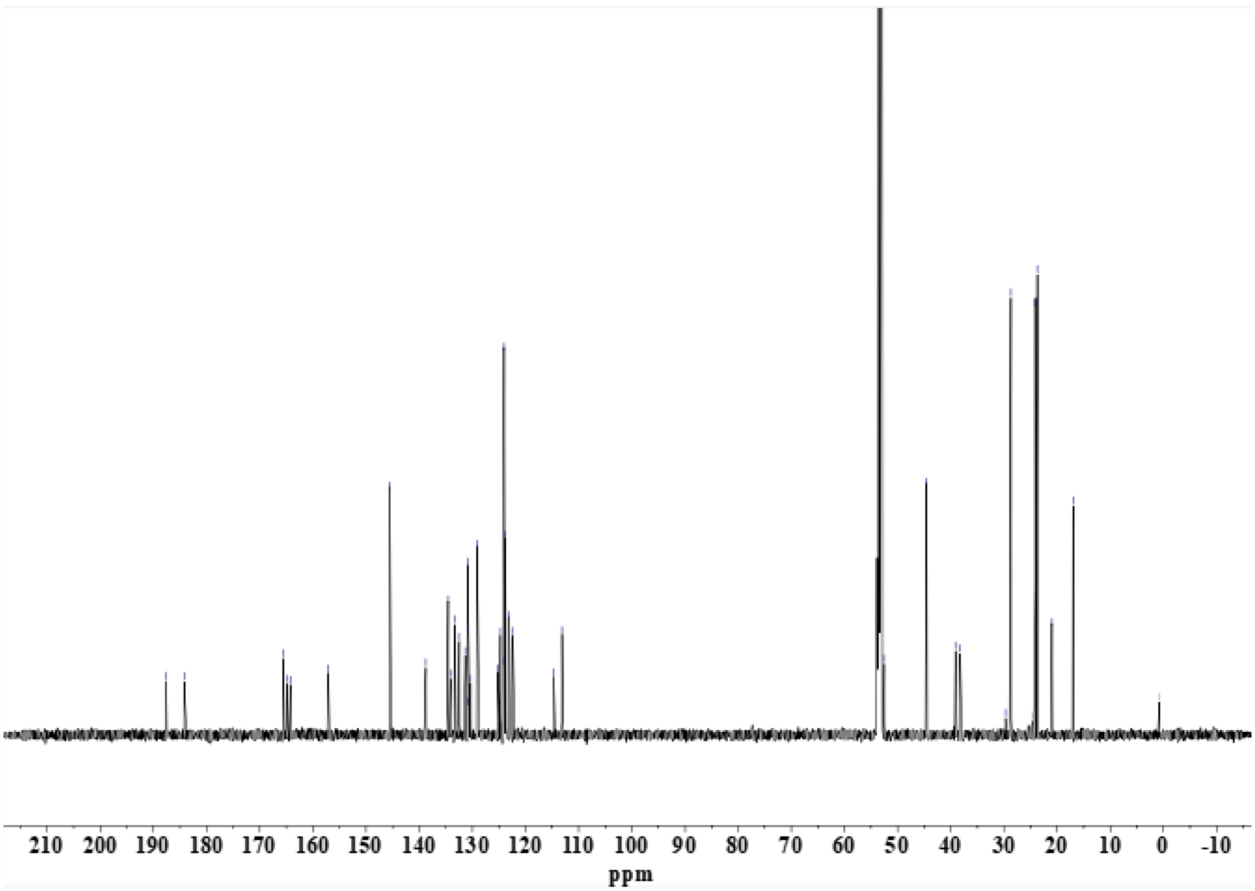

^{13}C NMR spectrum of complex **9** [Solvent: CD_2Cl_2]

7. HRMS Results:

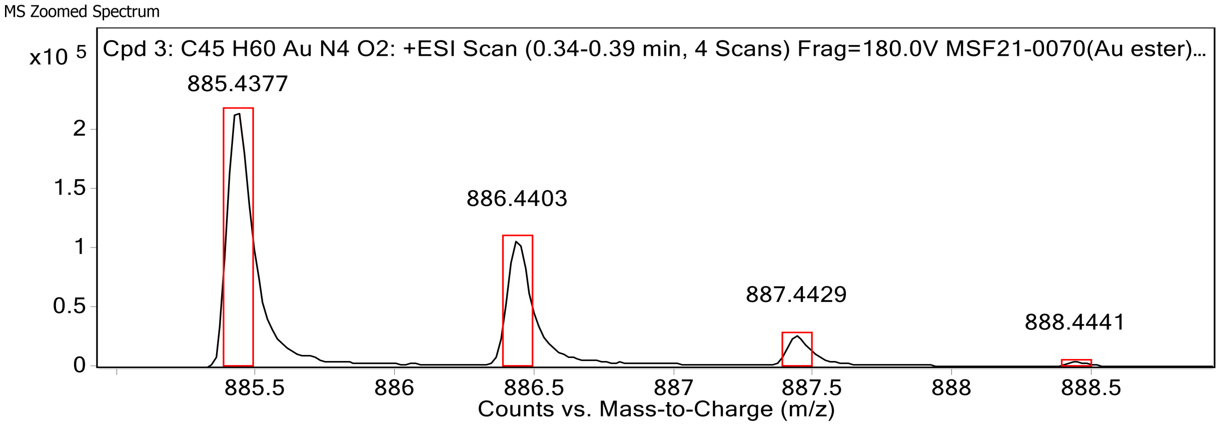

HRMS data of **Au-ester**

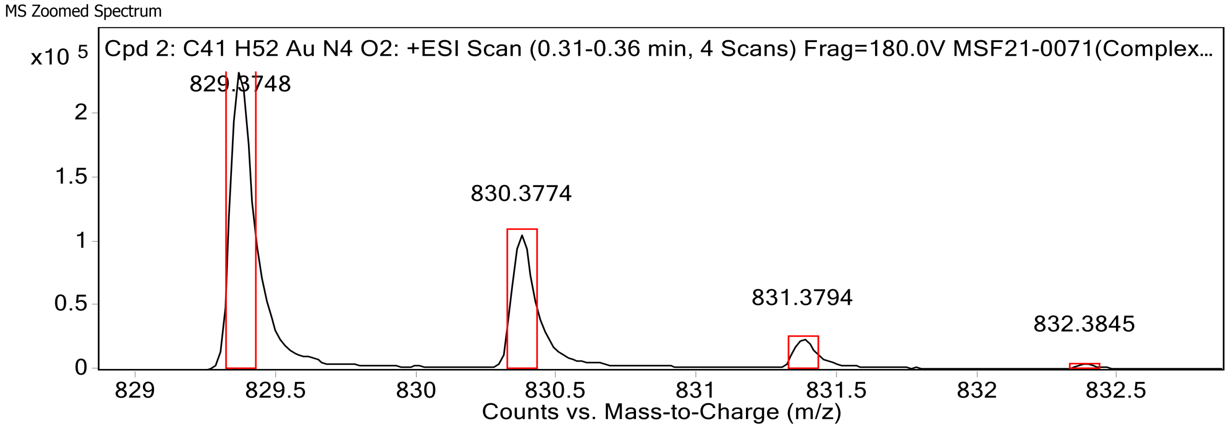

HRMS data of **complex 2**

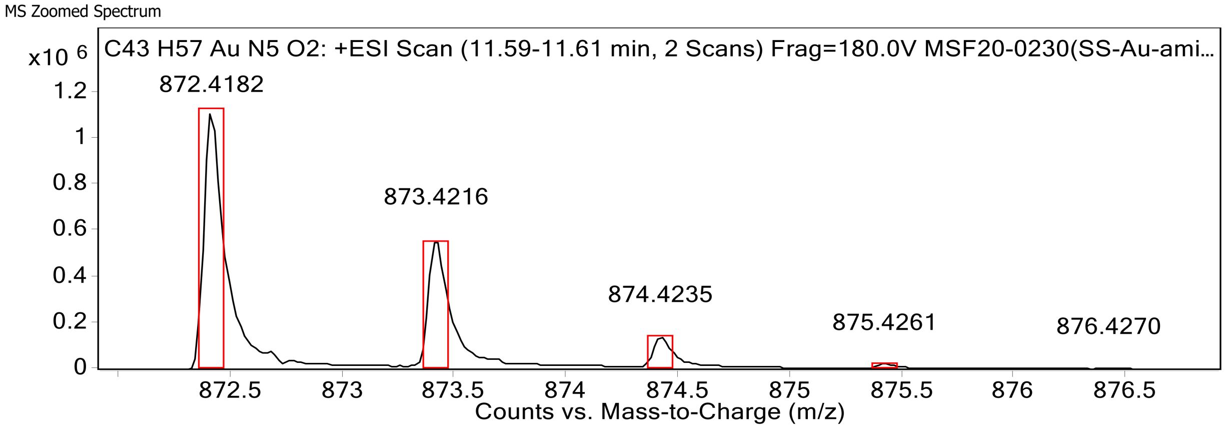

HRMS data of complex **3**

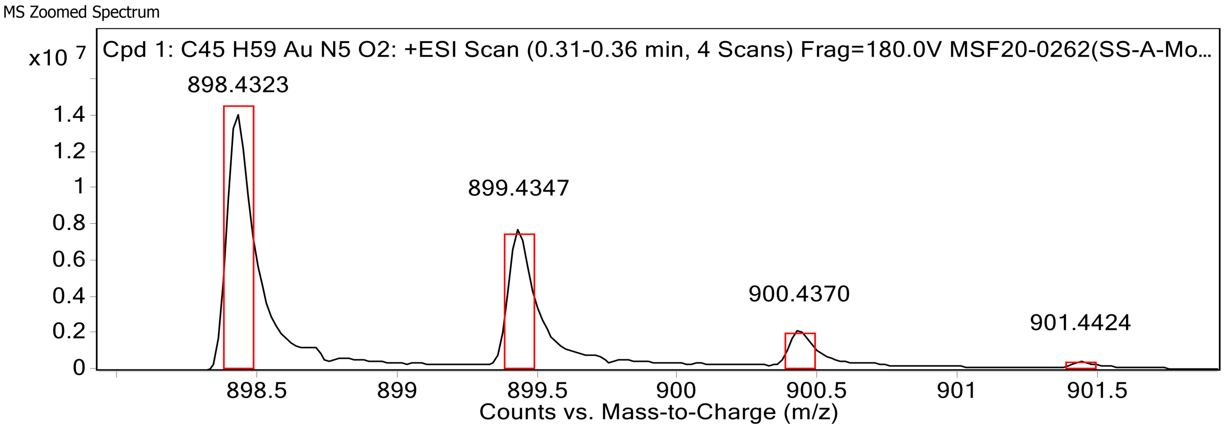

HRMS data of complex **4**

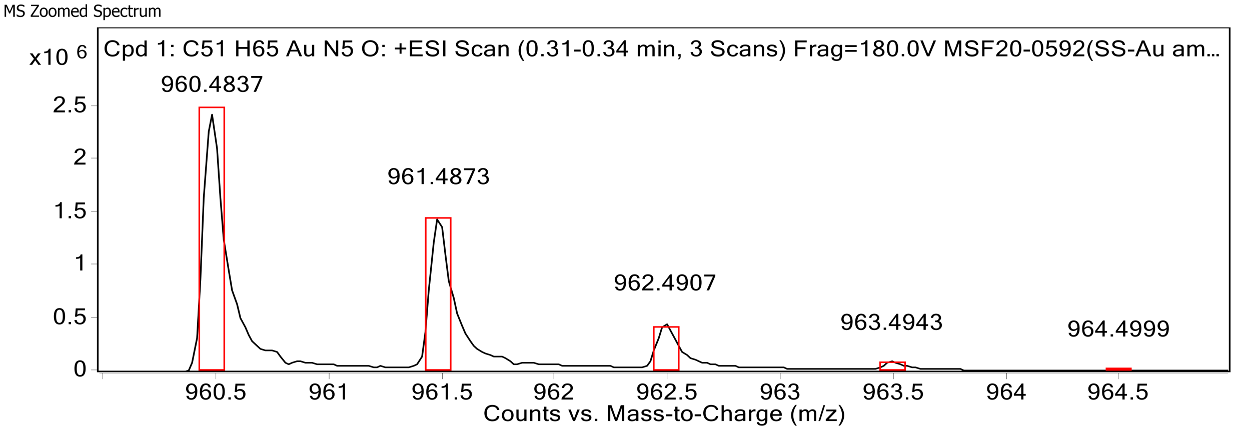

HRMS data of complex **5**

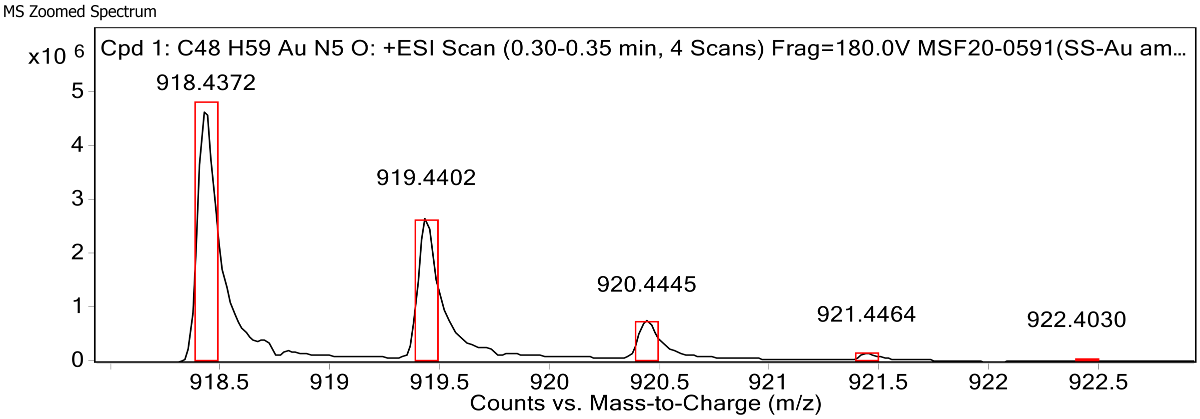

HRMS data of complex **6**

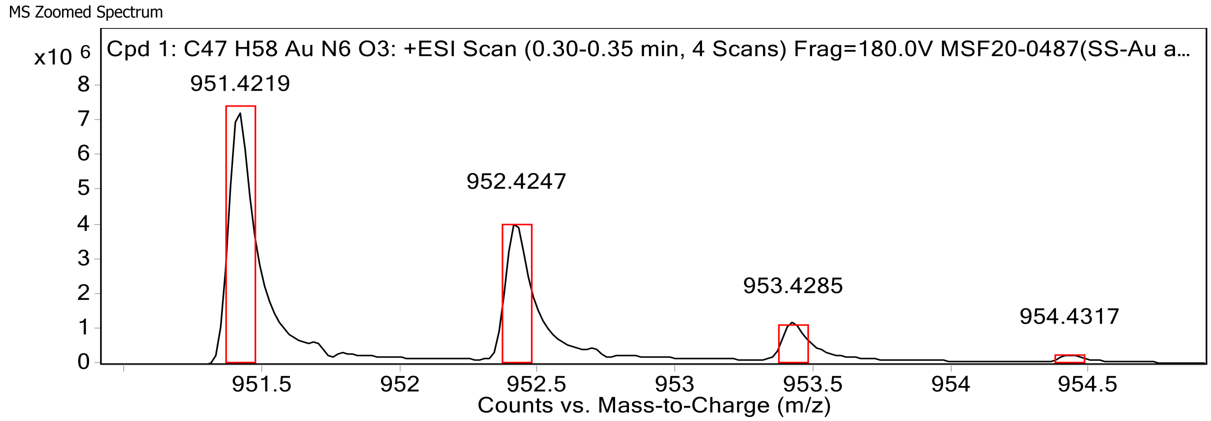

HRMS data of complex **7**

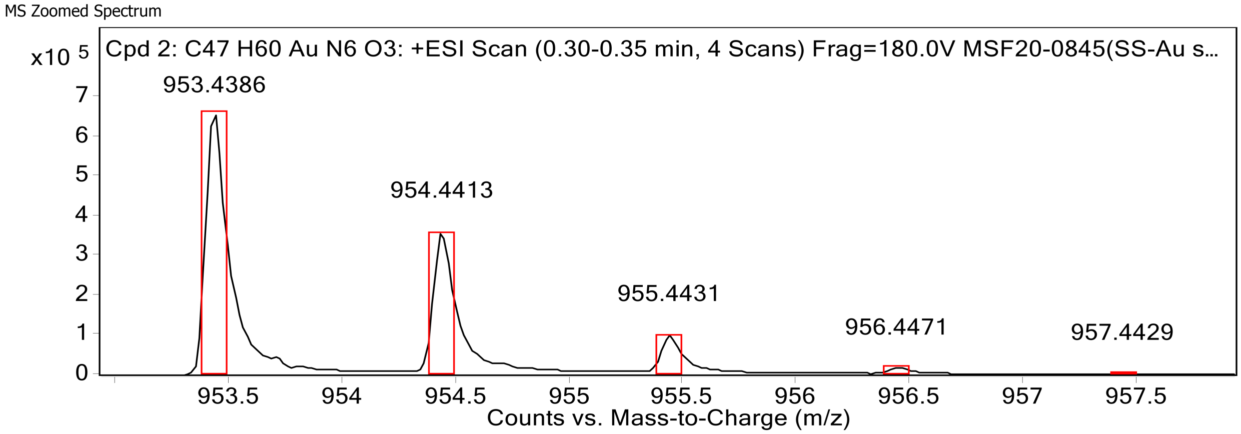

HRMS data of complex **8**

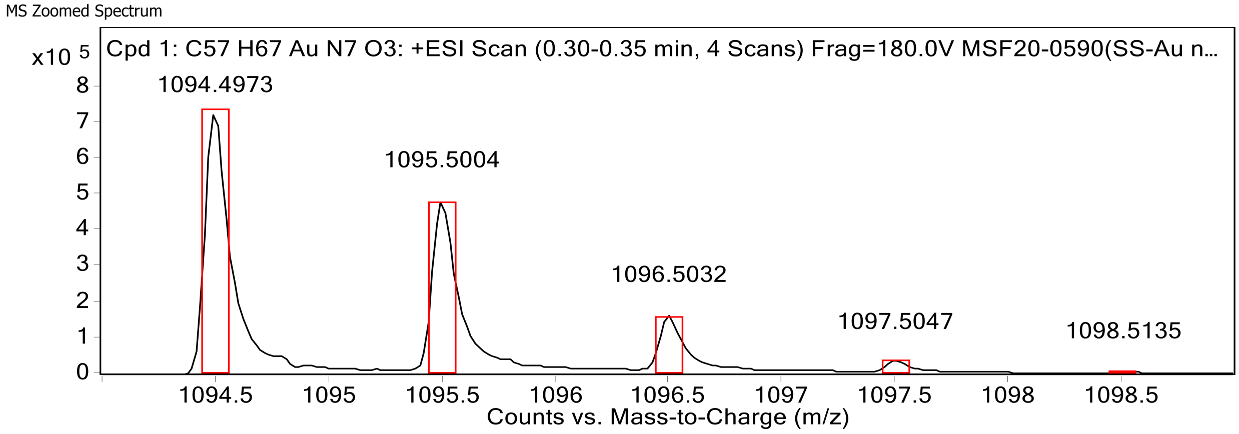

HRMS data of complex **9**

9. References

1 F. Nahra, N. V. Tzouras, A. Collado and S. P. Nolan, *Nat. Protoc.*, 2021, 1–18.
2 S. Shen, L. Dong, W. Chen, X. Zeng, H. Lu, Q. Yang and J. Zhang, *ACS Med. Chem. Lett.*, 2018, **9**, 1241–1246.
3 J. Hu, R. Allen, S. Rozinek and L. Brancaleon, *Photochem. Photobiol. Sci.*, 2017, **16**, 694–710.
4 M. Kubista, R. Sjöback, S. Eriksson and B. Albinsson, *Analyst*, 1994, **119**, 417–419.
5 M. S. Ali and H. A. Al-Lohedan, *ACS Omega*, 2020, **5**, 9131–9141.
6 T. Chatterjee, A. Pal, S. Dey, B. K. Chatterjee and P. Chakrabarti, *PLoS One*, 2012, **7**, 1–12.
7 S. Nusrat, M. K. Siddiqi, M. Zaman, N. Zaidi, M. R. Ajmal, P. Alam, A. Qadeer, A. S. Abdelhameed and R. H. Khan, *PLoS One*, 2016, **11**, 1–20.
8 A. M. Krause-Heuer, W. S. Price and J. R. Aldrich-Wright, *J. Chem. Biol.*, 2012, **5**, 105–113.
9. CrystalClear 1.40 (2008). Rigaku Americas Corportion, The Woodlands, TX.
10. L. Palatinus and G. Chapuis, *J. Appl. Crystallogr.*, 2007, **40**, 786–790.
11. G. M. Sheldrick, *Acta Cryst. C*, 2015, **71**, 9-18.
12. A. L. Spek, *Acta Cryst. D*, 2009, **65**, 148-155.
13. L. J. J. Farrugia, *Appl. Cryst.*, 1999, **32**, 837-838.
14. $R_w(F^2) = \{\Sigma w(|F_o|^2 - |F_c|^2)^2/\Sigma w(|F_o|)^4\}^{1/2}$ where w is the weight given each reflection. $R(F) = \Sigma(|F_o| - |F_c|)/\Sigma|F_o|\}$ for reflections with $F_o > 4(\sigma(F_o))$. $S = [\Sigma w(|F_o|^2 - |F_c|^2)^2/(n - p)]^{1/2}$, where n is the number of reflections and p is the number of refined parameters.
15. A. J. C. Wilson, *International Tables for X-ray Crystallography Vol. C*. Boston: Kluwer Academic Press, 1992, Tables 4.2.6.8 and 6.1.1.4.
16. G. M. Sheldrick, (1994). *SHELXTL/PC (Version 5.03)*. Siemens Analytical X-ray Instruments, Inc., Madison, Wisconsin, USA.
17. CrysAlisPro. Rigaku Oxford Diffraction (2019). CrysAlisPro Software System, 1.171.40.53.
18. G. M. Sheldrick, *Acta Cryst. A*, 2015, **71**, 3-8.
19. O. V. Dolomanov, L. J. Bourhis, R. J. Gildea, J. A. K. Howard, and H. Puschmann, *J. Appl. Cryst.*, 2009, **42**, 339-341.
20. P. Van Der Sluis and A. L. Spek, *Acta Crystallogr. Sect. A*, 1990, **46**, 194–201.
